# Management of Preschool Wheezing: Guideline from the Emilia-Romagna Asthma (ERA) Study Group

**DOI:** 10.3390/jcm11164763

**Published:** 2022-08-15

**Authors:** Valentina Fainardi, Carlo Caffarelli, Michela Deolmi, Kaltra Skenderaj, Aniello Meoli, Riccardo Morini, Barbara Maria Bergamini, Luca Bertelli, Loretta Biserna, Paolo Bottau, Elena Corinaldesi, Nicoletta De Paulis, Arianna Dondi, Battista Guidi, Francesca Lombardi, Maria Sole Magistrali, Elisabetta Marastoni, Silvia Pastorelli, Alessandra Piccorossi, Maurizio Poloni, Sylvie Tagliati, Francesca Vaienti, Giuseppe Gregori, Roberto Sacchetti, Sandra Mari, Manuela Musetti, Francesco Antodaro, Andrea Bergomi, Lamberto Reggiani, Fabio Caramelli, Alessandro De Fanti, Federico Marchetti, Giampaolo Ricci, Susanna Esposito

**Affiliations:** 1 Pediatric Clinic, Department of Medicine and Surgery, University of Parma, 43126 Parma, Italy; 2 Paediatric Unit, Department of Medical and Surgical Sciences of Mothers, Children and Adults, University of Modena and Reggio Emilia, 41125 Modena, Italy; 3 Pediatric Clinic, Scientific Institute for Research and Healthcare (IRCCS) Azienda Ospedaliero-Universitaria di Bologna, 40138 Bologna, Italy; 4 Paediatrics and Neonatology Unit, Ravenna Hospital, AUSL Romagna, 48121 Ravenna, Italy; 5 Paediatrics Unit, Imola Hospital, 40026 Imola, Italy; 6 Paediatric Unit, Carpi Hospital, 41012 Carpi, Italy; 7 Paediatrics and Neonatology Unit, Guglielmo da Saliceto Hospital, 29121 Piacenza, Italy; 8 Hospital and Territorial Paediatrics Unit, Pavullo, 41026 Pavullo nel Frignano, Italy; 9 Paediatrics Unit, Maggiore Hospital, 40133 Bologna, Italy; 10 Paediatrics Unit, Santa Maria Nuova Hospital, AUSL-IRCCS of Reggio Emilia, 42123 Reggio Emilia, Italy; 11 Paediatrics Unit, Sassuolo Hospital, 41049 Sassuolo, Italy; 12 Paediatrics and Paediatric Intensive Care Unit, Cesena Hospital, AUSL Romagna, 47521 Cesena, Italy; 13 Paediatrics Unit, Rimini Hospital, AUSL Romagna, 47921 Rimini, Italy; 14 Paediatric Clinic, Ferrara Hospital, 44124 Ferrara, Italy; 15 Paediatrics Unit, G.B. Morgagni—L. Pierantoni Hospital, AUSL Romagna, 47121 Forlì, Italy; 16 Primary Care Pediatricians, AUSL Piacenza, 29121 Piacenza, Italy; 17 Primary Care Pediatricians, AUSL Parma, 43126 Parma, Italy; 18 Primary Care Pediatricians, AUSL Modena, 41125 Modena, Italy; 19 Primary Care Pediatricians, AUSL Imola, 40026 Imola, Italy; 20 Pediatric Intensive Care Unit, IRCCS Azienda Ospedaliero-Universitaria di Bologna, 40138 Bologna, Italy

**Keywords:** allergen sensitization, episodic viral wheezing, multiple trigger wheezing, pediatric pulmonology, wheezing

## Abstract

Preschool wheezing should be considered an umbrella term for distinctive diseases with different observable and measurable phenotypes. Despite many efforts, there is a large gap in knowledge regarding management of preschool wheezing. In order to fill this lack of knowledge, the aim of these guidelines was to define management of wheezing disorders in preschool children (aged up to 5 years). A multidisciplinary panel of experts of the Emilia-Romagna Region, Italy, addressed twelve different key questions regarding the management of preschool wheezing. Clinical questions have been formulated by the expert panel using the PICO format (Patients, Intervention, Comparison, Outcomes) and systematic reviews have been conducted on PubMed to answer these specific questions, with the aim of formulating recommendations. The GRADE approach has been used for each selected paper, to assess the quality of the evidence and the degree of recommendations. These guidelines represent, in our opinion, the most complete and up-to-date collection of recommendations on preschool wheezing to guide pediatricians in the management of their patients, standardizing approaches. Undoubtedly, more research is needed to find objective biomarkers and understand underlying mechanisms to assess phenotype and endotype and to personalize targeted treatment.

## 1. Introduction

About one out of three children experiences wheezing during the first 3 years of life [1], but not all will develop asthma in later age [2]. Preschool wheezing should be considered an umbrella term for distinctive diseases with different observable and measurable features (phenotypes). Each phenotype may be the result of different endotypes, described by a mechanism that links clinical features to a molecular pathway. The three most common phenotypes of preschool wheeze that mostly rely on the temporal pattern of symptoms are: (a) transient early wheeze, which occurs before the age of 3 years and resolves by age of 6 years without lung function impairment; (b) late-onset wheeze, which develops after 3 years of age, persists in childhood, is linked to atopy and in some studies with reduced lung function and high bronchial hyperresponsiveness; (c) persistent wheeze, which starts in early life before 3 years of age and is associated with atopy, high immunoglobulin (Ig) E levels, early allergen sensitization and diminished lung function by school age [3,4,5]. However, these phenotypes can be described only retrospectively and are not helpful in guiding treatment. The European Respiratory Society (ERS) task force on preschool wheezing proposed a simpler clinical classification of wheezing as “episodic viral wheezing” (EVW) or “multiple trigger wheezing” (MTW) based on triggers and symptoms [6,7,8,9].

Despite many efforts, there is a large gap in knowledge regarding the management of preschool wheezing. In order to fill this lack of knowledge, the aim of these guidelines are to define the management of wheezing disorders in preschool children (aged up to 5 years).

## 2. Materials and Methods

We set up a multidisciplinary panel of experts that included all the Heads of the Pediatric Units of the Emilia-Romagna Region Italy, the Heads of the outpatient clinics for pulmonology and allergology, a sample of primary care pediatricians (identified in each province based on the size of the pediatric population according to ISTAT 2018 data) and a patients’ association (Respiro Libero, Parma, Italy). This study group (named Emilia-Romagna Asthma Study Group and described in detail in a previous publication on management of children with acute asthma attack [10]), included members with previous experience in the development of documents and recommendations with the Grading of Recommendations Assessment, Development, and Evaluation [11,12].

The aim was to address twelve different key questions regarding the management of preschool wheezing. Clinical questions have been formulated by the expert panel using the PICO format (Patients, Intervention, Comparison, Outcomes) and systematic reviews have been conducted on PubMed to answer these specific questions with the aim of formulating recommendations. Each subgroup (at least two people) formulated a search strategy and reviewed the retrieved references for relevant papers. Details are reported in Supplementary Material S1. Clinical questions have been grouped in three main topics:

(1) Definition of preschool wheezing

PICO question 1: Is the distinction between EVW and MTW useful for clinical practice?

(2) Management of the acute attack

PICO question 2: Are short-acting β2-agonists (SABA) useful in exacerbation of preschool wheezing?
PICO question 3: Are oral corticosteroids (OCS) useful in exacerbation of preschool wheezing?
PICO question 4: Are inhaled corticosteroids (ICS) useful in exacerbation of preschool wheezing?
PICO question 5: Are antibiotics useful in exacerbation of preschool wheezing?
PICO question 6: Is ipratropium bromide useful in exacerbation of preschool wheezing?
PICO question 7: Are leukotriene receptor antagonist (LTRA) useful in exacerbation of preschool wheezing?

(3) Controller therapy for the preschool child with wheezing

PICO question 8: When should controller medication be started?
PICO question 9: In children with preschool wheezing, are inhaled steroids more effective (and/or safer) than LTRA?
PICO question 10: How long should controller therapy with ICS be continued?
PICO question 11: Is intermittent therapy starting after symptoms onset with ICS preferred to daily therapy?
PICO question 12: How long should controller therapy with LTRA be continued?

The GRADE approach has been used for each selected paper to assess the confidence in the evidence (quality) and the degree of recommendations, as reported in Supplementary Material S2 [11]. Recommendations are graded as strong or weak after considering the quality of the evidence, the balance of desirable and undesirable consequences of compared management options, the assumptions about the relative importance of outcomes, the implications for resource use, and the acceptability and feasibility of implementation [12]. The panel has then decided on the strength of recommendations. A dedicated voting process (collection of voting forms through individual email messages) was developed for the present guidelines and an online meeting with participation of the full voting panel was organized. More specifically, voting panel members were provided with the results of the various literature searches, the evidence summaries, the proposed recommendations, and the related GRADE tables. Each voting member was then allowed to individually vote in favor or against each of the recommendations, to propose possible modifications, and to judge each recommendation as strong or weak according to GRADE rules. For recommendations with an agreement < 75%, further voting rounds were conducted after implementation of dedicated amendments based on the provided comments. After reaching an agreement ≥ 75% for all recommendations, all the authors reviewed and approved the final manuscript and Supplementary Material S1.

## 3. Results

### 3.1. Definition of Preschool Wheezing

#### 3.1.1. PICO Question 1: Is the Distinction between EVW and MTW Useful for Therapeutic Strategies?

##### Executive Summary

Two wheezing phenotypes have been described by the European task force on the basis of the trigger: EVW and MTW [13,14,15,16]. A wheezing typically exacerbated by a viral upper respiratory tract infection with few or no symptoms in the interval between the episodes was described as EVW and is the commonest phenotype between 1 and 5 years of age. These children are less atopic, respond to intermittent therapy at the time of symptoms and are less likely to develop asthma. Children who have symptoms that resemble asthma with wheezing also between respiratory infections and during activity, crying or laughing, show the phenotype traditionally called MTW [13,14,15,16]. Children with MTW are usually atopic, may have a family history of asthma, respond to regular ICS, develop an early and permanent reduction in lung function and go on to develop asthma at school age. Nevertheless, several studies documented that these phenotypes are unstable and can change over time [13,14,15,16].

A meta-analysis by Brodlie and colleagues reported that in EVW, maintenance and intermittent therapy with montelukast was not associated with significant benefits but only with a modest reduction in the severity of symptoms [17]. In contrast with these results, Yoshihara and colleagues found a significant reduction in asthma recurrences triggered by viral cold in preschoolers treated with montelukast as controller therapy [18]. No difference was found with the use of ICS instead [18].

A study by Ducharme and colleagues investigated the efficacy of as needed, high-dose ICS (750 mcg of fluticasone propionate), administered at the first sign of an upper respiratory tract infection in preschoolers with EVW [19]. This approach resulted in reducing the need for OCS and in milder symptoms, shorter episodes, lower use of SABA and higher quality of life [19]. A study by Bacharier and colleagues, analyzed the efficacy of as-needed ICS and montelukast in children with moderate to severe EVW [20]. The results of this study showed no difference in terms of episode-free days, OCS use, healthcare utilization, quality of life or hospitalization between the three groups (as needed ICS vs. as needed montelukast vs. placebo). Despite this, both montelukast and ICS were associated with a reduction in the severity of the episodes [20]. In line with these results, a study by Clavenna et al. found no difference in wheezing episodes between children with EVW receiving ICS vs. placebo during an upper respiratory tract infection [21]. Another meta-analysis reported no benefits with the administration of daily ICS for EVW, while the severity of the episodes was reduced by as needed ICS [22].

Regarding MTW, a study by Kahlon and colleagues reported that daily montelukast was effective in about two thirds of children in a cohort of preschoolers in terms of symptom control [23]. Interestingly, children who had developed symptoms earlier in life had the worst symptom control. Another study focusing on MTW reported that preschoolers receiving daily ICS during an acute wheezing episode had more symptom free days, milder symptoms and minor need for SABA (although the result for this outcome did not reach statistical significance) [24]. Pelkonen and colleagues analyzed a cohort of patients with both MTW (83%) and EVW (17%), aged 6 to 24 months [25]. Montelukast showed no efficacy in reducing symptom free days, use of rescue medication, or number of exacerbations. The young age of the patients included in the study and the lack of distinction between wheezing phenotypes, however, make these results hard to be generalized to a wider population [25].

Yoshihara and colleagues reported the effects of a combination therapy with ICS and bronchodilators in MTW, showing an improvement in terms of the number of exacerbations, symptom scores, weekly SABA use, and quality of life [26,27]. The meta-analysis by Kaiser et al. also reported that daily treatment with ICS is effective against MTW in reducing severe exacerbations [22]. When compared with montelukast, ICS achieve a better control of symptoms in these children. It must be noted that no results regarding as needed therapy in MTW were found. This may be due to the fact that usually these children are given a controller therapy because of the high frequency of their symptoms and the difficulty in anticipating the development of a new wheezing episode.

Considering the available studies overall, it was shown that phenotype-tailored therapy might be useful, but these phenotypes are usually unstable over time [13,14,15,28].

**Recommendation 1.** The distinction between EVW and MTW is useful for the therapeutic strategy, but should be periodically reassessed as it may change over time. The choice between montelukast and ICS, and between daily or intermittent therapy should take into account the wheezing phenotype (EVW or MTW), severity of symptoms and family history.

**Quality of evidence: Moderate. Strength of recommendation: B**

### 3.2. Management of the Acute Attack

#### 3.2.1. PICO Question 2: Is Inhaled SABA Useful in Exacerbation of Preschool Wheezing?

##### Executive Summary

The role of inhaled SABA in children with wheezing is well established in the literature. SABA represent the first-line treatment in preschool, school-aged children and adolescents with asthma-like symptoms, administered via a pressurized metered-dose inhaler (pMDI) with a spacer in mild-to-moderate attacks or via nebulization driven by oxygen in severe attacks, according to the major international guidelines [29,30,31].

The efficacy of inhaled SABA in children under 2 years of age during wheezing episodes is unclear, since in this age group this symptom often occurs in the setting of infectious bronchiolitis. For this reason, the British Thoracic Society (BTS) guideline on asthma management suggests considering an alternative diagnosis and other treatment options in children under two who have a poor initial response to SABA [29]. Similar indications are present in the Global Strategy for Asthma Management and Prevention document of the Global Initiative for Asthma (GINA), where a diagnosis of bronchiolitis must be considered in children with episodes of wheeze younger than 12 months [30].

A recent systematic review conducted by Pollock et al. aimed to present a comprehensive synthesis of evidence on the efficacy and safety of SABA to treat asthma and wheezing exacerbations in children aged 0–18 years presenting to the emergency department (ED) [32]. In their work, authors included 13 systematic reviews containing 56 relevant trials and 5526 patients demonstrating the efficacy of SABA delivered by pMDI as a first-line therapy for younger and older children (hospital admission decreased by 44% in younger children and ED length of stay decreased by 33 min in older children). Regarding children aged 0–3 years, this systematic review included a trial conducted on 28 patients in which SABA was compared to placebo: no effect on hospital admission (*p* = 0.51; low-quality evidence) was found. However, children treated with SABA showed a significant decrease in clinical severity score after 30 min (standardized mean difference (SMD): 1.31; 95% CI: 2.14 to 0.48), decrease in respiratory rate (mean difference (MD): 5.10; 95% CI: 9.45 to 0.75) and increase in oxygen saturation (MD: 1.60; 95% CI: 0.33–2.87) [32].

In children, in the case of pMDI use, the concomitant use of a spacer device is particularly important as it improves the absorption of SABA by approximately 20–30% even in the presence of poor technique [33]. The delivery of SABA by pMDI with spacer was superior to nebulization in a 2013 Cochrane comprising 1897 children and 729 adults in 39 clinical trials [34]. Although the hospitalization rate between the two methods of administration did not differ significantly, the mean length of stay of pediatric patients in the emergency room was shorter in those who received pMDI spacer treatment as well as a lower risk of developing tachycardia or tremors. According to the authors, these results were not applicable to subjects suffering from life-threatening asthma exacerbation as the latter were excluded from the studies included in the aforementioned meta-analysis [34].

A previous literature review, conducted by Castro-Rodriguez et al. in 2004 and including six randomized controlled trials (*n* = 491), aimed to compare the efficacy of SABA given by pMDI with a valved holding chamber (pMDI + VHC) or nebulizer in children under 5 years of age with acute exacerbations of wheezing or asthma in the emergency department setting [35]. Patients who received SABA by MDI + VHC showed a significant decrease in the admission rate compared with those by nebulizer (OR 0.42; 95% CI: 0.24–0.72; *p* = 0.002), with an even more significant decrease in children with moderate to severe exacerbations (OR 0.27; 95% CI: 0.13–0.54; *p* = 0.0003). Furthermore, the clinical score significantly improved in the group who received SABA by MDI + VHC in comparison to those who received nebulizer treatment (SMD, −0.44; 95% CI: −0.68 to −0.20; *p* = 0.0003) [35]. A further advantage of administration of SABA by pMDI is the lower risk of spreading viral respiratory infections compared to administration by nebulization [36].

Both the European Medicines Agency (EMA) and the Food and Drug Administration (FDA) highlighted the importance of using an inhaler device appropriate for age. The EMA recommended the routine use of a pMDI with an age-appropriate spacer with a facemask for administration of SABA under the age of 4. EMA, FDA and other regulatory agencies such as The Medicines and Healthcare Products Regulatory Agency (MHRA) consider the use of nebulized salbutamol under the age of 2 off-label since safety and effectiveness of nebulized salbutamol in children below 2 years of age have not been established [37,38,39]. The lack of studies and the uncertainty of clear indications in salbutamol use in this age group might have different explanations. Usually, the first wheezing episode in children < 2 years occurs during bronchiolitis, a disease unresponsive to SABA since it is caused by mucous obstruction and airway oedema of bronchioles rather than muscular constriction (bronchospasm) in the bronchi. Secondly, in this age group assessing the response to SABA with lung function tests is difficult and performed only in very specialized centers.

In 2014 the Italian Drug Regulatory Agency (AIFA) published a warning that restricted the use of nebulized salbutamol for children older than 2 years of age following several cases of adverse effects occurring in young children attributable to dosage error or wrong administration route [40]. Despite some measures previously adopted, such as the inclusion of an explanatory posology correlation table (milligrams-milliliters-number of drops) and a warning on the risk of overdose due to administration error, serious adverse reactions have been reported in children under 2 years of age, attributable to dosage error, incorrect administration route and also to drug exchange with the use of salbutamol (solution to nebulize). Adverse reactions, mainly tremors and tachycardia, were severe and required hospitalization [37,40]. All reported cases resulted in resolution of symptoms after discontinuation of treatment. For these reasons, a pMDI with spacer is preferred over nebulization.

**Recommendation 2.** Inhaled SABA represents the first-line treatment in preschool children with asthma-like symptoms. In the case of a mild-to-moderate wheezing attack, a pMDI with spacer is preferred over nebulization in children under 2 years of age. Nebulization driven by oxygen should be reserved for severe attacks.

**Quality of evidence: High. Strength of recommendation: A**

#### 3.2.2. PICO Question 3: Are OCS Useful in Exacerbation of Preschool Wheezing?

##### Executive Summary

The benefit of a short course of systemic corticosteroids in school-aged children and adolescents with asthma exacerbation is well established in the literature [10]. However, this therapeutic approach is widely debated in preschool children with asthma-like symptoms, since the evidence is unclear. In their randomized, double-blind, placebo-controlled trial, Panickar and colleagues compared a 5-day course of oral prednisolone (10 mg once a day for children 10 to 24 months of age and 20 mg once a day for older children) versus placebo in preschool children during a mild-to-moderate attack of wheezing associated with a viral infection [41]. No significant difference was found between the placebo group and the intervention group in terms of duration of hospitalization (primary outcome, 13.9 h vs. 11.0 h), Preschool Respiratory Assessment Measure (PRAM) score at 4, 12 and 24 h, albuterol use and 7-day symptom score. Authors concluded that in preschool children with mild-to-moderate wheezing associated with a viral infection, oral prednisolone was not superior to placebo [41].

A post-hoc and replication analysis of two multicenter, double-blind, randomized, placebo-controlled trials conducted by Beigelman et al. compared oral prednisolone vs. placebo in preschool children with severe intermittent wheezing during acute episodes of severe lower respiratory tract infections (LRTIs) [42]. Prednisolone once daily (2 mg/kg/day for 2 days, followed by 1 mg/kg/day for an additional 2 days) was not superior to placebo in terms of total symptom scores, cough, wheezing and trouble breathing scores, and interference with activity score. Authors concluded that prednisolone during clinically significant LRTIs did not reduce symptom severity [42].

The results of the prospective, randomized, double-blind, placebo-controlled trial conducted by Foster et al. suggested that there might be clinical benefits from treatment with systemic corticosteroids in preschool children who wheeze [43]. Children presenting to a pediatric emergency department with virus-associated wheeze were treated either with oral prednisolone once-daily at a dose of 1 mg/kg/day for 3 days, or with placebo. The median length of stay in the hospital was longer in the placebo group (540 min (IQR 124–971)) than in the prednisolone group (370 min (121–709)) [43]. Several authors focused their attention on the results of this study, some of them with strong criticism for the methodology applied (justification and process of the post-hoc secondary superiority analysis) and advised against this practice [44]. Furthermore, these results may have been biased by the fact that episodes of wheezing were generally severe and that children younger than 2 years were excluded from the trial. This could have resulted in a better response to steroids since the included subjects were more likely to have already a diagnosis of asthma [45].

Conversely, a recent randomized, double-blind placebo-controlled trial of Wallace et al. showed that administration of 3 days of oral prednisolone (2 mg/kg, maximum 40 mg) to preschool children with acute wheeze did not influence PRAM score at 24 h after intervention, requirement for hospital admission, length of inpatient stay, or salbutamol use in the first 48 h [46].

A systematic review with meta-analysis conducted by Castro-Rodriguez et al. assessing the role of OCS in preschoolers with acute wheezing episodes showed that compared to placebo, OCS did not result in a significant difference in hospital admission, additional course of systemic corticosteroids or unscheduled visits (RR: 0.73; 95% CI: 0.35–1.52) [47]. However, a stratified analysis of data suggested a potential benefit in terms of lower hospital admission rates and less need for additional courses of systemic steroids in children with more severe wheezing exacerbations presenting to the ED or requiring hospitalization [47].

**Recommendation 3.** A course of OCS is not routinely recommended in preschool children with acute wheezing attack, but it can be considered in the case of severe wheezing exacerbation that requires access to the emergency department, or requires hospitalization.

**Quality of evidence: High. Strength of recommendation: C**

#### 3.2.3. PICO Question 4: Are ICS Useful in Exacerbation of Preschool Wheezing?

##### Executive Summary

Administration of ICS at the onset of respiratory symptoms in young children with EVW is defined as “intermittent therapy”. This consists of the administration at home of high doses of ICS for 7–10 days in association with SABA, and is considered by the most recent guidelines and recommendations on management of preschool wheezing [30,48].

Since 2008, five randomized controlled trials compared SABA alone to intermittent ICS. The study by Zeiger et al. in children aged 12–53 months demonstrated that budesonide inhalation suspension (1 mg, twice daily for 7 days) at the first signs of respiratory tract infection was associated with a reduction in exacerbations requiring systemic corticosteroids [49]. On the other hand, the study by Bacharier et al. in children of the same age using the same pharmacologic approach did not show differences in rates of severe exacerbations requiring systemic steroids, but resulted in a modest reduction in trouble breathing, particularly in children with positive asthma predictive indices [50]. In the trial by Ducharme et al. in children aged 1–6 years, the therapeutic strategy consisted in intermittent fluticasone (750 mcg twice daily at onset of a respiratory tract infection for up to 10 days). The results showed a significant reduction in exacerbation rate in the group of children treated with fluticasone compared with placebo (8% vs. 18%, respectively) [19].

Papi and colleagues studied the efficacy of one-week treatment with nebulized beclomethasone dipropionate (400 mcg twice daily) plus salbutamol as needed, versus nebulized placebo plus salbutamol as needed in 166 preschool children with multiple trigger wheezing. The percentage of symptom-free days was significantly higher in the beclomethasone group (54.7%) than in the placebo group (40.5%) [24]. In a large study of more than 500 children with a history of EVW in the preceding 12 months, there was no significant difference in the rate of wheezing between those treated for 10 days with beclomethasone 400 mg twice a day and those receiving placebo (6.8% vs. 11.1%; *p* 1⁄4 0.09) [21]. A smaller gain in weight and height was reported in only one of the five trials [19].

Over the years three metanalyses summarized the evidence of intermittent therapy with high dose ICS [22,51,52]. The Cochrane systematic review reported that in preschool children with frequent wheezing episodes in comparison to placebo, the use of intermittent ICS at the onset of early symptoms reduced the likelihood of requiring rescue OCS by half, with an associated improvement in daytime and nighttime asthma symptoms’ score and parental perceived quality of life of children [51]. The meta-analysis by Kaiser et al. included data from six studies (*n* = 588) (three of which have been published before 2008) and confirmed a significant reduction in rates of severe exacerbations when using intermittent ICS compared with placebo (24.8% and 41.6%, respectively) [22]. The most recent systematic review included seven studies published between 1986 and 2014 (*n* = 1184 children) reporting on the use of budesonide (*n* = 4) and beclomethasone (*n* = 3). Conclusions stated that intermittent high-dose treatment of EVW with high dose ICS is as effective as daily treatment, while reducing overall corticosteroid exposure [52].

Since preschool children can experience several episodes of respiratory infections, the risk of an intermittent therapy approach is administering high doses of ICS very often, potentially affecting a child’s growth. A recent meta-analysis assessed the safety of high doses of ICS (for courses up to 14 days) in children. The study included 68 randomized trials for a total of 11,505 children and suggested that short-term high-dose ICS use is not associated with an increase in adverse events [53].

**Recommendation 4.** In preschool children with EVW but symptoms that are not persistent, intermittent therapy with high dose ICS could be used for 7–10 days at the first sign of respiratory infection.

**Quality of evidence: Moderate. Strength of recommendation: C**

#### 3.2.4. PICO Question 5: Are Antibiotics Useful in Exacerbation of Preschool Wheezing?

##### Executive Summary

Viral infections are considered the main trigger of wheezing exacerbations in preschool children [54]. However, some studies have demonstrated that bacterial infections can also have a relevant role. Stokholm et al. discovered that airway bacteria and respiratory viruses were equally associated with episodes of asthma-like symptoms in the first 3 years of life [55]. Esposito et al. showed the significant relationship of Mycoplasma pneumoniae and Chlamydia pneumoniae with wheezing in children, particularly in subjects with a history of recurrent episodes [56].

Interestingly, studies on bronchoalveolar lavage (BAL) of young children with recurrent wheeze, demonstrated the predominance of neutrophilic inflammation suggesting bacterial infection [57,58]. Schwerk et al. in a retrospective study compared the BAL of children with severe recurrent wheezing or persistent wheezing (*n* = 42) with a control group (*n* = 14) [57]. Of the 42 children with severe wheezing, 34 (81%) had neutrophilic inflammation and 20 of these a bacterial count (≥10^4^ CFU/mL) significantly higher than the control group (*p* < 0.005). H. influenzae, S. pneumoniae and M. catarrhalis were the most frequently isolated species. In addition, most of patients who had received antibiotic therapy (24 out of 26, 92%) with amoxicillin or amoxicillin and clavulanic or cefuroxime or trimethoprim and sulfamethoxazole for a median of six weeks (range 2–16 weeks) improved upon follow-up examination (showing fewer symptoms and exacerbations according to the international study of asthma and allergies in childhood (ISAAC) questionnaire). The rate of hospitalization due to severe wheeze dropped from 69% in the year before antibiotic therapy to 15% in the follow-up period (18 vs. 4 patients, *p* < 0.005) [56]. On the contrary, Patra et al. in an observational study involving children aged < 2 years of age and admitted with first time wheezing (*n* = 47) did not find a difference between children receiving antibiotics and those who did not [59].

Recently, three important randomized, controlled trials investigated the role of azithromycin in preschool wheezing [57,60,61]. Stokholm et al. recruited children aged 1–3 years with a history of recurrent asthma-like symptoms and randomized them to either azithromycin (3 days at 10 mg/kg/day) or placebo, at each episode of wheezing lasting at least 3 days. Therapy with azithromycin resulted in a shorter episode in 63.3% of cases (3.4 days versus 7.7 days, *p* < 0.0001) and this effect increased if the treatment was initiated within 6 days from the onset [55]. Another randomized, double-blind, placebo-controlled trial, conducted in 607 children aged 12–71 months with recurrent, severe LRTIs and minimal day-to-day wheeze impairment showed that azithromycin (12 mg/kg/day for 5 days) was associated with a significantly lower risk of progression to severe LRTI (HR 0.64, 95% CI 0.41–0.98, *p* = 0.04) [59]. In the third randomized, double-blind, placebo-controlled trial on azithromycin, no difference was found in the duration of the wheezing episode [61].

In a large retrospective Japanese study in children hospitalized for asthma exacerbation, in comparison with those who did not receive antibiotics, children with early antibiotic treatment had a longer hospital stay (mean difference 0.21 days, 95% CI 0.18–0.28), higher hospitalization cost (mean difference 83.5 USD, 95% CI 62.9–104) and greater probiotic use (RR 2.01, 95% CI 1.81–2.23) [62].

Overall, data did not confirm that antibiotics are useful to treat exacerbation of preschool wheezing. The mechanism of azithromycin in wheezing remains uncertain and the main concern with its use is the potential emergence of macrolide-resistant organisms and the negative influence on gut microbiota [63,64,65,66].

**Recommendation 5.** Antibiotics are not recommended in exacerbation of preschool wheezing.

**Quality of evidence: Moderate. Strength of recommendation: C**

#### 3.2.5. PICO Question 6: Is Ipratropium Bromide Useful in Exacerbation of Preschool Wheezing?

##### Executive Summary

Most studies included both young children and school-aged children with asthma. Wyatt and colleagues realized a prospective, single-blinded, randomized, controlled, equivalence trial in order to evaluate if the addition of ipratropium bromide by pMDI in moderate acute asthma in children affected hospital admission rates when compared with inhaled salbutamol and oral prednisolone alone [67]. Patients aged 2–15 years with acute, moderate asthma were randomized to two groups, one receiving salbutamol, prednisolone and ipratropium bromide, the other receiving only salbutamol and prednisolone. The managing doctor was blinded to the treatment. The admission rate in the ipratropium bromide group was 70.1% (122/174) compared with 64.2% (111/173) in the non-ipratropium bromide group. Adverse effects were more prevalent in the ipratropium bromide group. Authors concluded that the addition of ipratropium bromide in children with moderate acute asthma attack was not associated with reduction in admission rates [67].

A randomized controlled trial by Memon et al. compared the efficacy of three doses of nebulized salbutamol alone, or in combination with ipratropium bromide (250 mcg/dose) in children with acute severe asthma [68]. Efficacy was measured after 5 min from the last dose by change in severity score. The combination of nebulized salbutamol along with ipratropium bromide in the treatment of acute asthma exacerbation was not superior to salbutamol alone [68].

A systematic review from Vézina and colleagues tried to assess the efficacy and safety of anticholinergics added to SABA as inhaled or nebulized therapy in children hospitalized for an acute asthma exacerbation [69]. Authors included randomized trials comparing the combination of inhaled or nebulized anticholinergics and SABA versus SABA alone in children aged 1 to 18 years of age hospitalized for an acute asthma exacerbation. Primary outcomes were duration of hospital stay and serious adverse events. Secondary outcomes included admission and duration of stay in the intensive care unit (ICU), ventilation assistance, time to SABA spaced at four hours or longer, supplemental asthma therapy, duration of supplemental oxygen, change from baseline in asthma severity, relapse after discharge, adverse health effects and withdrawals. Seven randomized trials were included, four of which reported usable data on 472 children with asthma admitted to pediatric wards. No trials included patients admitted to the ICU. Results showed that the addition of anticholinergics to SABA had no effect on the duration of hospital admission (MD −0.28 h, 95% CI −5.07 to 4.52, three studies, 327 participants, moderate quality evidence) and no serious or non-serious adverse events were reported in any included trials. No statistically significant group difference was noted in other secondary outcomes, including the need for supplemental asthma therapy, time to SABA spaced at four hours or longer, asthma clinical scores, lung function and overall withdrawals for any reason [69].

Xu et al. realized a meta-analysis in order to evaluate the efficacy and safety of ipratropium bromide + salbutamol in the treatment of asthma in children and adolescents with asthma and in children < 1 year old with wheezing [70]. Studies were defined as eligible if they respected the following criteria: (1) randomized controlled trials; (2) patients < 18 years; (3) physician-diagnosed asthma and children less than 1 year old with wheezing; (4) comparing ipratropium bromide + salbutamol with salbutamol alone. Fifty-five studies were included with a total of 6396 participants. Results demonstrated that the association of ipratropium bromide and salbutamol significantly reduced the risk of hospital admission compared with salbutamol alone (risk ratio (RR) 0.79; 95% CI 0.66–0.95; *p* = 0.01; I2 = 40%). Subgroup analysis showed a significant difference in the risk of hospital admission in participants with severe asthma exacerbation (RR 0.73; 95% CI 0.60–0.88; *p* = 0.0009; I2 = 4%) and moderate-to-severe exacerbation (RR 0.69; 95% CI 0.50–0.96; *p* = 0.03; I2 = 3%), but not in those with wheezing in the first year of life. There were no significant differences in the risk of adverse events between ipratropium bromide + salbutamol group and salbutamol alone group (RR 1.77; 95% CI 0.63–4.98) [70].

**Recommendation 6.** Nebulization with ipratropium bromide is not recommended in exacerbation of preschool wheezing.

**Quality of evidence: Moderate. Strength of recommendation: C**

#### 3.2.6. PICO Question 7: Are LTRA Useful in Exacerbation of Preschool Wheezing?

##### Executive Summary

LTRA inhibit the activity of cysteinyl-leukotrienes, mediators of airway inflammation [71]. Montelukast is the only LTRA approved for preschool children and can be considered as a daily treatment to control wheezing symptoms. Five randomized controlled trials assessed the role of montelukast during the exacerbation of preschool wheezing [9,50,72,73,74].

Bacharier et al., compared in a randomized, double-blind and placebo-controlled trial the administration of a 7 day course of inhaled budesonide (1 mg twice daily) vs. montelukast (4 mg daily) vs. placebo in addition to albuterol in children with moderate to severe intermittent wheezing [50]. No difference was found over 12 months in the proportion of episode-free days, OCS use, health care access, quality of life and linear growth. However, both budesonide and montelukast seemed to reduce mean total symptoms score including trouble breathing and interference with activities [50].

A randomized, double-blind, non-inferiority trial evaluated the administration of montelukast in mild to moderate acute wheezing attacks as an alternative to OCS in children aged 2–17 years [72]. The group of children aged 2–5 years received the standard therapy in the emergency department (prednisolone 2 mg/kg + nebulized albuterol 250 mcg/dose + nebulized ipratropium bromide) followed by a course of OCS (1 mg/kg prednisone/prednisolone) or a course of montelukast (4 mg) for 5 days after discharge. Results showed that children on montelukast were more likely to have an exacerbation (22.4% vs. 7.9%, 95% CI 26.5–2.4%), especially patients aged < 3 years (OR, 4.9, 95% CI 1.66–15.22). Compared to patients treated with OCS, those receiving montelukast needed more additional medications such as additional bronchodilators, steroids, antibiotics and cough syrup (23.9% versus 9.5%, *p* = 0.03). Authors concluded that montelukast is not indicated for stabilization of patients with mild to moderate asthma attacks after hospital discharge [72].

Valovirta and colleagues compared, over a 1-year period, the efficacy of daily or episode-driven montelukast therapy (for 12 days) vs. placebo in a large multicenter randomized controlled trial including 1771 children. No difference was found among the three groups in acute attacks requiring physician or emergency room visit, administration of OCS or hospitalization [73].

A multicenter randomized controlled trial, involving 62 care sites in England and Scotland, enrolled 1358 children aged between 10 months and 5 years with at least two previous wheezing episodes to receive montelukast at each acute episode or placebo over a 12-month period [74]. Patients were stratified by ALOX5 promoter genotype and then assigned randomly to the two groups. In fact, depending on the copy numbers of the Sp1-bing motif in the ALOX5 gene promoter (that can be 5/5, 5/x or x/x, where x differs from 5), the response to montelukast in adults with asthma is known to be different. The trial found no difference in the number of unscheduled medical attendances for wheezing episodes between the two groups, or in the number or duration of wheezing episodes. Compared with placebo, unscheduled medical attendances for wheezing episodes were reduced in children with the 5/5 ALOX5 gene promoter given montelukast (*p* = 0.01). Authors concluded that there is no benefit of intermittent therapy with montelukast in preschool wheezing but patients with 5/5 ALOX5 promoter genotype might be responsive [74].

Demet Akbaş and colleagues conducted a randomized, double-blind, placebo-controlled, parallel-group clinical trial in 100 patients hospitalized for moderate-to-severe wheezing attacks [75]. Children were treated according to current guidelines with nebulized salbutamol, prednisolone and nebulized ipratropium bromide and, in addition, one group received montelukast (4 mg) and one group placebo. The trial showed no difference in length of hospital stay, clinical asthma score or oxygen saturation [75].

Three meta-analyses assessed the role of intermittent LTRA in preschool children with acute wheezing episodes [9,17,22]. Compared with placebo, the intermittent administration of montelukast did not reduce the number of wheezing episodes requiring OCS (RR 0.85, 95% CI 0.64 to 1.14, *n* = 343) or the visits to the emergency department (OR 1.00, 95% CI 0.49 to 2.02, *n* = 238) [17,73]. Moreover, no difference was found between treatment with intermittent ICS or intermittent montelukast (RR 0.82, 95% CI 0.59 to 1.15) in the acute phase of wheezing [22].

Several systematic reviews of the literature found the same results [76], even if few studies demonstrated a modest reduction in symptom severity and reduction in healthcare use in children presenting with positive asthma predictive indices [6,77,78,79,80,81].

**Recommendation 7.** LTRA are not recommended in exacerbation of preschool wheezing.

**Quality of evidence: High. Strength of recommendation: C**

### 3.3. Controller Therapy for the Preschool Child with Wheezing

#### 3.3.1. PICO Question 8: When Should Controller Medication Be Started?

##### Executive Summary

According to GINA recommendations, there are no validated tools to assess asthma control during preschool age, but the decision to start a controller daily therapy should be guided by parental reports (daytime and nighttime symptoms, need for rescue medication and level of activity impairment) and by the likelihood of future acute exacerbations. Whenever symptoms are not controlled, a continuative daily therapy with a low-dose ICS for at least 3 months is recommended [30]. BTS guidelines encouraged the use of ICS in preschool children with one or more awakenings at night/week, or with symptoms, or use of SABA ≥ three times/week [29], whereas NICE guidelines recommend starting ICS if symptoms (nocturnal awakenings, cough) occurred three times per week or more [82]. The recent Italian intersociety consensus published in 2021, summarizing all these guidelines and recommendations, concluded that a trial with daily ICS is recommended in all preschool children with recurrent wheezing or with less frequent but severe exacerbations, regardless of the definitive diagnosis [83].

Uncertainty remains about the dosage of daily ICS with both BTS and GINA documents suggesting a low daily dose of ICS eventually stepping up to a medium dose if symptoms do not improve (BTS/SIGN). Conversely, the NICE guidelines recommended starting with a moderate dose of ICS for 8 weeks, but this statement was based on expert opinion [82]. However, all recommended adjustment of the daily dose of ICS to the lowest dose required to control symptoms. A trial with daily ICS is important to exclude other diagnoses and should be restarted if symptoms relapse after interruption.

The evidence for these recommendations resulted from two European Respiratory Society Task Forces on preschool wheezing (2008 and 2014) stating that in children with MTW and in those with frequent or severe episodes of EVW, a 3 month trial with daily ICS treatment can improve symptoms, exacerbation rate and lung function [6,84]. Several studies and systematic reviews demonstrated the efficacy of ICS in children with preschool wheezing.

The study by Papi et al. in 2009, was a randomized, double-blind, double-dummy, placebo controlled clinical trial in preschool children (*n* = 276) with recurrent wheezing (history of at least three episodes of wheezing requiring medical attention in the previous six months and with wheezing symptoms at the time of enrolment), that showed the efficacy of ICS treatment over SABA as needed, in terms of symptoms and longer time to first exacerbation. Three treatments were assessed for a three-month period: (a) regular nebulized glucocorticoid plus SABA as needed versus (b) as needed nebulized ICS + SABA versus (c) placebo and as needed SABA. The study showed that the number of symptom free days was higher in the regular beclomethasone group compared to the as needed SABA group (*p* = 0.034, (95% CI: 0.58–13.15)) but not compared to the as needed ICS + SABA combination (*p* = 0.293, (95% CI: –2.38–7.62)). Compared to the salbutamol group, both beclomethasone and combination groups demonstrated lower symptom scores and longer time to first exacerbation, while no statistically significant differences were found among the three groups in regard to the use of relief medication [85].

Brand and colleagues conducted a randomized controlled trial to assess the role of ciclesonide in patients aged from 2 to 6 years with recurrent wheezing and positive asthma predictive index or atopy, without considering patients with EVW. Patients were observed for 2–4 weeks and if presenting with wheezing symptoms or need for rescue medication, they began a 24 week continuative therapy with ciclesonide at different dosages (40, 80 or 160 mcg) or placebo. Even if no differences between groups were found in time to first severe exacerbation requiring OCS, exacerbation rates were lower in pooled ciclesonide groups (6.2%) than in the placebo group (10.2%) (RR: 1.65 (95% CI: 1.04–2.63), (*p* = 0.030)). Compared to baseline, when patients were presenting ongoing symptoms, there was a significant improvement (*p* < 0.0001) in symptom scores and rescue medication use in all groups but no difference was found between ciclesonide groups and placebo. Lung function, in particular FEV1 and FEF 25–75, significantly improved in the ciclesonide group (*p* < 0.05) compared to placebo [86].

In 2009, a meta-analysis of 29 studies (*n* = 3592) showed that preschoolers with a clinical diagnosis of wheezing for at least 6 months before study enrolment who were treated for at least 4 weeks with ICS had significantly less exacerbations compared to placebo (18.0% vs. 32.1%, RR: 0.52 (0.43–0.63), *p* = 0.0001). The result was independent of age (infants or preschoolers), history of atopy and type or delivery mode of ICS (budesonide, beclomethasone and fluticasone, nebulized or metered dosed inhaled). Moreover, compared to the placebo group, patients treated with ICS presented lower withdrawal rates due to exacerbations, reduced SABA use and improved lung function measured as PEF and FEV1 [87]. The same author recommended daily ICS in preschoolers with recurrent wheezing in the previous 6 months, especially in those with risk factors for asthma [88]. 

In a recent review, continuative treatment with daily ICS was suggested in the case of: persistent symptoms (meaning 2 days/week or 2 nights/month), patients at higher risk for exacerbations (parental asthma, atopy, blood eosinophilia or multiple-trigger wheezing) with four or less episodes of wheezing in the previous year, patients with at least two exacerbations treated with OCS in the previous 6 months [89].

The most recent meta-analysis about this topic dates to 2016 and included 15 randomized controlled trials (*n* = 3278) comparing daily ICS to placebo and to montelukast. The analysis showed a reduced exacerbation rate with a daily medium-dose of ICS compared to placebo (RR: 0.70; 95%CI: 0.61–0.79; NNT = 9), especially in those with persistent asthma (RR 0.56; 95% CI, 0.46–0.70; NNT = 11). Moreover, daily ICS reduced exacerbation rate even if compared with montelukast (RR 0.59; 95% CI, 0.38–0.92) [22].

**Recommendation 8.** In preschool children with persistent or recurrent wheezing and in those with severe exacerbations, daily controller therapy with daily ICS should be started.

**Quality of evidence: High. Strength of recommendation: B**

#### 3.3.2. PICO Question 9: In Children with Preschool Wheezing, Are ICS More Effective (and/or Safer) Than LTRI?

##### Executive Summary

The use of LTRAs is an alternative to ICS for the treatment of mild and moderate persistent asthma and preschool wheezing in children [71].

Most comparative studies (randomized controlled trials, observational studies, systematic reviews and meta-analyses) suggested that ICS therapy is generally more effective than montelukast in children [6,22,90,91,92,93,94,95,96,97,98]. In one of the biggest meta-analyses, Kaiser et al. demonstrated that in preschool children with asthma or recurrent wheezing, exacerbations were reduced more by daily ICS than by daily montelukast [22]. In a more recent systematic review, Castro-Rodriguez et al. confirmed these results: compared to montelukast, ICS were associated with better control of symptoms and less exacerbations and less need for rescue systemic corticosteroids in preschool children with wheezing, but the level of evidence was weak [98]. Brand et al. suggested that maintenance treatment with ICS is recommended for MTW and that montelukast can be prescribed for the treatment of EVW and started at the onset of symptoms of a viral cold [6]. Given the large overlap in phenotypes and the fact that patients can move from one phenotype to another, a trial with ICS and montelukast may be considered in almost any preschool child with recurrent wheezing.

Some studies showed a similar effectiveness of LTRA and ICS [99,100,101,102,103,104,105,106], whereas other research reported better outcomes for montelukast [103,107,108]. Wu et al. observed that subjects with allergic rhinitis treated with LTRA were less likely to experience emergency department visits compared with subjects treated with ICS [103]. In a Chinese retrospective cross-sectional observational study with questionnaire-based analysis from 2021, Chen et al. found a lower percentage of asthma symptoms, night coughing, reliever medication, decreased asthma control and better exercise tolerance in the LTRA group [108].

Adherence to therapy is a major issue in wheezing and asthma. In 2012 Elkout et al. showed that medication possession ratio (index of adherence) was poor in all patients regardless of the maintenance therapy (ICS, LABA, LTRA), with only 15–39% having adequate adherence over a 1 year period [100]. However, treatment with LTRA had a higher medication possession ratio, while undersupply was greater in the ICS group. In younger children, oral montelukast may be easier to take with once-a-day administration while treatment with ICS might be associated with poor compliance [90,100,101,105,107]. Both ICS and montelukast have minimum adverse drug reactions [90,91,105]. However, most of the studies included a wide age range and some of the systematic reviews and meta-analyses also included adults.

**Recommendation 9.** ICS are recommended as a first choice as controller therapy in preschool children with wheezing, but montelukast could be considered in case of a lack of cooperation or poor compliance.

**Quality of evidence: Moderate. Strength of recommendation: B**

#### 3.3.3. PICO Question 10: How Long Should Controller Therapy with ICS Be Continued?

##### Executive Summary

The study by Bacharier and colleagues compared the efficacy of daily ICS versus placebo over a 2-year period in 285 atopic children with wheezing [20]. The group treated with fluticasone showed more episode free days, less systemic corticosteroid courses and less need of urgent medical visits. Nevertheless, these beneficial effects disappeared in the following year after treatment interruption.

Another study published in 2009 by Papi et al. found that controller therapy with daily ICS for 3 months was effective in increasing symptom free days and reducing daytime symptom score, night-time symptom score and number of nocturnal awakenings [85]. In line with these results, Kooi et al. reported a significant reduction in airways resistance and symptoms score after 3 months of treatment with daily ICS [99].

A study by Brand and colleagues found that controller therapy with daily ICS reduced the risk of acute exacerbation over a 24-week period [86]. A recent Italian consensus on airway disease in children suggested that, when started, controller therapy should be continued for at least 3 months [83]. The same article reported that NICE 2017 guidelines suggested at least an eight-week trial with a moderate dose of ICS. According to this consensus, the efficacy of treatment should be assessed 4–8 weeks after initiation [83].

An interesting study by Kwong and colleagues assessed the level of asthma control in pre-school children after stepping down or suspending the controller therapy after 3 months of regular treatment and demonstrated that suspending the daily treatment was associated with a higher risk of poor control [109].

Overall, these results suggest that discontinuation of therapy should be based on symptoms control rather than on the duration of therapy.

**Recommendation 10.** Although there is no clear evidence regarding the ideal duration of treatment, in children with recurrent wheezing, controller therapy with ICS should be continued for at least 3 months. In case of good symptom control, the clinician can make an attempt to suspend the daily treatment and then reassess the child in the short-term.

**Quality of evidence: Low. Strength of recommendation: B**

#### 3.3.4. PICO Question 11: Is Intermittent Therapy Starting after Symptoms Onset with ICS Preferred to Daily Therapy?

##### Executive Summary

Only two studies directly compared daily ICS with intermittent ICS in the therapeutic management of preschool wheezing. The study conducted by Papi et al. was a multicenter, randomized, parallel-group, three-group, double-blind, placebo-controlled study in 276 preschool children. Subjects were given different treatment regimens for 12 weeks: one group (*n* = 110) received a daily dose of 800 mcg (400 mcg twice daily) of beclomethasone plus as needed SABA during the acute respiratory event, one group (*n* = 110) was treated with daily placebo plus high doses of beclomethasone 800 mcg and SABA as needed only during the acute respiratory event, and one group (*n* = 56) was given daily placebo and as needed SABA. The percentage of symptom-free days over 12 weeks was higher in the group treated regularly with beclomethasone compared to the group treated with salbutamol as needed, but similar to the group treated with the combination of beclomethasone/salbutamol. Both groups treated with daily beclomethasone and the group treated with the beclomethasone/salbutamol combination had the same improvement in day and night symptoms and nocturnal awakenings [85]. In the study by Zeiger et al. the effect of daily budesonide (0.5 mg once a day, increasing to 0.5 mg twice a day for 7 days during respiratory infection) was compared with intermittent treatment (budesonide 1 mg twice a day for 7 days). After 52 weeks, there was no difference between the groups in the frequency of exacerbations requiring rescue therapy with OCS and there was a similar number of wheezing episodes and number of days absent from school/day care [49].

A 2013 Cochrane meta-analysis comparing intermittent and daily ICS found no significant differences between the two treatments (RR 1.09; 95% CI, 0.85–1.41; *p* = 0.49) [110]. In agreement with these data, Rodrigo et al. produced a meta-analysis including seven placebo-controlled trials (*n* = 1367) with a minimum of 8 weeks of daily ICS with rescue SABA versus intermittent ICS plus SABA at the onset of symptoms [111]. No statistically significant difference in the rate of asthma exacerbations between those with daily vs. intermittent ICS was noted (0.96; 95% CI: 0.86, 1.06, I2 Z 0%). However, compared to intermittent ICS, the daily ICS group had a significant increase in asthma-free days.

In two non-systematic reviews on preschool wheeze treatments, daily and intermittent ICS showed the same efficacy in reducing severe exacerbations requiring OCS [88,112]. Similarly, a 2015 Cochrane review did not find significant difference in hospital admissions or in the quality of life between the two regimens [51]. The meta-analysis conducted by Kaiser analyzed, as a subgroup, the two studies (*n* = 498) by Papi and by Zeiger and found no difference in symptom free days between the two groups. Furthermore, both groups showed a protective role in the prevention of severe exacerbations (daily ICS reduced the risk by 30%, intermittent ICS reduced risk by 36% and there were no significant differences when these strategies were compared directly). Additionally, no difference was found in severe exacerbations between daily and intermittent ICS (daily ICS 25.7%, intermittent ICS 28.1%; RR 0.91; 95% CI, 0.71–1.18; *p* = 0.49, I2 = 43%) [22]. In the most recent meta-analysis by Murphy, only the study by Zeiger was included [49]. Although this study was not adequately powered to demonstrate equivalence, authors concluded that intermittent and daily therapy showed the same efficacy in reducing exacerbations requiring OCS [52].

However, the NICE 2017 guidelines stated that proofs were insufficient to confirm that the intermittent use of ICS was better, worse, or equivalent to daily administration of ICS in children with preschool wheezing [82]. On the other hand, GINA 2022 reported that in subjects at Step 1 with intermittent wheezing, in whom SABA was unable to control symptoms (particularly if they are atopic), ICS intermittent therapy can be considered. However, for children in step 2 regular administration of daily low dose ICS plus SABA as needed is recommended as the first choice treatment [113].

**Recommendation 11.** In preschool children with recurrent or persistent wheezing, treatment with intermittent high dose ICS for 7–10 days at first signs of respiratory infection or daily ICS as controller therapy are both recommended to reduce the risk of wheezing exacerbations. A follow-up of the patient after 3 months is recommended to re-assess the clinical picture and the therapy.

**Quality of evidence: High. Strength of recommendation: B**

#### 3.3.5. PICO Question 12: In Case of Use of LTRA as Controller Therapy, How Long Should It Be Continued?

##### Executive Summary

Beneficial effects of montelukast have been reported within 6 weeks of treatment [114]. In a review, Jarrti et al. reported the results of some studies that fell out of our search string as they had been published before 2008 [92]. Amongst these, a study by Knorr and colleagues found that daily treatment with montelukast for 3 months was superior to placebo in the prevention of symptoms, SABA and OCS use [115]. Another study included in this meta-analysis found that a 6 weeks course with daily montelukast was not superior to placebo in any of the outcomes analyzed in children between 6 and 24 months old [116]. These contrasting results suggested that age may play a role in the response to montelukast [92].

The systematic review by Castro-Rodriguez and colleagues, studied the efficacy of montelukast in wheezers and children with asthma in comparison to ICS [98]. Among the studies included in this metanalysis, Krawiec et al. did not find any difference in symptom control between preschoolers treated with ICS, montelukast or placebo [104]. Jehan and colleagues [117], reported that 16.7% of the patients in their cohort could step down therapy after 6 months on montelukast (compared to 51.6% of those treated with ICS) [117]. Kooi and colleagues reported that montelukast reduced symptoms within 3 months from the beginning of therapy [99]. A prospective study by Kahlon and colleagues assessed the clinical response to montelukast in a cohort of preschoolers with MTW [23]. Nearly 70% achieved good control within 1 month of daily treatment, and almost all reported good symptom control after 3 months of treatment. In children with persistent wheezing, ICS given as maintenance therapy was more effective in controlling symptoms than montelukast [90]. Interestingly, the effects of montelukast were similar after 3 or 12 months.

A consensus by Brand and colleagues suggested that any controller therapy should be continued in at least a 2–3 months long trial to correctly assess the response of the patient [84]. In a study on infants treated with montelukast or ICS, both demonstrated a significant decrease in doctor visits, hospital admissions, use of bronchodilators and use of OCS after 3 months, and after 12 months of treatment [106]. An interesting study by Moeller and colleagues, found that montelukast reduced symptoms, bronchial hyperreactivity, fractional exhaled nitric oxide (FeNO) levels and increased lung function in preschoolers with high FeNO levels within just 8 weeks [118]. Shah and colleagues reported that controller therapy with montelukast was effective in reducing symptoms and increasing lung function within the first two weeks of treatment [119]. Wu and colleagues studied the effects of montelukast in young children with wheezing and in children with asthma at 4, 8 and 12 weeks [120]. In preschoolers, montelukast was effective in reducing symptom severity and the need for medical examination or rescue therapy. No difference in these parameters was demonstrated at the different time points of follow-up, suggesting that response to montelukast was already evident after 4 weeks of treatment [120].

**Recommendation 12.** Clinical effects of montelukast can be evident within a few weeks, but in case of its use a 3-month trial is suggested. If the child shows no response to this treatment, montelukast should be discontinued.

**Quality of evidence: Low. Strength of recommendation: B**

## 4. Discussion

This study shows that there are ongoing controversies in the management of preschool wheezing, mainly related to disease severity classification, ICS or montelukast responders and non-responders, long-term or intermittent therapy with ICS, and strategies able to alter the evolution of the disease into asthma.

In preschool children with wheezing, some limitations are mainly due to the variable disease profile and difficulties in performing lung function tests in the first years of life. Classification based on duration of wheeze (i.e., transient, persistent and late onset wheeze) is clinically useless, and can be assigned only retrospectively at school-age. Classification of wheezing based on patients’ temporal symptoms (i.e., EVW or MTW) can be used to guide management, but has certain limitations. In young children these two patterns are not sufficiently stable over time and can vary with treatment. More research should focus on disease severity in addition to phenotypes and endotypes.

ICS are the preferred long-term controller therapies in children regardless of age, to improve symptom control and quality of life. Certain groups of children with wheezing seem to respond better to ICS, especially those sensitized to aeroallergens and with high peripheral eosinophilia [91]. While effective for reducing impairment and exacerbations, daily maintenance therapy is not disease modifying and does not protect from the development of asthma or the decline in lung function [121,122]. Montelukast is considered an alternative to ICS when starting controller therapy. More studies are needed to better characterize ICS or montelukast responders and non-responders to allow for development of additional treatment options. However, it is unclear which children should be started with maintenance therapy (ICS or montelukast) to prevent symptoms and acute episodes. Family history of asthma, blood eosinophils and aeroallergen sensitization can help, but the decision is mainly based on parental reports of symptom patterns. Intermittent therapy with ICS at the first signs of viral respiratory illness can be an effective alternative to daily ICS treatment, particularly in children with EVW.

This consensus document aimed to respond to issues that are still poorly addressed, with the ambition of filling current shortcomings. Through the GRADE method, in our study the participants discussed the recommendations, and agreement was reached after an active discussion in some cases. It should be noted that the participants in the project came from different clinical contexts, i.e., they were pediatricians working in the hospital, in outpatient clinics for pulmonology and allergology and in primary care. The findings obtained can establish the basis for educational interventions that aim to optimize clinical management of preschool wheezing. The specific statements developed are intended to guide the healthcare professional in practice to ensure a better and standardized approach in children < 5 years of age. Table 1 summarizes the 12 recommendations. An extended evidence summary is available in the Supplementary Material.

## 5. Conclusions

These guidelines provide clear and shared indications for management of preschool wheezing, based on the most updated literature. This work represents, in our opinion, the most complete and up-to-date collection of recommendations to guide pediatricians in the management of the patient, thereby standardizing approaches. Our recommendations answer the need for a clear direction in clinical practice, although there is the awareness of the acquisition of new knowledge in the future. Undoubtedly, more research is needed to find objective biomarkers and understand underlying mechanisms to assess phenotype and endotype and to personalize targeted treatment. However, research in young children is difficult due to ethical issues and difficulties in obtaining lower airway samples and measuring lung function. Ideally, biomarkers should be easy to assess for general pediatricians, in order to choose the appropriate treatment. New therapeutic options such as allergen immunotherapy and monoclonal antibodies targeting Th2 inflammation need efficacy and safety studies in this age group. Finally, new strategies able to alter the evolution of the disease into asthma are required. 

## Figures and Tables

**Table 1:** Recommendations for clinical management of preschool wheezing.

PICO Question	Recommendation	Quality of Evidence	Strength of Recommendation
PICO question 1: Is the distinction between EVW and MTW useful for the clinical practice?	The distinction between EVW and MTW is useful for the therapeutic strategy, but should be periodically reassessed as it may change over time. The choice between montelukast and ICS and between daily or intermittent therapy should take into account the wheezing phenotype (EVW or MTW), severity of symptoms and family history.	**Moderate**	**B**
PICO question 2: Are SABA useful in exacerbation of preschool wheezing?	Inhaled SABA represent the first-line treatment in preschool children with asthma-like symptoms. In the case of a mild-to-moderate wheezing attack, a pMDI with spacer is preferred over nebulization in children under 2 years of age. Nebulization driven by oxygen should be reserved for severe attacks.	**High**	**A**
PICO question 3: Are OCS useful in exacerbation of preschool wheezing?	A course of OCS is not routinely recommended in preschool children with an acute wheezing attack, but it can be considered in the case of severe wheezing exacerbation that requires access to the emergency department or requires hospitalization.	**High**	**C**
PICO question 4: Are inhaled steroids useful in exacerbation of preschool wheezing?	In preschool children with EVW but symptoms that are not persistent, intermittent therapy with high dose ICS could be used for 7–10 days at the first sign of respiratory infection.	**Moderate**	**C**
PICO question 5: Are antibiotics useful in exacerbation of preschool wheezing?	Antibiotics are not recommended in exacerbation of preschool wheezing.	**Moderate**	**C**
PICO question 6: Is ipratropium bromide useful in exacerbation of preschool wheezing?	Nebulization with ipratropium bromide is not recommended in exacerbation of preschool wheezing.	**Moderate**	**C**
PICO question 7: Are LTRA useful in exacerbation of preschool wheezing?	LTRA are not recommended in exacerbation of preschool wheezing.	**High**	**C**
PICO question 8: When should controller medication be started?	In preschool children with persistent or recurrent wheezing and in those with severe exacerbations, controller therapy with daily ICS should be started.	**High**	**B**
PICO question 9: In children with preschool wheezing, are ICS more effective (and/or safer) than LTRA?	ICS are recommended as a first choice as controller therapy in preschool children with wheezing, but montelukast could be considered in the case of a lack of cooperation or poor compliance.	**Moderate**	**B**
PICO question 10: How long should controller therapy with ICS be continued?	Although there is no clear evidence about the ideal duration of treatment, in children with recurrent wheezing, controller therapy with ICS should be continued for at least 3 months. In case of good symptom control, the clinician can make an attempt to suspend the daily treatment and then reassess the child in the short-term.	**Low**	**B**
PICO question 11: Is intermittent therapy starting after symptom onset with ICS preferred to daily therapy?	In preschool children with recurrent or persistent wheezing, treatment with intermittent high dose ICS for 7–10 days at first signs of respiratory infection or daily ICS as controller therapy are both recommended to reduce the risk of wheezing exacerbations. A follow-up of the patient after 3 months is recommended to reassess the clinical picture and the therapy.	**High**	**B**
PICO question 12: How long should controller therapy with LTRA be continued?	Clinical effects of montelukast can be evident within a few weeks, but in case of its use, a 3-month trial is suggested. If the child shows no response to this treatment, montelukast should be discontinued.	**Low**	**B**

EVW, episodic viral wheezing; inhaled corticosteroids; LTRA, leukotriene receptor antagonist; MTW, multiple trigger wheezing; OCS, oral corticosteroids; pMDI, pressurized metered-dose inhaler; SABA, short-acting β2-agonists. Details on quality of evidence and strength of recommendations are reported in Supplementary Material S2.

## Data Availability

Not applicable.

## Supplementary Materials

The following supporting information can be downloaded at: https://www.mdpi.com/article/10.3390/jcm11164763/s1, Supplementary Material S1: PICO questions and literature analysis [6,9,17,19,20,21,22,23,24,25,26,27,32,41,42,43,46,47,49,50,51,52,55,57,59,60,61,62,67,68,69,70,72,73,74,75,85,86,87,90,91,92,93,94,95,96,97,98,99,100,101,102,103,104,105,106,107,108,110,111,117,118,120,123,124,125,126]. Supplementary Material S2: GRADE method witb details on quality of evidence and strength of recommendations.
