# Supplementary material for: Management of Preschool Wheezing: Guideline from the Emilia-Romagna Asthma (ERA) Study Group
Source: J Clin Med. 2022 Aug 15;11(16):4763. doi: 10.3390/jcm11164763 (PMC9409690; doi:10.3390/jcm11164763)
Supplement: Supplementary file 1 [file jcm-11-04763-s001.zip › jcm-1841734-supplementary.pdf]

Supplementary material S1: PICO questions and review of the literature

Clinical management of preschool wheezing: guidelines from the Emilia-Romagna Asthma (ERA) Study Group

Results of the clinical questions

1) Definition of preschool wheezing

PICO question 1. Is the distinction between episodic viral wheezing and multi-trigger wheezing useful for the therapeutical strategy?

Patient or population: children with preschool wheezing
Setting: primary care/emergency department
Intervention: phenotype definition
Comparison: viral and multi-trigger wheeze

Search strategy
((child, preschool[MeSH Terms]) OR (toddler*)) AND ((Respiratory Sounds[Mesh]) OR (wheez*) OR ("Asthma"[Mesh:NoExp])) AND ((Anti-Asthmatic Agents[Mesh]) OR (Adrenergic beta-2 Receptor Agonists[Mesh]) OR ("Bronchodilator Agents"[Mesh]) OR (albuterol[MeSH Terms]) OR (salbutamol) OR ((Anti-Asthmatic Agents[Mesh]) OR (Adrenergic beta-2 Receptor Agonists[Mesh]) OR ("Bronchodilator Agents"[Mesh]) OR (salmeterol)) OR ((("Administration, Inhalation"[Mesh]) OR (Inhaled)) AND ((ICS) OR (Adrenal Cortex Hormones[Mesh]) OR (*steroid*))) OR (((Administration, Oral[Mesh]) OR (oral) OR (systemic)) AND ((Adrenal Cortex Hormones[Mesh]) OR (*steroid*) OR (betamethasone) OR (prednisone) OR (dexamethasone))) OR (("Leukotriene Antagonists"[Mesh]) OR (montelukast)))

PICO n° 1 Workflow of study selection process

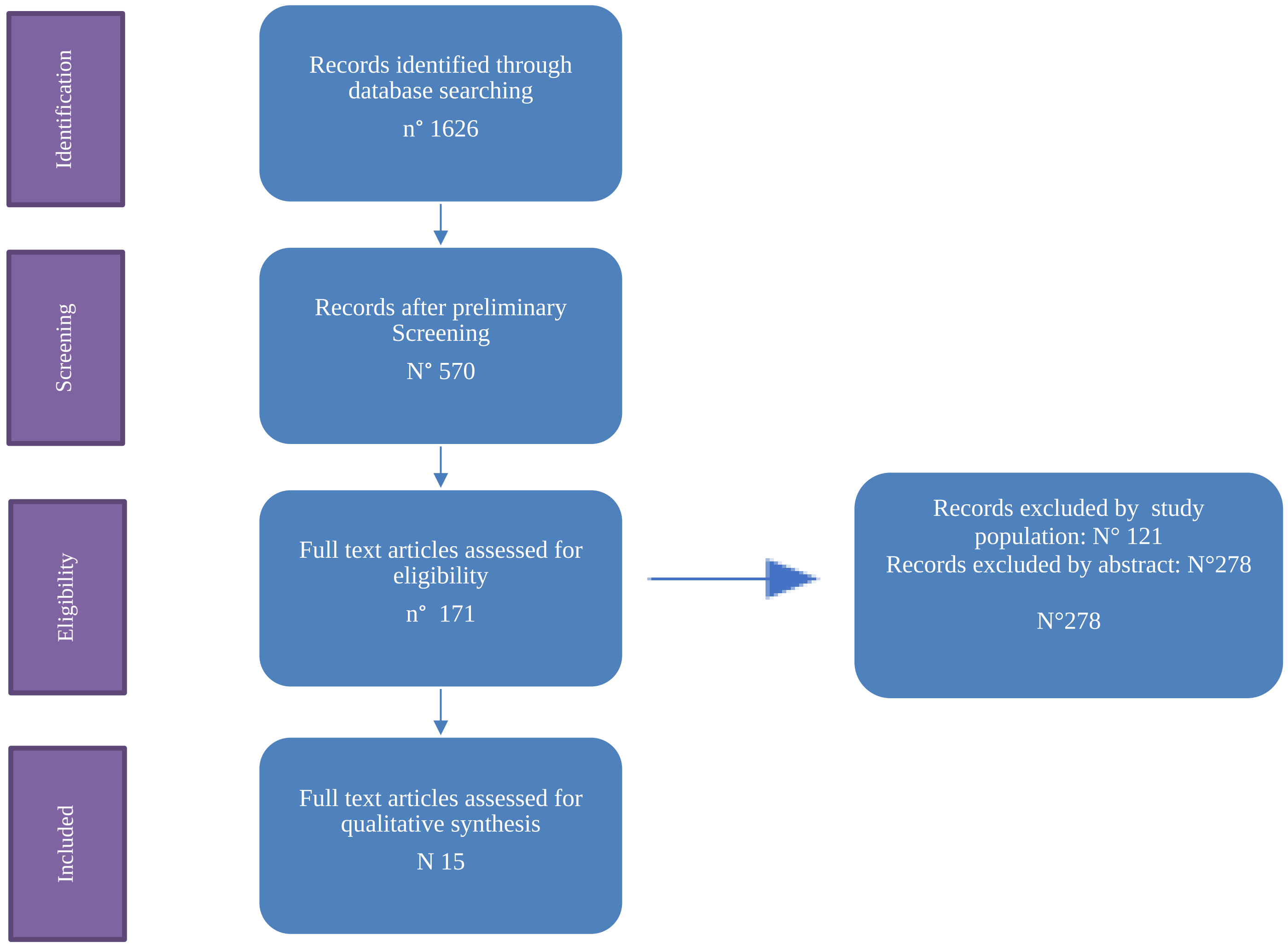

Modified from: Moher D, Liberati A, Tetzlaff J, Altman DG, The PRISMA Group (2009).
Preferred Reporting Items for Systematic Reviews and Meta-Analyses: The PRISMA Statement. PLoS Med 6(7): e1000097

**Title of the study, first author, year [ref]**	**Type of study**	**Study design**	**Population**	**N of patients (age)**	**Experiments/ mechanisms assessed**	**Outcomes (primary and secondary)**	**Effect [Relative risk (RR) or odds ratio (OR) + confidence interval (CI 95%)]**
Episodic use of an inhaled corticosteroid or leukotriene receptor antagonist in preschool children with moderate-to-severe intermittent wheezing. **Bacharier, 2008 [1]**	Randomized, double-blind placebo-controlled trial	As-needed ICS (budesonide 1.0 mg twice daily, n=96) vs as-needed LTRA (montelukast 4 mg once daily, n=94) vs as-needed placebo (n=47), to be started at first sign of respiratory tract infection/wheezing	EV Wheezers	237 (1-5 years)	Outcomes:<br>Episode-free days,oral corticosteroids use, healthcare utilization, quality of life, hospitalization rate<br><br>Symptoms score (cough + wheezing + trouble breathing + interference with activity)	No statistically significant difference<br><br>Total symptom score was lower in ICS and LTRA groups compared to placebo (24.6% reduction, p=0.02 in ICS group) (29.6% reduction, p=0.006 in LTRA group)[1]	
Preemptive use of high-dose fluticasone for virus-induced wheezing in young children. **Ducharme, 2009 [2]**	Parallel-group, randomized, placebo-controlled trial with triple blinding	As-needed ICS (fluticasone propionate 750 mcg twice daily, n=62) vs placebo (n=67)	EV Wheezers	129 (1-6 years)	Outcomes:<br>Oral corticosteroids use<br><br>Symptoms control<br><br>Rescue β2-agonist use	8 vs 18% URTIs[2] requiring systemic corticosteroids<br><br>5.66±4.37 vs 6.88±5.1 days with asthma-like symptoms during URTIs<br><br>4 (2 to 7) vs 6 (3 to 9) days of rescue β2-agonist use during URTIs	OR (ICS vs placebo) 0.49, CI 95% 0.30 to 0.83<br><br>Rate Ratio (ICS vs placebo) 0.82 CI 95% 0.71 to 0.95<br><br>Rate Ratio (ICS vs placebo) 0.80, CI 95% 0.68 to 0.94
Ciclesonide in wheezy preschool children with a positive asthma predictive index or atopy. **Brand, 2011 [3]**	RCT	Daily ICS (ciclesonide 40/80/160 mcg, n=224, 215 and 229 respectively) vs placebo (n=206) (24 weeks)	MT Wheezers with positive API[4] or screening for atopy	874 (2-6 years)	Outcomes:<br>Risk of acute exacerbation	10.2% vs 6.2% risk of severe exacerbation	RR 1.65 (placebo vs ciclesonide[5]), CI 95% 1.04-2.63
Short term efficacy of nebulized beclomethasone in mild-to-moderate wheezing episodes in pre-school children. **Papi, 2011 [4]**	Randomized, multicenter, double blind, parallel-group, placebo-controlled trial	Daily ICS (beclomethasone dipropionate 400 mcg twice daily, n=110) vs placebo (n=56) (both + as-needed salbutamol)	MT Wheezers during an acute episode (7 days of treatment)	166 (1-4 yo)	Outcomes:<br>Symptoms control	No asthma symptoms during the 1st week of treatment<br><br>Adjusted mean coughing score: -0.29 (95% CI of difference: -0.568, -0.002) using ICS	OR 2.65 (ICS vs placebo); 95% CI 95% 1.08-6.51
Intermittent or daily montelukast versus placebo for episodic asthma in children.	Multicenter, randomized, double-blind, double-	Daily LTRA (montelukast, 4-5 mg once daily) + as-needed placebo (n=584) vs	EVWheezers	1757 (6-5 yo)	As needed or controller therapy.		

**Valovirta, 2011 [5]**	dummy, parallel group trial	daily placebo + as-needed LTRA vs daily placebo + as-needed placebo (n=588)			Outcomes: Healthcare resource utilization, number of episodes, symptom-free days<br><br>Symptoms during acute exacerbation<br><br>b2-agonists use	No statistically significant difference in asthma episodes needing healthcare resource utilization, number of episodes, symptom-free days<br><br>Reduction of symptoms during an asthma-like episode in the daily LTRA group compared to placebo (the intermittent LTRA group showed a reduction that did not reach statistical significance)<br><br>Reduction in the use of b2-agonists, both in the daily LTRA group (-0.29, 95% CI -0.57, 0.00)<br>and<br>the intermittent LTRA group (-0.31 95% CI -0.59, -0.03), compared to placebo	Rate Reduction -0.12 95% CI -0.24/0.00
Daily or Intermittent Budesonide in Preschool Children with Recurrent Wheezing.<br>**Zeiger, 2011 [6]**	RCT	Daily ICS (budesonide 0.5 mg once daily, n=100) + as-needed placebo vs daily placebo + as-needed ICS (budesonide 1 mg twice daily, n=113)	MTWheezers	213 (12-53 mo)	As needed or controller therapy.<br><br>Outcomes: frequency of exacerbations requiring oral corticosteroids<br><br>time to first and second exacerbation<br><br>rate of treatment failure<br><br>episode-free days<br><br>symptom severity<br><br>absences related to respiratory symptoms<br><br>albuterol use<br><br>quality of life<br><br>FeNO	No significant difference between groups (p=0.60)<br><br>No significant difference between groups (p=0.87 and p=0.38, respectively)<br><br>No significant difference between groups (p=0.12)<br><br>No significant difference between groups (p not calculated)<br><br>No significant difference between groups in symptoms scores during respiratory tract illnesses or during exacerbations.<br><br>No significant difference between groups (both for parents and children; p not calculated)<br><br>No significant difference between groups (p not calculated)<br><br>No significant difference between groups (p not calculated)<br><br>Changes in values for FeNO were not reported	
The effect of montelukast on respiratory symptoms and lung function in wheezy infants.<br>**Pelkonen, 2013 [7]**	RCT	Daily LTRA (montelukast 4 mg once daily, n=56) VS daily placebo (n=57) for 8 weeks	Mostly (83%) MTWheezers, 17% EVWheezers. (no subgroup analysis performed)	113 (6-24 mo)	Controller therapy.<br><br>Outcomes assessed: Number of symptom free days<br><br>Lung function	No significant differences in changes in symptom-free days between the montelukast and placebo groups (p=0.965).<br><br>No significant between-group differences in the changes in median FRC (p= 0.260), sGaw (p= 0.277) and V’ max (p= 0.364) values after treatment.	

					Airway responsiveness and FeNO<br><br>Use of rescue medication and number of exacerbations	After the treatment period, the methacoline challenge test was successfully performed in a total of 77 children and FeNO measurements undertaken in 71 children. There were no significant differences in changes in these parameters (provocative dose of methacoline causing a 40% fall in forced expiratory flow at FRC and FeNO) between the groups (p=0.513 and p=0.452, respectively)<br><br>No significant differences between the changes in the use of rescue medication between treatment groups (p unspecified).	
The effect of montelukast on respiratory symptoms and lung function in wheezy infants.<br>**Szefler, 2013 [8]**	Open-label, randomized, active-controlled, multicenter study	Daily ICS (budesonide, n=195) vs daily LTRA (montelukast, n=97)<br>(52 weeks)	MTWheezers	202 (2-4 yo)	Controller therapy.<br><br>Outcomes assessed:<br>Rescue oral steroids use<br><br>Additional courses of medication	Patients who required oral steroid therapy in the ICS vs LTRA group:<br>at 12 w 8.6% vs 18.6% (p=0 .044)<br>at 26 w 14.3% vs 27.8% (p=0.029),<br>at 52 w 21.9% versus 37.1% (p=0.022) respectively<br><br>Rate of additional courses of medication (an estimated reduction of 41%) in the ICS vs LTRA group (1.35 vs 2.30; p= 0.003)	
Effectiveness of nebulized beclomethasone in preventing viral wheezing: an RCT.<br>**Clavenna, 2014 [9]**	Randomized, double-blind, parallel group trial	Daily ICS (beclomethasone 400 mcg once daily, n=264) vs placebo (n=264)	EVWheezers During an URTI<br>(10 days of treatment)	525 (1-5 yo)	As needed therapy.<br><br>Outcomes assessed:<br>Development of wheezing<br><br>Extra visits<br><br>Rescue drugs use<br><br>Parents satisfaction rate with treatment	No statistically significant difference in the risk of developing wheezing (6.8% in ICS and 11.1% in placebo, p=0.09)<br><br>No statistically significant difference (p=0.35)<br><br>No statistically significant difference (p=0.23)<br><br>No statistically significant difference (p=0.46)	
Intermittent montelukast in children aged 10 months to 5 years with wheeze (WAIT trial): a multicentre, randomised, placebo-controlled trial.<br>**Nwokoro, 2014 [10]**	Multicentre, parallel-group, randomised, placebo-controlled trial	As-needed LTRA (montelukast, n=669) vs as-needed placebo (n=677)	EVWheezers	1346 (10m-5yo)	As needed therapy.<br><br>Outcomes assessed:<br>Unscheduled medical attendances<br><br>Duration of hospital admission<br><br>Number of wheeze episodes<br><br>Duration of wheeze episodes	No statistically significant difference in mean unscheduled medical attendances between montelukast and placebo groups (p=0.06)<br><br>No statistically significant difference (p=0.40)<br><br>No statistically significant difference (p=0.68)<br><br>No statistically significant difference (p=0.53)	

					Number of courses of oral steroids per year<br><br>Time to first unscheduled medical attendance	Montelukast was effective in difference in reducing the use of corticosteroids (0.26 -SD=0.7- and 0.33 -0.9- courses per child in montelukast and placebo group, respectively; p=0.03)<br><br>No statistically significant difference (p=0.09)	Incidence rate ratio: 0.75 (0.58–0.98)
Efficacy and Safety of Salmeterol/fluticasone Combination Therapy in Infants and Preschool Children with Asthma Insufficiently Controlled by Inhaled Corticosteroids. **Yoshihara, 2016 [11]**	Open-label prospective multicenter study	Daily Salmeterol/fluticasone combination (50/100 mcg)<br>(12 weeks)	MTWheezers (defined as persistently asthmatic in the study), not controlled by daily ICS	35 (6m-5yo)	Controller therapy.<br><br>Outcomes assessed:<br>Nighttime disorder<br><br><br><br><br>Symptoms<br><br><br>Rescue SABA use<br><br><br>Unscheduled clinic visits per week<br><br>Exacerbations due to virus infection<br><br><br><br><br>Asthma control<br><br><br>Quality of life	 <br><br>Median (range) nighttime sleep disorder score:<br>at baseline 2.00 (0.25–9.00)<br>at 4 w 1.00 (0–4.75) (p<0.01[6])<br>at 8 w 0.25 (0–4.00) (p<0.001[6])<br>at 12 w 0.75 (0–3.75) (p<0.001[6])<br><br>Median asthma symptom score:<br>at baseline 4.25 (0.75–32.25)<br>at 12 w 1.25 (0–11.75) (p<0.01)<br><br>Median frequency of SABA use per week:<br>at baseline 1.75 (0–28.0)<br>at 12 w 0 (0–4.25) (p<0.001)<br><br>No statistically significant difference in frequency of unscheduled clinic visits per week between baseline and 12 w endpoint<br><br>Median exacerbations due to virus infection:<br>at baseline 0.25 (0–4.75)<br>at 4 w 0 (0–2.00) (p<0.01[5])<br>at 8 w 0.25 (0–3.75) (p<0.01[5])<br>at 12 w 0.25 (0–4.00) (no statistically significant difference)<br><br>Median asthma control score:<br>at baseline 14.0 (3–23)<br>at 12 w 21.0 (0–24) (p<0.001)<br><br>Median patient QOL score:<br>at baseline was 8.0 (0–20)<br>at 12 w 2.0 (0–24) (p<0.01)	
Early control treatment with montelukast in preschool children with asthma: A randomized controlled trial. **Nagao, 2018 [12]**	Multicenter, open-label, randomized controlled trial	Daily Montelukast (n=47) vs as-needed b2-agonists (n=46) for 48 weeks	MTWheezers (defined as persistently asthmatic in the study)	93 (1-5 yo)	As needed or controller therapy.<br><br>Outcomes assessed:<br>Acute exacerbations<br><br><br><br><br>Step-up treatment	 <br><br><br>Number of acute exacerbations: 0.08/month (0.9/year; LTRA group) and 0.16/month (1.9/year, no controller group)<br><br><br><br>21% in the montelukast group vs 41% in the no-controller group received step-up treatment	 <br><br><br>Hazard ratio 0.41 (Montelukast vs as-needed b2-agonists) 95% CI 0.20 to 0.72<br><br>Hazard ratio 0.45, 95% CI 0.21 to 0.92

					Symptoms-free days	No difference between the groups in mean symptom-free days (p unreported)	
Safety and efficacy of tiotropium in children aged 1-5 years with persistent asthmatic symptoms: a randomised, double-blind, placebo-controlled trial.<br>**Vrijlandt, 2018 [13]**	Multicentre, randomised, double-blind, placebo-controlled, parallel-group, phase 2/3 trial	Daily anticholinergic (tiotropium, 2.5 and 5 mcg, n=36 and 31 respectively) vs placebo (n=34) for 12 weeks	MTWheezers (defined as persistently asthmatic in the study), not controlled by daily ICS	101 (1-5 yo)	Controller therapy:<br><br>Outcomes assessed:<br>Nighttime/daytime symptoms<br><br>Symptoms-free days<br><br>Acute exacerbations<br><br>Rescue albuterol use	No statistically significant difference (p unreported)<br><br>No statistically significant difference (p unreported)<br><br>No statistically significant difference (p=0.46 and p=025 for 2,5 and 5 mcg of tiotropium, respectively)<br><br>No statistically significant difference (p unreported)	
The efficacy and safety of fluticasone/salmeterol compared to fluticasone in children younger than four years of age.<br>**Yoshihara, 2019 [14]**	Phase 4, stratified, controlled, double-blind, parallel-group study	Daily ICS (fluticasone propionate 50-100 mcg twice daily, n=150) vs daily combination (fluticasone propionate/salmeterol 50/25-100/50 mcg twice daily, n=150) for 8 weeks	MTWheezers (defined as asthmatic according to the 2012 Japanese guidelines)	300 (6 m-4 yo)	Controller therapy.<br><br>Outcomes assessed:<br>Mean change in total asthma symptom scores from baseline to the last 7 days of the double-blind period<br><br>Frequency of asthma exacerbations<br><br>Japanese Paediatric Asthma Control (JPAC) scores<br><br>Use of rescue medication over 24 hours	No statistically significant difference (p=0.21)<br><br>No statistically significant difference (p unreported)<br><br>JPAC scores mean change from baseline was greater in the FP/SAL group (0.4 points) than the FP group (−0.3 points), p= 0.04.<br><br>No statistically significant difference (p=0.39)	0.47 (95% CI, 0.14, 1.60)
Role of montelukast in multitrigger wheezers attending chest clinic in Punjab, India<br>**Kahlon, 2021 [15]**	Prospective observational cohort study	Daily LTRA (montelukast 4 mg) for 3 months	MTWheezers	139 (6m-5yo)	Controller therapy.<br><br>Outcomes assessed:<br>Symptoms control	96 children (69%) had complete relief of symptoms, and amongst them, 60 children (62.5%) had symptomatic relief within 2 weeks, 8 children (5.8%) had partial control of symptoms but wanted to continue the treatment due to improved quality of life, 37 children (26.6%) had no change in symptoms.<br><br>10 children had developed symptoms within first 6 months of life and only 40% had relief. 129 children had developed symptoms in later infancy had better control in 75.9% cases (98/129) (p =0.022)	

[1] Percentages refer to the reduction of the Area Under Curve of each treatment compared to placebo
[2] Upper Respiratory Tract Infection
[3] Values are reported as number of patients (% of belonging group at start) in the following order: EVWheezers, MTwheezers, total

[4]Asthma Predictive Index
[5] The RR of the placebo group vs the pooled ciclesonide groups was the only one to reach statistical significance, although all 3 of the ciclesonide groups showed a lower percentage of severe exacerbations than the placebo group
[6] p-value refers to statistical significativity of the difference between each endpoint and baseline

References

1. Bacharier LB, Phillips BR, Zeiger RS, Szefler SJ, Martinez FD, Lemanske RF Jr, Sorkness CA, Bloomberg GR, Morgan WJ, Paul IM, Guilbert T, Krawiec M, Covar R, Larsen G, Mellon M, Moss MH, Chinchilli VM, Taussig LM, Strunk RC; CARE Network. Episodic use of an inhaled corticosteroid or leukotriene receptor antagonist in preschool children with moderate-to-severe intermittent wheezing. J Allergy Clin Immunol. 2008 Dec;122(6):1127-1135.e8. doi: 10.1016/j.jaci.2008.09.029. Epub 2008 Oct 30. PMID: 18973936; PMCID: PMC2753208.
2. Ducharme FM, Lemire C, Noya FJ, Davis GM, Alos N, Leblond H, Savdie C, Collet JP, Khomenko L, Rivard G, Platt RW. Preemptive use of high-dose fluticasone for virus-induced wheezing in young children. N Engl J Med. 2009 Jan 22;360(4):339-53. doi: 10.1056/NEJMoa0808907. PMID: 19164187.
3. Brand PL, Luz García-García M, Morison A, Vermeulen JH, Weber HC. Ciclesonide in wheezy preschool children with a positive asthma predictive index or atopy. Respir Med. 2011 Nov;105(11):1588-95. doi: 10.1016/j.rmed.2011.07.017. Epub 2011 Aug 11. PMID: 21839625.
4. Papi A, Nicolini G, Boner AL, Baraldi E, Cutrera R, Fabbri LM, Rossi GA. Short term efficacy of nebulized beclomethasone in mild-to-moderate wheezing episodes in pre-school children. Ital J Pediatr. 2011 Aug 22;37:39. doi: 10.1186/1824-7288-37-39. PMID: 21859484; PMCID: PMC3170583.
5. Valovirta E, Boza ML, Robertson CF, Verbruggen N, Smugar SS, Nelsen LM, Knorr BA, Reiss TF, Philip G, Gurner DM. Intermittent or daily montelukast versus placebo for episodic asthma in children. Ann Allergy Asthma Immunol. 2011 Jun;106(6):518-26. doi: 10.1016/j.anai.2011.01.017. Epub 2011 Mar 4. PMID: 21624752.
6. Zeiger RS, Mauger D, Bacharier LB, Guilbert TW, Martinez FD, Lemanske RF Jr, Strunk RC, Covar R, Szefler SJ, Boehmer S, Jackson DJ, Sorkness CA, Gern JE, Kelly HW, Friedman NJ, Mellon MH, Schatz M, Morgan WJ, Chinchilli VM, Raissy HH, Bade E, Malka-Rais J, Beigelman A, Taussig LM; CARE Network of the National Heart, Lung, and Blood Institute. Daily or intermittent budesonide in preschool children with recurrent wheezing. N Engl J Med. 2011 Nov 24;365(21):1990-2001. doi: 10.1056/NEJMoa1104647. PMID: 22111718; PMCID: PMC3247621.

7. Pelkonen AS, Malmström K, Sarna S, Kajosaari M, Klemola T, Malmberg LP, Mäkelä MJ. The effect of montelukast on respiratory symptoms and lung function in wheezy infants. Eur Respir J. 2013 Mar;41(3):664-70. doi: 10.1183/09031936.00173411. Epub 2012 Jul 12. PMID: 23060628.
8. Szefler SJ, Carlsson LG, Uryniak T, Baker JW. Budesonide inhalation suspension versus montelukast in children aged 2 to 4 years with mild persistent asthma. J Allergy Clin Immunol Pract. 2013 Jan;1(1):58-64. doi: 10.1016/j.jaip.2012.08.005. Epub 2012 Nov 8. PMID: 24229823.
9. Clavenna A, Sequi M, Cartabia M, Fortinguerra F, Borghi M, Bonati M; ENBe Study Group. Effectiveness of nebulized beclomethasone in preventing viral wheezing: an RCT. Pediatrics. 2014 Mar;133(3):e505-12. doi: 10.1542/peds.2013-2404. Epub 2014 Feb 17. PMID: 24534400.
10. Nwokoro C, Pandya H, Turner S, Eldridge S, Griffiths CJ, Vulliamy T, Price D, Sanak M, Holloway JW, Brugha R, Koh L, Dickson I, Rutterford C, Grigg J. Intermittent montelukast in children aged 10 months to 5 years with wheeze (WAIT trial): a multicentre, randomised, placebo-controlled trial. Lancet Respir Med. 2014 Oct;2(10):796-803. doi: 10.1016/S2213-2600(14)70186-9. Epub 2014 Sep 8. PMID: 25212745; PMCID: PMC4189104.
11. Yoshihara S, Fukuda H, Tamura M, Arisaka O, Ikeda M, Fukuda N, Tsuji T, Hasegawa S, Kanno N, Teraoka M, Wakiguchi H, Aoki Y, Igarashi H, Terada A, Hasegawa M, Manki A. Efficacy and Safety of Salmeterol/fluticasone Combination Therapy in Infants and Preschool Children with Asthma Insufficiently Controlled by Inhaled Corticosteroids. Drug Res (Stuttg). 2016 Jul;66(7):371-6. doi: 10.1055/s-0042-108852. Epub 2016 Jun 6. PMID: 27273710.
12. Nagao M, Ikeda M, Fukuda N, Habukawa C, Kitamura T, Katsunuma T, Fujisawa T; LePAT (Leukotriene and Pediatric Asthma Translational Research Network) investigators. Early control treatment with montelukast in preschool children with asthma: A randomized controlled trial. Allergol Int. 2018 Jan;67(1):72-78. doi: 10.1016/j.alit.2017.04.008. Epub 2017 May 16. PMID: 28526210.
13. Vrijlandt EJLE, El Azzi G, Vandewalker M, Rupp N, Harper T, Graham L, Szefler SJ, Moroni-Zentgraf P, Sharma A, Vulcu SD, Sigmund R, Chawes B, Engel M, Bisgaard H. Safety and efficacy of tiotropium in children aged 1-5 years with persistent asthmatic symptoms: a randomised, double-blind, placebo-controlled trial. Lancet Respir Med. 2018 Feb;6(2):127-137. doi: 10.1016/S2213-2600(18)30012-2. Epub 2018 Jan 18. PMID: 29361462.

14. Yoshihara S, Tsubaki T, Ikeda M, Lenney W, Tomiak R, Hattori T, Hashimoto K, Soutome T, Kato S. The efficacy and safety of fluticasone/salmeterol compared to fluticasone in children younger than four years of age. Pediatr Allergy Immunol. 2019 Mar;30(2):195-203. doi: 10.1111/pai.13010. Epub 2019 Feb 6. PMID: 30556939; PMCID: PMC6850202.
15. Kahlon GK, Pooni PA, Bhat D, Dhooria GS, Bhargava S, Arora K, Gill KS. Role of montelukast in multitrigger wheezers attending chest clinic in Punjab, India. Pediatr Pulmonol. 2021 Aug;56(8):2530-2536. doi: 10.1002/ppul.25522. Epub 2021 Jun 8. PMID: 34102024.

2) **Management of the acute attack**

PICO question 2. Are inhaled short-acting β2-agonists (SABA) useful in exacerbation of preschool wheezing?

Patient or population: children with preschool wheezing

Setting: primary care/emergency department

Intervention: inhaled beta2-agonists for acute attack

Comparison: placebo

Search strategy:

((child, preschool[MeSH Terms]) OR (toddler*)) AND ((Respiratory Sounds[Mesh]) OR (wheez*) OR ("Asthma"[Mesh:NoExp])) AND ((Anti-Asthmatic Agents[Mesh]) OR (Adrenergic beta-2 Receptor Agonists[Mesh]) OR ("Bronchodilator Agents"[Mesh]) OR (albuterol[MeSH Terms]) OR (salbutamol)) AND (english[Filter]) AND (2008:2021[pdat])

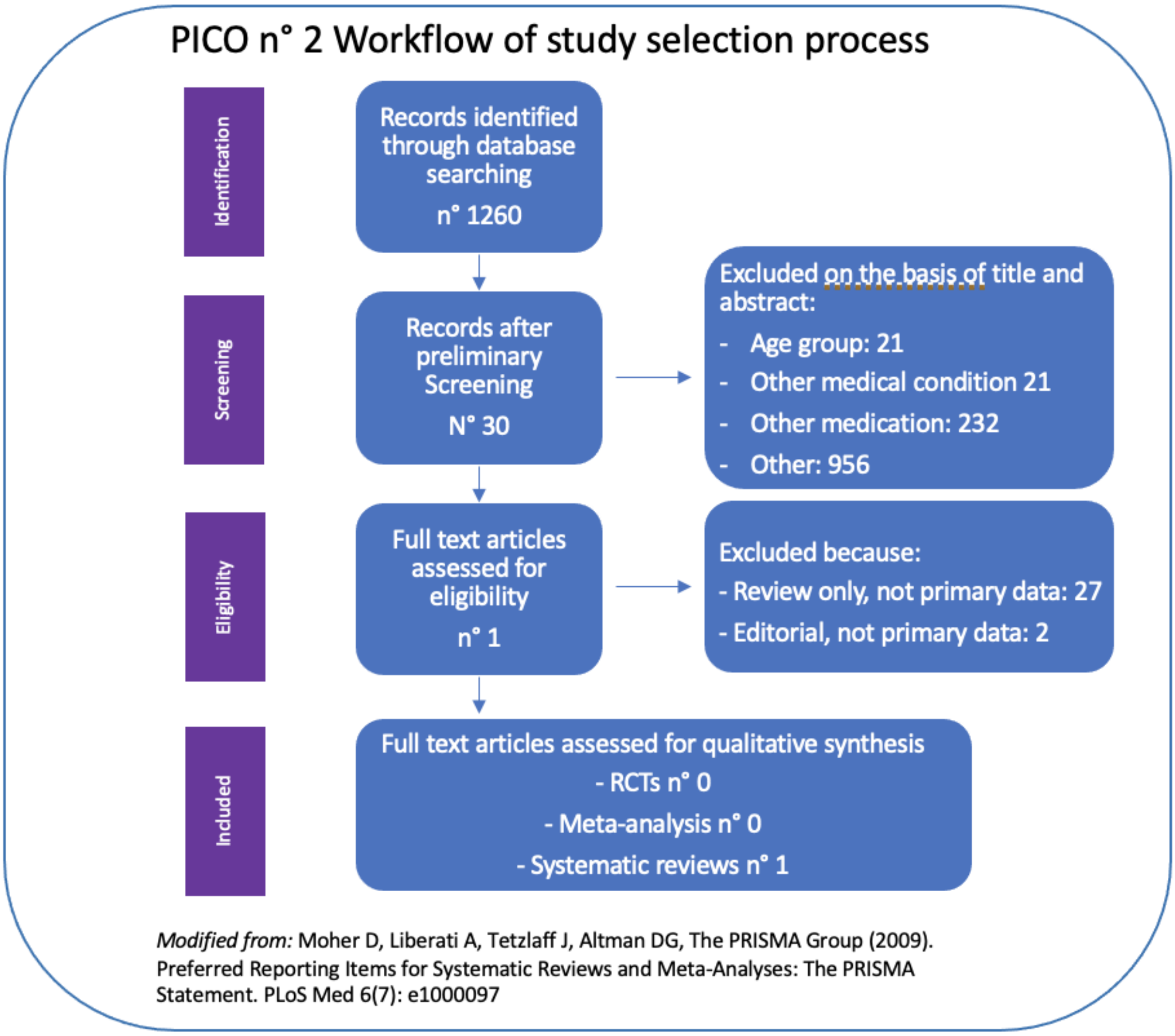

Title of the study, first author, year [ref]	Type of study	Study design	N of patients (age)	Experiments/mechanisms assessed	Outcomes (primary and secondary)	Results	Effect [Relative risk (RR) or odds ratio (OR) + confidence interval (CI 95%)]
Inhaled short-acting bronchodilators for managing emergency childhood asthma: an overview of reviews. **Pollock, 2017 [1]**	Systematic review	Inhaled short-acting bronchodilators for managing childhood asthma exacerbation in ED	5526 (0-18 years)				
			28 (from 1 trial) (0-3 years)	SABA vs placebo	Primary outcome: Hospital admission	Not significant (low quality evidence)	RR 1.73 (0.34-8.81)
					Secondary outcome:		
					Clinical severity score	Favors SABA	SMD -1.31 (-2.14 to -0.48)
					Respiratory rate	Favors SABA	MD -5.10 (-9.45 to -0.75)
					Oxygen saturation	Favors SABA	MD 1.60 (0.33 to 2.87)

OR odds ratio, ED emergency department, RR risk ratio, SMD standardized mean difference, MD mean difference.

References

1. Pollock M, Sinha IP, Hartling L, Rowe BH, Schreiber S, Fernandes RM. Inhaled short-acting bronchodilators for managing emergency childhood asthma: an overview of reviews. Allergy. 2017 Feb;72(2):183-200. doi: 10.1111/all.13039. Epub 2016 Oct 5. PMID: 27588581.

PICO question 3. A are oral cortcosteroids (OCS) useful in exacerbation of preschool wheezing?

Patient or population: children with preschool wheezing

Setting: primary care/emergency department

Intervention: oral corticosteroid for acute attack

Comparison: placebo

Search strategy:

((child, preschool[MeSH Terms]) OR (toddler*)) AND ((Respiratory Sounds[Mesh]) OR (wheez*) OR ("Asthma"[Mesh:NoExp])) AND ((Administration, Oral[Mesh]) OR (oral) OR (systemic)) AND ((Adrenal Cortex Hormones[Mesh]) OR (*steroid*) OR (betamethasone) OR (prednisone) OR (dexamethasone)) AND (english[Filter]) AND (2008:2021[pdat])

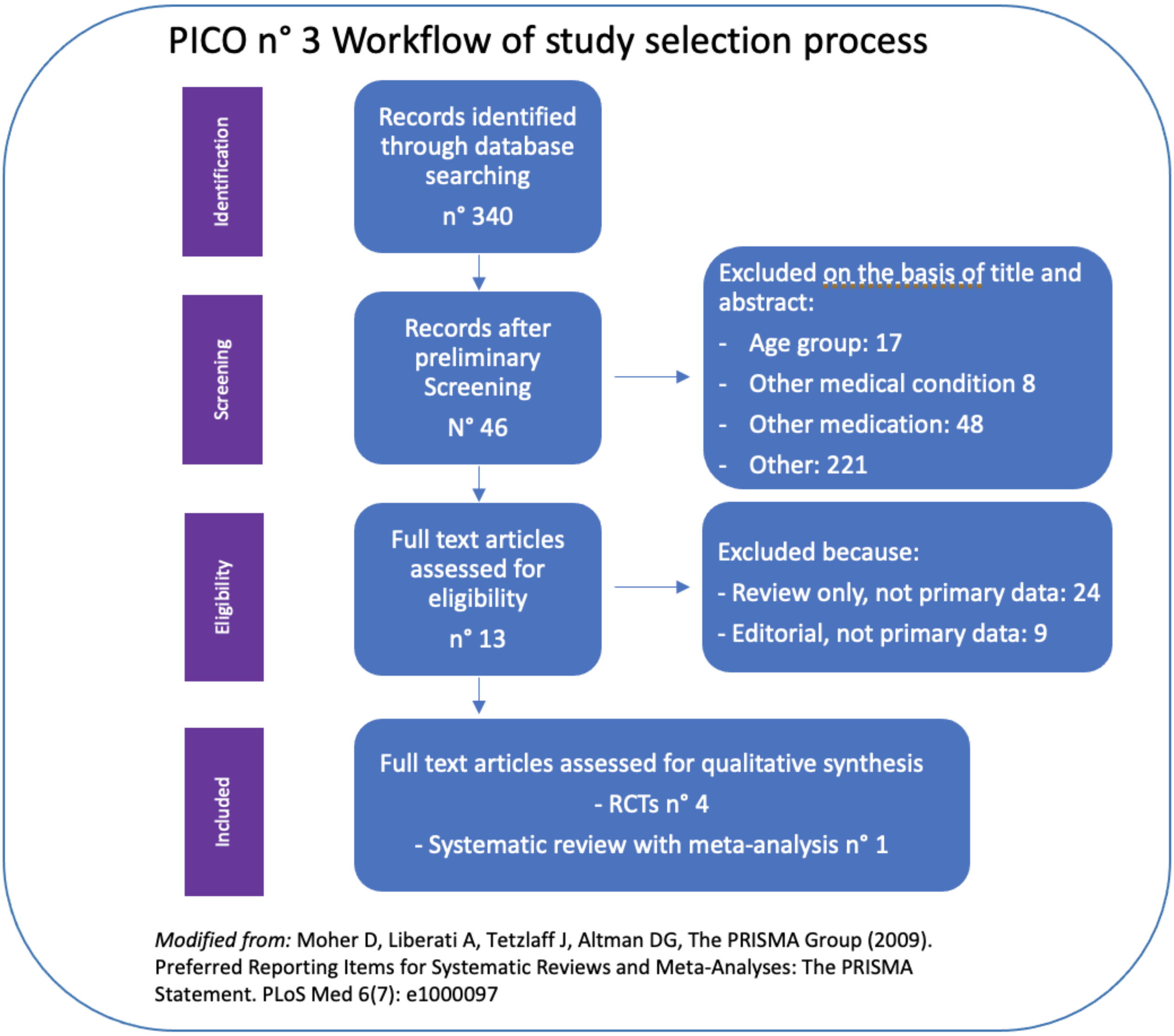

Title of the study, first author, year [ref]	Type of study	Study design	N of patients (age)	Experiments/mechanisms assessed	Outcomes (primary and secondary)	Results	Effect [Relative risk (RR) or odds ratio (OR) + confidence interval (CI 95%)]
Oral prednisolone for preschool children with acute virus-induced wheezing. **Panickar, 2009 [1]**	Randomized, double-blind, placebo-controlled trial	Oral prednisolone (n 343) vs placebo (n 344) during a *mild-to-moderate attack* of wheezing associated with a viral infection	687 (10-60 months)	Prednisolone 10 mg once a day (10-24 months), 10 mg once a day (>24 months) vs placebo for 5 days	Primary outcome		
					Interval between presentation and signoff for discharge	No significant difference (p = 0.16)	0.89 (0.76 - 1.05)
					Interval between presentation and actual discharge	No significant difference (p = 0.18)	0.90 (0.77 - 1.05)
					Secondary outcome		
					PRAM score at 4,12,24 hrs	No significant difference	-0.29 (-0.65 - 0.06), 0.2 (-0.24 - 0.64), -0.06 (-0.57 - 0.51)
					Albuterol use (total metered-dose inhaler actuations)	No significant difference	-14.08 (-26.62 - 1.54)
					7-day symptom score daytime	No significant difference	−0.06 (−0.18 - 0.07)
					7-day symptom score nighttime	No significant difference	−0.14 (−0.29 - 0.01)
Do oral corticosteroids reduce the severity of acute lower respiratory tract illnesses in preschool children with recurrent wheezing? **Beigelman, 2013 [2]**	Post hoc and replication analyses of 2 multicenter, double-blind, randomized, placebo-controlled trial	Oral prednisolone vs placebo in *severe intermittent wheezing* during acute episodes of severe LRTIs	215 (12-59 months) + 278 (12-53 months)	Prednisolone once-daily dose at 2 mg/kg/d for 2 days, followed by 1 mg/ kg/d for an additional 2 days, prescribed as rescue treatment according to predefined protocol	Primary outcome		
					AUC of total symptom scores among the more severe episodes.	No significant difference (p = 0.46, p = 0.46)	
					Secondary outcome		
					AUC cough score	No significant difference (p = 0.83, p = 0.83)	
					AUC wheeze score	No significant difference (p = 0.35, p = 0.05)	
					AUC trouble breathing score	No significant difference (p = 0.15, p = 0.76)	
					AUC interference with activity score	No significant difference (p = 0.79, p = 0.53)	

Oral prednisolone in preschool children with virus-associated wheeze: a prospective, randomized, double-blind, placebo-controlled trial. **Foster, 2018 [3]**	Non-inferiority, prospective, randomized, double-blind, placebo-controlled trial	Oral prednisolone (n 305) vs placebo (n 300) in virus-associated wheeze	605 (24-72 months)	Prednisolone once-daily dose at 1 mg/kg/d vs placebo for 3 days	Length of stay	Significant difference (p 0.0227)	0.79 (0.64 – 0.97)
Impact of oral corticosteroids on respiratory outcomes in acute preschool wheeze: a randomized clinical trial. **Wallace, 2021 [4]**	Randomized, double-blind placebo-controlled trial	Oral prednisolone (n 238) vs placebo (n 239) in wheeze associated with respiratory illness	477 (24-59 months)	Prednisolone once-daily dose at 2 mg/kg/d (max 40 mg) vs placebo for 3 days	<u>Primary outcome</u>		
					PRAM score at 24 hrs	No significant difference (p = 0.09)	−0.39 (−0.84 - 0.06)
					<u>Secondary outcome</u>		
					Length of ED stay for discharged patients	No significant difference (p = 0.06)	−0.57 (−1.15 - 0.02)
					Requirement for hospital admission	No significant difference (p = 0.05)	OR 0.67 (0.45 to 1.01)
					Length of inpatient stay	No significant difference (p = 0.27)	−2.9 (−7.8 - 2.4)
					MDI doses of salbutamol in first 48 hours	No significant difference (p = 0.27)	0 (−6 - 0)
Efficacy of oral corticosteroids in the treatment of acute wheezing episodes in asthmatic preschoolers: Systematic review with meta-analysis. **Castro-Rodriguez, 2016 [5]**	Systematic review with meta-analysis	Oral corticosteroids versus placebo in preschoolers presenting with acute asthma/wheezing exacerbations.	1733 (<6 years)		Hospital admission	No significant difference	RR 1.00 (0.49–2.05)
					Additional Course of Systemic Corticosteroids	No significant difference	RR 0.74 (0.40–1.34)
					Unscheduled Visits	No significant difference	RR 0.73 (0.35–1.52)

OR odds ratio, ED emergency department, RR risk ratio, SMD standardized mean difference, MD mean difference, PRAM Preschool Respiratory Assessment Measure, hrs hours, LRTIs lower respiratory tract illnesses, AUC area under the curve.

References

1. Panickar J, Lakhanpaul M, Lambert PC, Kenia P, Stephenson T, Smyth A, Grigg J. Oral prednisolone for preschool children with acute virus-induced wheezing. N Engl J Med. 2009 Jan 22;360(4):329-38. doi: 10.1056/NEJMoa0804897. PMID: 19164186.
2. Beigelman A, King TS, Mauger D, Zeiger RS, Strunk RC, Kelly HW, Martinez FD, Lemanske RF Jr, Rivera-Spoljaric K, Jackson DJ, Guilbert T, Covar R, Bacharier LB; Childhood Asthma Research and Education Network of National Heart, Lung, and Blood Institute. Do oral corticosteroids reduce the severity of acute lower respiratory tract illnesses in preschool children with recurrent wheezing? J Allergy Clin Immunol. 2013 Jun;131(6):1518-25. doi: 10.1016/j.jaci.2013.01.034. Epub 2013 Mar 14. PMID: 23498594; PMCID: PMC3810170.
3. Foster SJ, Cooper MN, Oosterhof S, Borland ML. Oral prednisolone in preschool children with virus-associated wheeze: a prospective, randomised, double-blind, placebo-controlled trial. Lancet Respir Med. 2018 Feb;6(2):97-106. doi: 10.1016/S2213-2600(18)30008-0. Epub 2018 Jan 17. Erratum in: Lancet Respir Med. 2018 Jan 31;: PMID: 29373235.
4. Wallace A, Sinclair O, Shepherd M, Neutze J, Trenholme A, Tan E, Brabyn C, Bonisch M, Grey N, Johnson DW, McNamara D, Thompson JMD, Asher I, Dalziel SR. Impact of oral corticosteroids on respiratory outcomes in acute preschool wheeze: a randomised clinical trial. Arch Dis Child. 2021 Apr;106(4):339-344. doi: 10.1136/archdischild-2020-318971. Epub 2020 Oct 16. Erratum in: Arch Dis Child. 2021 Mar 30;: PMID: 330673
5. Castro-Rodriguez JA, Beckhaus AA, Forno E. Efficacy of oral corticosteroids in the treatment of acute wheezing episodes in asthmatic preschoolers: Systematic review with meta-analysis. Pediatr Pulmonol. 2016 Aug;51(8):868-76. doi: 10.1002/ppul.23429. Epub 2016 Apr 13. PMID: 27074244; PMCID: PMC5007060.

PICO question 4. Are inhaled steroids useful in exacerbation of preschool wheezing?

Patient or population: children with preschool wheezing

Setting: primary care

Intervention: daily ICS

Comparison: placebo

Search strategy:

("child, preschool"[MeSH Terms] OR "toddler"[All Fields]) AND ("respiratory sounds"[MeSH Terms] OR "wheez*"[All Fields]) AND ("intermittent steroid*" OR "inhaled steroid*" OR "ICS" OR "inhaled corticosteroid"[All Fields] OR "beclomethasone"[MeSH Terms] OR "fluticasone"[MeSH Terms] OR "Adrenal Cortex Hormones/therapeutic use"[MAJR]) AND ((2008:2021[pdat]) AND (english[Filter]))

PICO n° 4 Workflow of study selection process

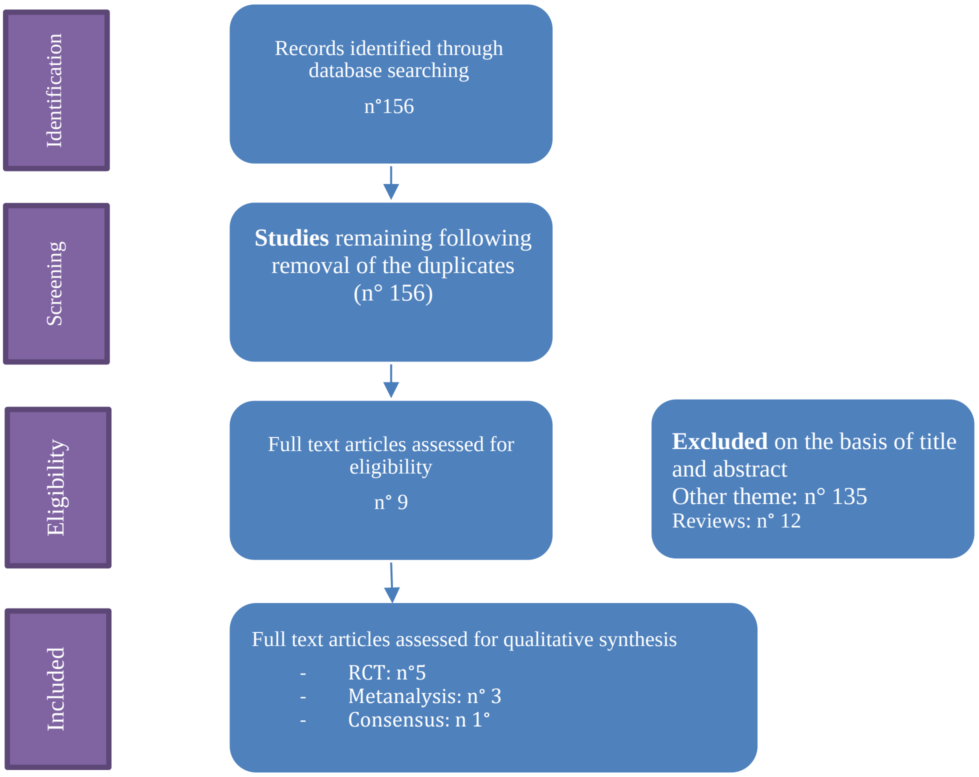

Modified from: Moher D, Liberati A, Tetzlaff J, Altman DG, The PRISMA Group (2009). Preferred Reporting Items for Systematic Reviews and Meta-Analyses: The PRISMA Statement. PLoS Med 6(7): e1000097

Title of the study, first author, year [ref]	Type of study	Study design	N of patients, age	Experiments/mechanisms assessed	Outcomes (primary and secondary)	Results	Effect [Relative risk (RR) or odds ratio (OR) + confidence interval (CI 95%)]
Daily or Intermittent Budesonide in Preschool Children with Recurrent Wheezing. **Zeiger, 2011 [1]**	randomized, double-blind, parallel-group trial	Children were randomly assigned to receive either an intermittent high-dose regimen of budesonide (1 mg twice daily for 7 days, starting early during a predefined respiratory tract illness (n 139) or a daily low-dose regimen of budesonide(0.5 mg nightly) (n 139)<br>and as-needed albuterol	278 (12-53 months)	Daily low dose of budesonide<br>VS<br>Intermittent high-dose budesonide during respiratory tract illness	Frequency of exacerbations (rate per patient-year)	No difference	Daily: 0.97 CI 0.76 to 1.22<br>Intermittent: 0.95 CI, 0.75 to 1.20
					Time to the first exacerbation	No difference	Hazard ratio, 0.97; CI, 0.76 to 1.22; P=0.87
					Rate of treatment failure,	No difference	P=0.12
					No. of treatments for respiratory tract illness	No difference	Event rate/Person year:<br>Intermittent: 3.61 CI (3.13 to 4.16)<br>Daily: 3.27 CI (2.82 to 3.79)
					No. of urgent care visits for asthma	No difference	Event rate/Person year:<br>Intermittent: 2.37 (1.89 to 2.97)<br>Daily: 2.40 (1.91 to 3.02)<br>relative rate: 0.99 (0.72 to 1.35)
					Respiratory tract illnesses in which prednisolone was administered	No difference	Proportion:<br>intermittent: 0.24 CI (0.19 to 0.29)<br>Daily: 0.26 (0.21 to 0.32)<br>Relative proportion: 0.90 (0.68 to 1.19)
					No. of days absent from work, school, or day care	No difference	Event rate/Person year:<br>Intermittent: 2.72 (2.00 to 3.70)<br>Daily: 3.02 (2.22 to 4.12)<br>Relative rate: 0.90 (0.59 to 1.37)
					Days with albuterol use (%)	No difference	Mean value<br>Intermittent: 6 CI (5 to 7)<br>Daily : 5 CI (4 to 6)<br>Mean difference<br>0.4 (−1.0 to 2.0)
					Episode-free days (%)	No difference	Mean value<br>Intermittent: 78 (75 to 80)<br>Daily : 78 (76 to 81)<br>Mean difference<br>−0.7 (−4.0 to 2.0)
Short term efficacy of nebulized beclomethasone in mild-to-moderate wheezing	Double-blind, double-dummy, randomized, parallel-group trial	Post hoc analysis of a previous study on children with multiple trigger wheeze. A group (n 110) received	166 (1-4 years)	High dose beclomethasone (110) vs	Symptom-free days (mean percentage)	Beclomethasone (54.7%), > as needed salbutamol (40.5 %)	P = 0.033; [OR 2.65; 1.08-6.51]

episodes in pre-school children. **Papi, 2011 [2]**		daily placebo plus a high doses of beclomethasone 800 µg/1600 µg salbutamol and one group (n 56) received daily placebo and 2500 µg of salbutamol during the acute respiratory event.		Regular placebo (56) + as needed salbutamol for 1 week	Daytime rescue medication, no. of uses	Beclomethasone (0.26) = as needed salbutamol (0.34)	P = 0.366
					Mean coughing score	Beclomethasone (0.11), > as needed salbutamol (0.39)	P = 0.048
Preemptive use of high-dose fluticasone for virus-induced wheezing in young children. **Ducharme, 2009 [3]**	Parallel-group, randomized, placebo-controlled trial with triple blinding	As-needed ICS (fluticasone propionate 750 mcg twice daily, n=62) vs placebo (n=67)	EV Wheezers	129 (1-6 years)	Outcomes:<br>Oral corticosteroids use<br><br>Symptoms control<br><br>Rescue β2-agonist use	8 vs 18% URTIs[2] requiring systemic corticosteroids<br><br>5.66±4.37 vs 6.88±5.1 days with asthma-like symptoms during URTIs<br><br>4 (2 to 7) vs 6 (3 to 9) days of rescue β2-agonist use during URTIs	OR (ICS vs placebo) 0.49, CI 95% 0.30 to 0.83<br><br>Rate Ratio (ICS vs placebo) 0.82 CI 95% 0.71 to 0.95<br><br>Rate Ratio (ICS vs placebo) 0.80, CI 95% 0.68 to 0.94
Episodic use of an inhaled corticosteroid or leukotriene receptor antagonist in preschool children with moderate-to-severe intermittent wheezing. **Bacharier, 2008 [4]**	Randomized double-blind, placebo-controlled trial	Administration of either budesonide inhalation (n 96), montelukast (n 95) or placebo (n 47) for 1 week in addition to albuterol in children with *moderate-to-severe intermittent wheezing*	238 (12-59 months)	7 days of inhaled budesonide (1 mg twice daily) vs montelukast 4 mg daily vs placebo	Primary outcome:<br>Proportion of episode-free days (EFDs) during 12 months	No difference among: budesonide, montelukast or placebo	0.76 (0.70-0.81)<br>0.73 (0-66-0.79)<br>0.74 (0.65-0.81)
					Secondary outcomes:		
					Oral corticosteroid use	No difference (p = 0.15)	
					Health care use	No difference (p = 0.98)	
					Quality of life	No difference (p > 0.16)	
					Linear growth	No difference (p = 0.59)	
					Trouble breathing	Improved with budesonide (p = 0.003) and montelukast (p = 0.003)	Score AUC: 4.2 (3.1-5.2)<br>4.2 (3.1-5.3)
					Interference with activity scores	Improved with budesonide (p = 0.01) and montelukast (p = 0.001)	Score AUC: 4.8 (3.7-5.9)<br>4.3 (3.1-5,4)
					Mean total symptoms score	Improved with budesonide (p = 0.001) and montelukast (p = 0.003)	Score AUC:<br>5.8 (4.8-6.7)<br>5.4 (4.4-6.4)

Effectiveness of nebulized beclomethasone in preventing viral wheezing: an RCT.<br>**Clavenna, 2014 [5]**	Randomized, double-blind, parallel group trial	Daily ICS (beclomethasone 400 mcg once daily, n=264) vs placebo (n=264)	EVWheezers During an URTI<br>(10 days of treatment)	525 (1-5 yo)	As needed therapy.<br><br>Outcomes assessed:<br>Development of wheezing<br><br>Extra visits<br><br>Rescue drugs use<br><br>Parents satisfaction rate with treatment	No statistically significant difference in the risk of developing wheezing (6.8% in ICS and 11.1% in placebo, p=0.09)<br><br>No statistically significant difference (p=0.35)<br><br>No statistically significant difference (p=0.23)<br><br>No statistically significant difference (p=0.46)	
Nebulized Inhaled Corticosteroids in Asthma Treatment in Children ≤5 Years of Age: A Systematic Review and Global Expert Analysis<br>**Murphy, 2020 [6]**	Systematic review	ICS for the management of acute asthma (n°10)<br>-budesonide (n = 7),<br>-fluticasone (n = 2),<br>-flunisolide (n = 1),<br>-beclomethasone (n = 1),<br>-dexamethasone (n = 1)	1018	Budesonide 2 times/day VS placebo (age 7-72 month)	Hours of hospitalization	Significantly shorter in the ICS group	44 hours (ICS group) vs 80 hours (placebo group); P = 0.01
				Budesonide 2 mg single dose VS placebo (age 6 month-18 years)	Pulmonary index score after 1 hour		-ICS: 5.0<br>-placebo 6.0 (P = 0.07)
				Budesonide 2 mg single dose VS placebo (age 2 -18 years)		No significant difference in respiratory rate, heart rate, oxygen saturation, or number of hospital admissions	Asthma score -3 in both groups (P = 0.64)
				Beclomethasone 2 times/day VS placebo (age 1-4 years)	Symptom-free days	more symptom-free days in Beclomethasone group	Rate<br>-ICS: 54.7%<br>-placebo 40.5%,; P = 0.012
				Budesonide 1 mg 2 times/day.<br>Vs Prednisolone 0.5 mg/Kg 3/day (<3 years)			
				Budesonide 800 µg dose at half-hour intervals (3 doses) vs oral Prednisolone 2 mg/kg (age 1-12 years)		greater improvement in mean respiratory rate, heart rate, PIS, and respiratory distress assessment scores for budesonide group	all P < 0.05

				Fluticasone 500 µg 4 times/day. vs oral methyl- Prednisolone 1 mg/kg/day (4 days) → 0.5 mg/kg/day (3 days)<br>(age 1-16 years)	Pulmonary index score	No difference	Mean PIS decreased from 8 to 1 in both groups (both P < 0.0001)
				Dexamethasone 1.5 mg/kg vs 2 mg/kg oral prednisone<br>(age 1-17 years)	Hospitalization<br><br>Discharge from ER after 2 h	Fewer patient in the dexamethasone group<br><br>Rapid onset of action Dexamethasone> prednisone	-dexamethasone: 21%<br>-prednisone:31%<br>(P = 0.26)<br><br>-dexamethasone: 23%<br>-prednisone:7%<br>(P = 0.02)
				budesonide 500 µg 2 times/day vs Fluticasone 250 µg 2 times/day<br>(age 4-15 years)	Morning PEF		-fluticasone: PEF increased 44L/min<br>-budesonide: PEF increased 39L/min<br>(P = 0.032)
				Budesonide 0.5 mg 2 times/day for 7 days → 0.25 mg 2 times/day for 14 days<br>Vs<br>Flunisolide 40 µg 2 times/day for 7 days → 20 µg 2 times/day for 14 days<br>(3-5 years)	Symptome score	Flunisolide better than budesonide at day 7 but not at day 21	(P < 0.05)
		ICS for the management of maintenance therapy (n°9)	1681				
Preventing Exacerbations in Preschoolers With Recurrent Wheeze: A Meta-analysis.<br>**Kaiser, 2016 [7]**	Meta-analysis	In consultation with a medical librarian, 3 search strategies for 3 databases were created Studies were considered eligible for inclusion if they met criteria regarding population, intervention and comparator, outcomes, and study design. Studies that included only children <2 years were excluded because of the potential overlap with	Children with asthma or recurrent wheezing (≥2 episodes/ year)<br>(0-6 years)	Studies comparing the following interventions were included: daily ICS versus placebo, intermittent ICS versus placebo, daily ICS versus intermittent ICS, or any regimen of ICS versus any regimen of montelukast. Any	22 studies (N = 4550) were included.<br>15 studies (N = 3278) compared daily ICS with placebo and showed reduced exacerbations with daily medium-dose ICS. Subgroup analysis of children with persistent asthma showed reduced exacerbations with daily ICS compared with placebo (8 studies, N =	Daily ICS with placebo<br><br>Daily ICS compared with montelukast	- RR 0.70 (95% CI 0.61–0.79)<br><br>- RR 0.56 (95% CI 0.46–0.70)<br>- RR 0.59 (95% CI, 0.38–0.92)

		bronchiolitis in this age group.		studies that reported on outcome of severe wheezing exacerbations necessitating systemic (oral or intravenous) corticosteroid were included. Severe exacerbations were chosen as our primary outcome because they are a patient-important outcome that have significant consequences for children, caregivers, and the health care system. Only randomized controlled trials were included. Guidelines, reviews, commentaries, abstracts, and letters to editors were reviewed to identify any primary data; however, these publication types were not included because of lack of peer review and inability to judge bias.	2505) and daily ICS compared with montelukast (1 study, N = 202). Subgroup analysis of children with intermittent asthma or viral-triggered wheezing showed reduced exacerbations with preemptive high-dose intermittent ICS compared with placebo (5 studies, N = 422). LIMITATIONS: More studies are needed that directly compare these strategies. There is strong evidence to support daily ICS for preventing exacerbations in preschool children with recurrent wheeze, specifically in children with persistent asthma.	Intermittent steroids vs placebo	- RR 0.65 (95% CI 0.51–0.81)
					Trouble breathing	Improved with budesonide (p = 0.003) and montelukast (p = 0.003)	Score AUC: 4.2 (3.1-5.2)<br>4.2 (3.1-5.3)
					Interference with activity scores	Improved with budesonide (p = 0.01) and montelukast (p = 0.001)	Score AUC: 4.8 (3.7-5.9)<br>4.3 (3.1-5,4)
					Mean total symptoms score	Improved with budesonide (p = 0.001) and montelukast (p = 0.003)	Score AUC:<br>5.8 (4.8-6.7)<br>5.4 (4.4-6.4)
Intermittent inhaled corticosteroid therapy versus placebo for persistent asthma	Meta-analysis	4 preschool child trials	490 preschool-child	As required ICS<br>Vs placebo	Exacerbations requiring oral corticosteroids	ICS<placebo	OR 0.48 (0.31 to 0.73) CI 95%

in children and adults (Review)<br>Cochrane Database of Systematic Reviews<br>**Chong J, 2015 [8]**					Serious adverse events	ICS<placebo	OR 0.42 (0.17 to 1.02) CI 95%
					Hospitalisations	ICS<placebo	OR 0.73 (0.23 to 2.29) CI 95%
					Quality of Life	The mean quality of life in the intervention groups was 3.28 higher	
					Day time asthma score	The mean daytime asthma score in the intervention groups was 0.35 standard deviations lower	
					Night time asthma score	The mean nighttime asthma score in the intervention groups was 0.28 standard deviations lower	-0.28 [-0.50, -0.06
					Adverse events	ICS< placebo	OR 0.86 (0.42 to 1.75) CI 95%

References

1. Zeiger RS, Mauger D, Bacharier LB, Guilbert TW, Martinez FD, Lemanske RF Jr, Strunk RC, Covar R, Szefler SJ, Boehmer S, Jackson DJ, Sorkness CA, Gern JE, Kelly HW, Friedman NJ, Mellon MH, Schatz M, Morgan WJ, Chinchilli VM, Raissy HH, Bade E, Malka-Rais J, Beigelman A, Taussig LM; CARE Network of the National Heart, Lung, and Blood Institute. Daily or intermittent budesonide in preschool children with recurrent wheezing. N Engl J Med. 2011 Nov 24;365(21):1990-2001. doi: 10.1056/NEJMoa1104647. PMID: 22111718; PMCID: PMC3247621.
2. Papi A, Nicolini G, Boner AL, Baraldi E, Cutrera R, Fabbri LM, Rossi GA. Short term efficacy of nebulized beclomethasone in mild-to-moderate wheezing episodes in pre-school children. Ital J Pediatr. 2011 Aug 22;37:39. doi: 10.1186/1824-7288-37-39. PMID: 21859484; PMCID: PMC3170583
3. Ducharme FM, Lemire C, Noya FJ, Davis GM, Alos N, Leblond H, Savdie C, Collet JP, Khomenko L, Rivard G, Platt RW. Preemptive use of high-dose fluticasone for virus-induced wheezing in young children. N Engl J Med. 2009 Jan 22;360(4):339-53. doi: 10.1056/NEJMoa0808907. PMID: 19164187.
4. Bacharier LB, Phillips BR, Zeiger RS, Szefler SJ, Martinez FD, Lemanske RF Jr, Sorkness CA, Bloomberg GR, Morgan WJ, Paul IM, Guilbert T, Krawiec M, Covar R, Larsen G, Mellon M, Moss MH, Chinchilli VM, Taussig LM, Strunk RC; CARE Network. Episodic use of an inhaled corticosteroid or leukotriene receptor antagonist in preschool children with moderate-to-severe intermittent wheezing. J Allergy Clin Immunol. 2008 Dec;122(6):1127-1135.e8. doi: 10.1016/j.jaci.2008.09.029. Epub 2008 Oct 30. PMID: 18973936; PMCID: PMC2753208.
5. Clavenna A, Sequi M, Cartabia M, Fortinguerra F, Borghi M, Bonati M; ENBe Study Group. Effectiveness of nebulized beclomethasone in preventing viral wheezing: an RCT. Pediatrics. 2014 Mar;133(3):e505-12. doi: 10.1542/peds.2013-2404. Epub 2014 Feb 17. PMID: 24534400.
6. Murphy KR, Hong JG, Wandalsen G, Larenas-Linnemann D, El Beleidy A, Zaytseva OV, Pedersen SE. Nebulized Inhaled Corticosteroids in Asthma Treatment in Children 5 Years or Younger: A Systematic Review and Global Expert Analysis. J Allergy Clin Immunol Pract. 2020 Jun;8(6):1815-1827. doi: 10.1016/j.jaip.2020.01.042. Epub 2020 Jan 30. PMID: 32006721.

7. Kaiser SV, Huynh T, Bacharier LB, Rosenthal JL, Bakel LA, Parkin PC, Cabana MD. Preventing Exacerbations in Preschoolers With Recurrent Wheeze: A Meta-analysis. Pediatrics. 2016 Jun;137(6):e20154496. doi: 10.1542/peds.2015-4496. PMID: 27230765.
8. Chong J, Haran C, Chauhan BF, Asher I. Intermittent inhaled corticosteroid therapy versus placebo for persistent asthma in children and adults. Cochrane Database Syst Rev. 2015 Jul 22;2015(7):CD011032. doi: 10.1002/14651858.CD011032.pub2. PMID: 26197430; PMCID: PMC8676065.

PICO question 5. Are antibiotics useful in exacerbation of preschool wheezing?

Patient or population: children with preschool wheezing

Setting: primary care/hospital

Intervention: antibiotics use in exacerbation

Comparison: usual care with salbutamol

Search strategy:

("child, preschool"[MeSH Terms] OR "toddler*"[All Fields]) AND ("respiratory sounds"[MeSH Terms] OR "wheez*"[All Fields] OR "Asthma"[MeSH Terms:noexp]) AND ("anti-bacterial agents"[MeSH Terms] OR "antibiotic"[All Fields] OR "antibiotics"[All Fields]) AND "english"[Language] AND 2008/01/01:2021/12/31[Date - Publication]

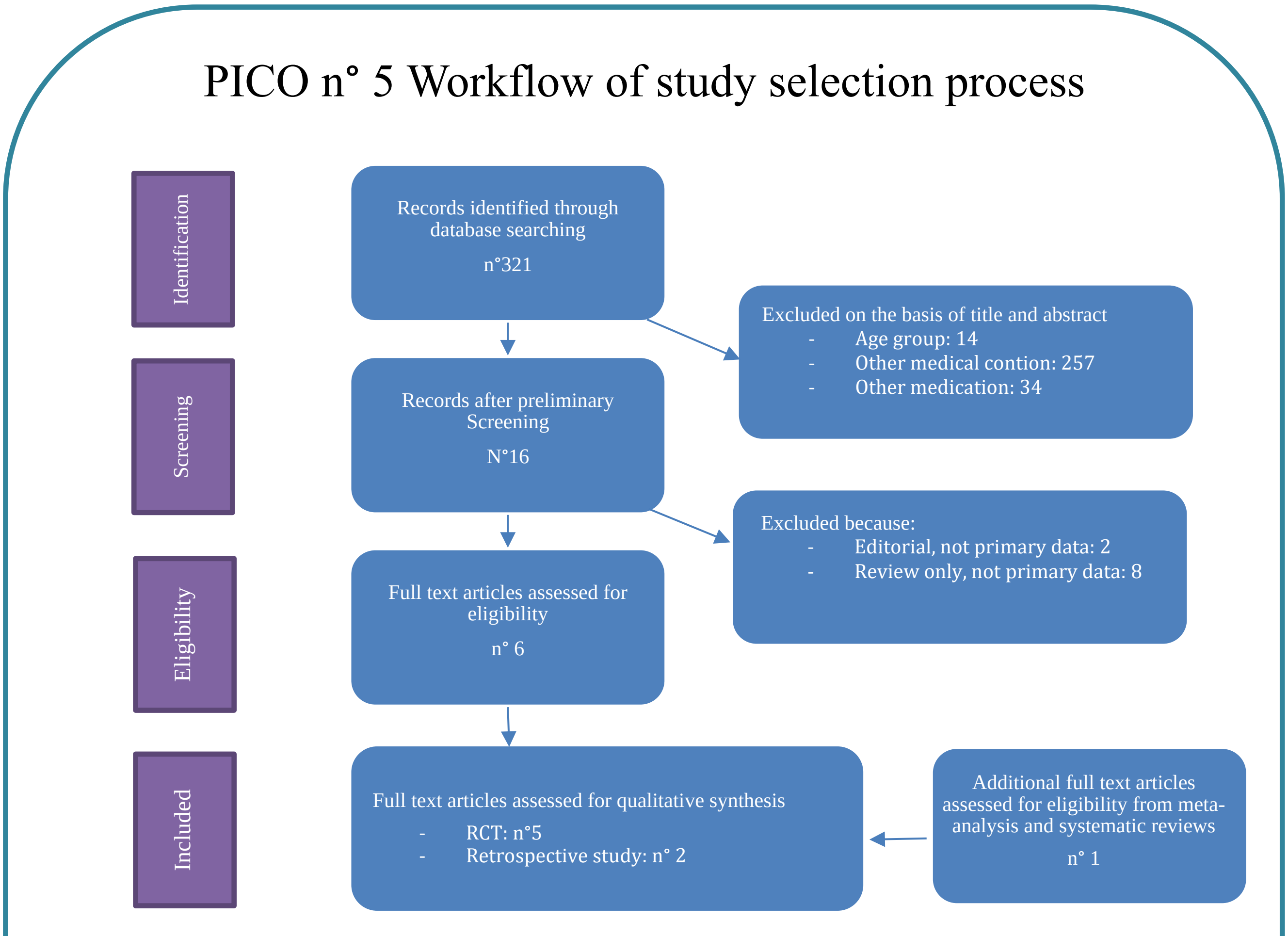

Modified from: Moher D, Liberati A, Tetzlaff J, Altman DG, The PRISMA Group (2009). Preferred Reporting Items for Systematic Reviews and Meta-Analyses: The PRISMA Statement. PLoS Med 6(7): e1000097

Title of the study, first author, year [ref]	Type of study	Study design	Population	N of patients, (age)	Experiments/mechanisms assessed	Outcome (primary and secondary)	Results	Effect [Relative risk (RR) or odds ratio (OR) + confidence interval (CI 95%)]
Antibiotic prescribing pattern in paediatric in patients with first time wheezing. **Patra, 2011 [1]**	Observational study	Comparison between clinical and laboratory parameters into two groups (who received antibiotics n=23 and who did not n=24)	All the children below two years of age admitted with first time wheezing (Department of pediatrics of tertiary care center at New Delhi)	47 (<2 years, mean age 5.8 months)	Subjects were divided into two groups- those who received antibiotics and those who did not<br><br>clinical and laboratory parameters were compared	Improved	No difference (p>0.5)	
						Deteriorated	No difference (p>0.1)	
						Mean Hospital stay	No difference (p>0.05)	
						Asymptomatic at follow up after 7 days	No difference (p=0.19)	
Wheeze in preschool age is associated with pulmonary bacterial infection and resolves after antibiotic therapy. **Schwerk, 2011 [2]**	Retrospective study	Comparison between children with severe recurrent or persistent wheezing (42) and control group (14) investigated by bronchoscopy and BAL	Children under the age of six years who underwent flexible bronchoscopy for severe recurrent or persistent wheeze between 2003 and 2010 at Hanover University Children's Hospital	42 (5-67 months)	Bronchoscopy and bronchoalveolar lavage (BAL) and modified ISAAC questionnaire	Neutrophilia and bacterial count	Increase in wheezing (22, 52% with bacterial count $>10^4$ cfu/ml and 20, 91% with neutrophilia) compared to the control group (3 (21%) with bacterial count $>10^4$ cfu/ml and 0 with neutrophilia) (p<0.005)	
						Improvement in patients (26) treated with antibiotics: amoxicillin or amoxicillin and clavulanic or cefuroxime or trimethoprim and sulfamethoxazole, for a median of six weeks (range 2-16 weeks)	24 out of 26 (92%) improved after therapy	
Azithromycin for episodes with asthma-like symptoms in young children aged 1-3 years: a randomized, double-blind, placebo-controlled trial.	Randomized, double-blind, placebo-controlled trial	Administration of azithromycin (79 episodes) or placebo (79 episodes) in	Children aged 1-3 years, who were diagnosed with recurrent asthma-like	72 (158 episodes) (1-3 years)	Each episode of troublesome lung symptoms was randomized individually to	Primary outcome: diary-verified duration of episodes of troublesome lung	63.3% reduction in the duration of the episode in the azithromycin (p<0.001)	63.3% (56.0-69.3)

**Stokholm, 2015 [3]**		children with *recurrent asthma-like symptoms* lasting at least 3 days	symptoms from the Copenhagen Prospective Studies on Asthma Childhood 2010 cohort		either azithromycin (3 day at 10mg/kg/die) or placebo	symptoms after initiation of treatment		
						Secondary outcomes		
						Time from treatment to the next episode	No difference (p=0.82)	Hazard ratio 0.95 (95% CI 0.65-1.40)
						The number of episodes that turned into severe exacerbations	No difference	
						Duration of SABA use after treatment	22% reduction in the number of days of SABA use in the azithromycin group (p=0.006)	22% (7.0-34.6)
Early administration of azithromycin and prevention of severe lower respiratory tract illnesses in preschool children with a history of such illnesses.<br>**Bacharier, 2016 [4]**	Randomized, double-blind, placebo-controlled trial	Participants with *recurrent, severe LRTIs* and minimal day-to-day impairment were randomly assigned to receive azithromycin (12 mg/kg/d for 5 days, n=307) or matching placebo (n=300) started early during each predefined RTI	607 children, aged 12-71 months enrolled between April 2011 and December 2014 across 9 academic US medical centers in the National Heart, Lung, and Blood Institute's Asthma Network	607 (12-71 months)	Screening cultures on deep oropharyngeal swab samples	<u>Primary outcome:</u> number of treated RTIs not progressing to severe LRTI	The azithromycin group experienced significantly lower risk of progression to severe LRTI (HR 0.64, p=0.04)	95% CI 0.41-0.98
						Secondary outcomes		
						Health care utilization	No difference	
						Time to second treated RTI	No difference	
						Development of azithromycin-resistant organism	No difference	
Treatment of preschool children presenting to the emergency department with wheeze with azithromycin: a placebo-controlled randomized trial.<br>**Mandhane, 2017 [5]**	Prospective, double-blinded, placebo-control randomized trial	Wheezy pre-school children were randomized to receive either five days of azithromycin (10mg/kg/die for day one and 5 mg/kg/die for four days; n=110) or placebo (n=112)	Wheezy pre-school children who presented to the Alberta Children's and Stollery Children's Hospitals emergency departments between January 2011 to May 2014	222<br>(12 to 60 months)	Parents completed a baseline questionnaire<br>Skin prick testing	<u>Primary:</u> the time (days) to respiratory symptoms resolution defined as three consecutive days of no symptoms using calendar diary	No difference (4 days for both groups, p=0.28)	
						<u>Secondary</u> outcomes		
						SABA use after treatment	No difference (4.5 days for azythromycin group vs 5 days for placebo group p=0.22)	

						Time to disease exacerbation	No difference (HR 0.91, p=0.65)	95% CI: 0.61 -1.36
Association between early antibiotic treatment and clinical outcomes in children hospitalized for asthma exacerbation.<br>**Okubo, 2020 [6]**	Retrospective cohort study	Compare clinical outcomes and resource utilization between children who received early antibiotic treatment , n=19866 during the first 2 days of hospitalization (macrolide 40.2%, cephalosporin 24.2%, penicillin 4150%) and those who did not	Pediatric inpatient aged between 3 months and 15 years with a diagnosis of asthma exacerbation between July 1, 2010, and March 31, 2018 (data collected from more than 1000 hospitals and covers up to 55% of all inpatient admission to acute-care hospitals in Japan.)	48743 (3 months-15 years)	Patients were divided into two group (those who received antibiotics during the first 2 days of hospitalization and those who did not or after 2 days)	Primary outcome: length of hospital stay	Increase in early treatment group (mean difference 0.21 days)	95% CI 0.17-0.25
						Secondary outcomes		
						Hospitalization cost	Increase in the early treatment group (mean difference 83.5 USD)	95% CI 62.9-104
						Risk of invasive or noninvasive mechanical ventilation	Increase in the early treatment group (RR 2.06)	95% CI 0.02-0.18
						Risk of 30-day readmission	No difference (RR 0.90)	95% CI 0.70-1.16
						Risk of probiotic use	Increase in the early treatment group (RR 2.01)	95% CI, 1-81-2.23)
The airway microbiota modulates effect of azithromycin treatment for episodes of recurrent asthma-like symptoms in preschool children.<br>**Thorsen, 2021 [7]**	Randomized, double-blind, placebo-controlled trial	To study the influence of the airway microbiota on the effect of azithromycin treatment (3 day at 10mg/kg/die) during acute episodes of asthma-like symptoms lasting at least 3 days	Children aged 1-3 years, who were diagnosed with recurrent asthma-like symptoms from the Copenhagen Prospective Studies on Asthma Childhood 2010 cohort	68 (139 episodes) (1-3 years)	Hypopharyngeal aspiration and 16S r RNA gene amplicon sequencing	Primary outcome: duration of episodes	Duration is associated with microbiota richness (7.5% increased duration per 10 additional operational taxonomic units -OTUs- p=0.025)	95% CI 1-14%
						Secondary outcome: evaluate effect modification by bacterial colonization	10% increased effect of azithromycin per 10 additional OTUs (p=0.04)	95% CI 0.4-18%)

References

1. Patra S, Singh V, Pemde HK, Chandra J. Antibiotic prescribing pattern in paediatric in patients with first time wheezing. Ital J Pediatr. 2011 Sep 5;37:40. doi: 10.1186/1824-7288-37-40. PMID: 21892931; PMCID: PMC3179436
2. Schwerk N, Brinkmann F, Soudah B, Kabesch M, Hansen G. Wheeze in preschool age is associated with pulmonary bacterial infection and resolves after antibiotic therapy. PLoS One. 2011;6(11):e27913. doi: 10.1371/journal.pone.0027913. Epub 2011 Nov 29. PMID: 22140482; PMCID: PMC3226624.
3. Stokholm J, Chawes BL, Vissing NH, Bjarnadóttir E, Pedersen TM, Vinding RK, Schoos AM, Wolsk HM, Thorsteinsdóttir S, Hallas HW, Arianto L, Schjørring S, Krogfelt KA, Fischer TK, Pipper CB, Bønnelykke K, Bisgaard H. Azithromycin for episodes with asthma-like symptoms in young children aged 1-3 years: a randomised, double-blind, placebo-controlled trial. Lancet Respir Med. 2016 Jan;4(1):19-26. doi: 10.1016/S2213-2600(15)00500-7. Epub 2015 Dec 17. PMID: 26704020; PMCID: PMC7164820.
4. Bacharier LB, Guilbert TW, Mauger DT, Boehmer S, Beigelman A, Fitzpatrick AM, Jackson DJ, Baxi SN, Benson M, Burnham CD, Cabana M, Castro M, Chmiel JF, Covar R, Daines M, Gaffin JM, Gentile DA, Holguin F, Israel E, Kelly HW, Lazarus SC, Lemanske RF Jr, Ly N, Meade K, Morgan W, Moy J, Olin T, Peters SP, Phipatanakul W, Pongracic JA, Raissy HH, Ross K, Sheehan WJ, Sorkness C, Szefler SJ, Teague WG, Thyne S, Martinez FD. Early Administration of Azithromycin and Prevention of Severe Lower Respiratory Tract Illnesses in Preschool Children With a History of Such Illnesses: A Randomized Clinical Trial. JAMA. 2015 Nov 17;314(19):2034-2044. doi: 10.1001/jama.2015.13896. Erratum in: JAMA. 2016 Jan 12;315(2):204. Erratum in: JAMA. 2016 Jan 26;315(4):419. PMID: 26575060; PMCID: PMC4757487.
5. Mandhane PJ, Paredes Zambrano de Silbernagel P, Aung YN, Williamson J, Lee BE, Spier S, Noseworthy M, Craig WR, Johnson DW. Treatment of preschool children presenting to the emergency department with wheeze with azithromycin: A placebo-controlled randomized trial. PLoS One. 2017 Aug 3;12(8):e0182411. doi: 10.1371/journal.pone.0182411. PMID: 28771627; PMCID: PMC5542589.
6. Okubo Y, Horimukai K, Michihata N, Morita K, Matsui H, Fushimi K, Yasunaga H. Association between early antibiotic treatment and clinical outcomes in children hospitalized for asthma exacerbation. J Allergy Clin Immunol. 2021 Jan;147(1):114-122.e14. doi: 10.1016/j.jaci.2020.05.030. Epub 2020 Jun 3. PMID: 32504615.

7. Thorsen J, Stokholm J, Rasmussen MA, Mortensen MS, Brejnrod AD, Hjelmsø M, Shah S, Chawes B, Bønnelykke K, Sørensen SJ, Bisgaard H. The Airway Microbiota Modulates Effect of Azithromycin Treatment for Episodes of Recurrent Asthma-like Symptoms in Preschool Children: A Randomized Clinical Trial. Am J Respir Crit Care Med. 2021 Jul 15;204(2):149-158. doi: 10.1164/rccm.202008-3226OC. PMID: 33730519.

PICO question 6. Is ipratropium bromide useful in exacerbation of preschool wheezing?

Patient or population: children with preschool wheezing

Setting: primary care/hospital

Intervention: ipratropium bromide use in exacerbation

Comparison: usual care with salbutamol

Search strategy:

(((child, preschool[MeSH Terms]) OR (toddler*)) AND ((respiratory sound[MeSH Terms]) OR (wheez*) OR (asthma[MeSH Terms:NoExp]))) AND (((ipratropium bromide[MeSH Terms]) OR (agents, anticholinergic[MeSH Terms]))) AND ((exacerbation*) OR (bronchospasm*)) AND ((english[Filter]) AND (2008:2021[pdat]))

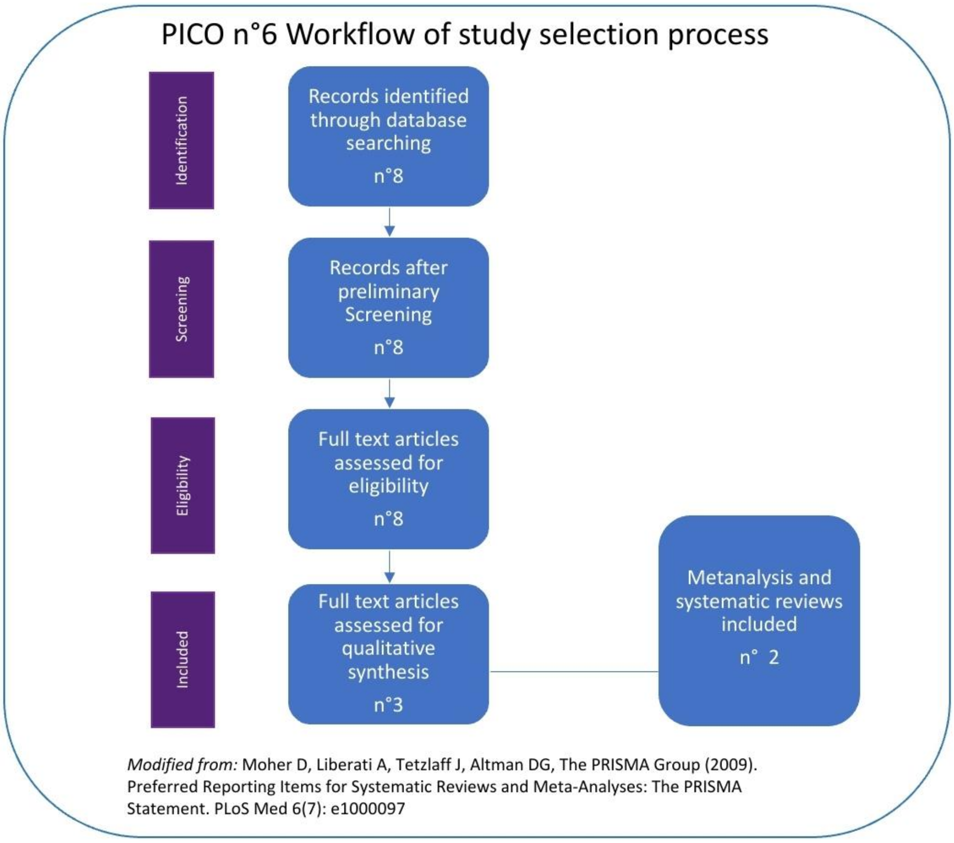

Title of the study, first author, year [ref]	Type of study	Study design	N of patients, (age)	Experiments/mechanisms assessed	Outcome (primary and secondary)	Results	Effect [Relative risk (RR) or odds ratio (OR) + confidence interval (CI 95%)]
Metered-dose inhaler ipratropium bromide in moderate acute asthma in children: A single-blinded randomised controlled trial.<br>**Wyatt E, 2015 [1]**	Randomized Controlled Trial	Patients with acute, moderate asthma were randomised to two groups, one receiving salbutamol, prednisolone and ipratropium bromide (IB), the other receiving only salbutamol and prednisolone	347 (2 – 15 years)	Prospective, single-blinded, randomised, controlled, equivalence trial.<br><br>Both groups received salbutamol by MDI (100 μg per actuation) three times at 20-min intervals (six actuations per dose if 2–5 years, 12 actuations per dose if 6–15 years, with five breaths between each actuation, consistent with usual practice) and oral prednisolone 1 mg/kg to a maximum of 50 mg. Additionally, the IB group received IB by MDI (21 μg per actuation), three times at 20-min intervals (four actuations per dose if 2–5 years, eight actuations per dose if 6–15 years, with five breaths between each actuation).	Risk of hospitalization<br><br>Difference in risk<br><br>Adverse effects	The absolute difference of +5.9% is not statistically equivalent but does not show a statistically significant decrease in admission rates when IB was given<br><br>Adverse effects were more prevalent in the IB group	70.1% vs 64.2%<br><br>+5.9% (−4.0% to 15.8%)<br><br>13.2% vs 4.6%<br>RR 2.86 (1.31-6.21).
Response to nebulized salbutamol versus combination with ipratropium bromide in children with acute severe asthma.<br>**Memon B, 2016 [2]**	Randomized Controlled Trial	Patients with acute asthma attack randomized into two equal groups of 100 children each.	200 (2 – 14 years)	Group A patients received 3 doses of nebulised salbutamol alone (0.03 ml/kg/dose) at 15-minute intervals and Group B received 3 similar doses of salbutamol along with ipratropium (250 ug/dose).<br>Efficacy was measured after 5minutes of the last dose by change in severity score from severe exacerbation (baseline) to low score.	Improvement in clinical score	In Group B, 93 (93%) children showed improvement in clinical score (<10 score) while it was 84 (84%) in Group A.<br>There was better response in clinical score in Group A than Group B, but it was not significant (p>0.05).	Group A: 84 (84 %) Clinical score before IB 8.4 ± 2.3 and after IB 4.9 ± 2.1 (p=0.001)<br><br>Group B: 93(93 %)<br>Clinical score before IB 8.6 ± 3.1 and after IB 4.4 ± 2.4 (p=0.007)
Inhaled anticholinergics and short-acting beta2-agonists versus short-acting beta2-agonists alone for children with acute asthma in hospital.<br>**Vézina K, 2014 [3]**	Systematic Review	Randomized trials (n=7) with paediatric patients hospitalised for an acute asthma exacerbation	472 (1 – 18 years)	All studies selected met the eligible criteria: randomised trials comparing the combination of inhaled or nebulised anticholinergics and short-acting β2-agonists versus short-acting β2-agonists alone in children one to 18 years of age hospitalised for an acute asthma exacerbation.<br>From the 7 RCT selected, only 4 had reported usable data	Duration of hospital stay.<br><br>Serious adverse events.<br><br><u>Secondary outcomes</u>:<br>Included admission and duration of stay in the intensive care unit (ICU)<br><br>Ventilation assistance<br><br>Time to short-acting β2-agonists spaced at four hours or longer	No differences<br><br>No differences<br><br>As a result of the similarity of trials, authors could not explore the influence of age, admission site, intensity of anticholinergic treatment and co-interventions on primary outcomes.<br><br>No statistically significant group difference was noted in	Mean difference (MD) -0.28 hours, 95% confidence interval (CI) -5.07 to 4.52

					Supplemental asthma therapy<br><br>Duration of supplemental oxygen<br><br>Change from baseline in asthma severity<br><br>Relapse after discharge<br><br>Adverse health effects<br><br>Withdrawals	other secondary outcomes, including the need for supplemental asthma therapy, time to short-acting β2-agonists spaced at four hours or longer, asthma clinical scores, lung function and overall withdrawals for any reason	
Combination of ipratropium bromide and salbutamol in children and adolescents with asthma: A meta-analysis.<br>**Xu H, 2021 [4]**	Meta-analysis	All studies selected (n=55) were randomized controlled trials with asthmatic paediatric patients or children <1 year old with wheezing who received IB+ salbutamol or salbutamol alone.	6396 (0 – 18 years)	All the studies selected met the eligible criteria: (1) randomized controlled trials (RCTs); (2) patients <18years; (3) physician-diagnosed asthma and children less than 1 year old with wheezing; (4) comparing IB+ salbutamol with salbutamol alone	Hospital admission<br><br>Hospital admission in patients with severe asthma exacerbation<br><br>Hospital admission in moderate-to-severe exacerbation<br><br>Adverse effects	IB + salbutamol significantly reduced the risk of hospital admission compared with salbutamol alone<br><br>Significantly reduced in IB+salbutamol group<br><br>Significantly reduced in IB+salbutamol group<br><br>No differences in the two groups	RR 0.79; 95% CI 0.66-0.95; p = 0.01; I2 = 40%<br><br>RR 0.73; 95% CI 0.60-0.88; p = 0.0009; I2 = 4%<br><br>RR 0.69; 95% CI 0.50-0.96; p = 0.03; I2 = 3%<br><br>RR 1.77; 95% CI 0.63-4.98

IB= Ipratropium bromide; RCT= Randomized Controlled Trial; RR= Risk Ratio; MDI= Metered-dose inhaler

References

1. Wyatt EL, Borland ML, Doyle SK, Geelhoed GC. Metered-dose inhaler ipratropium bromide in moderate acute asthma in children: A single-blinded randomised controlled trial. J Paediatr Child Health. 2015 Feb;51(2):192-8. doi: 10.1111/jpc.12692. Epub 2014 Jul 14. PMID: 25039574.
2. Memon BN, Parkash A, Ahmed Khan KM, Gowa MA, Bai C. Response to nebulized salbutamol versus combination with ipratropium bromide in children with acute severe asthma. J Pak Med Assoc. 2016 Mar;66(3):243-6. PMID: 26968269.
3. Vézina K, Chauhan BF, Ducharme FM. Inhaled anticholinergics and short-acting beta(2)-agonists versus short-acting beta2-agonists alone for children with acute asthma in hospital. Cochrane Database Syst Rev. 2014 Jul 31;(7):CD010283. doi: 10.1002/14651858.CD010283.pub2. PMID: 25080126.
4. Xu H, Tong L, Gao P, Hu Y, Wang H, Chen Z, Fang L. Combination of ipratropium bromide and salbutamol in children and adolescents with asthma: A meta-analysis. PLoS One. 2021 Feb 23;16(2):e0237620. doi: 10.1371/journal.pone.0237620. PMID: 33621253; PMCID: PMC7901745.

PICO question 7. Are leukotriene antagonists useful in exacerbation of preschool wheezing?

Patient or population: children with preschool wheezing

Setting: primary care/hospital

Intervention: systemic montelukast during exacerbation

Comparison: inhaled steroids or usual care with salbutamol

Search strategy:

((child, preschool [MeSH Terms]) OR (toddler*)) AND ((Respiratory Sounds[Mesh]) OR (wheez*) OR ("Asthma"[Mesh:NoExp])) AND (("Leukotriene Antagonists"[Mesh]) OR (montelukast)) AND (english[Filter]) AND (2008:2021[pdat])

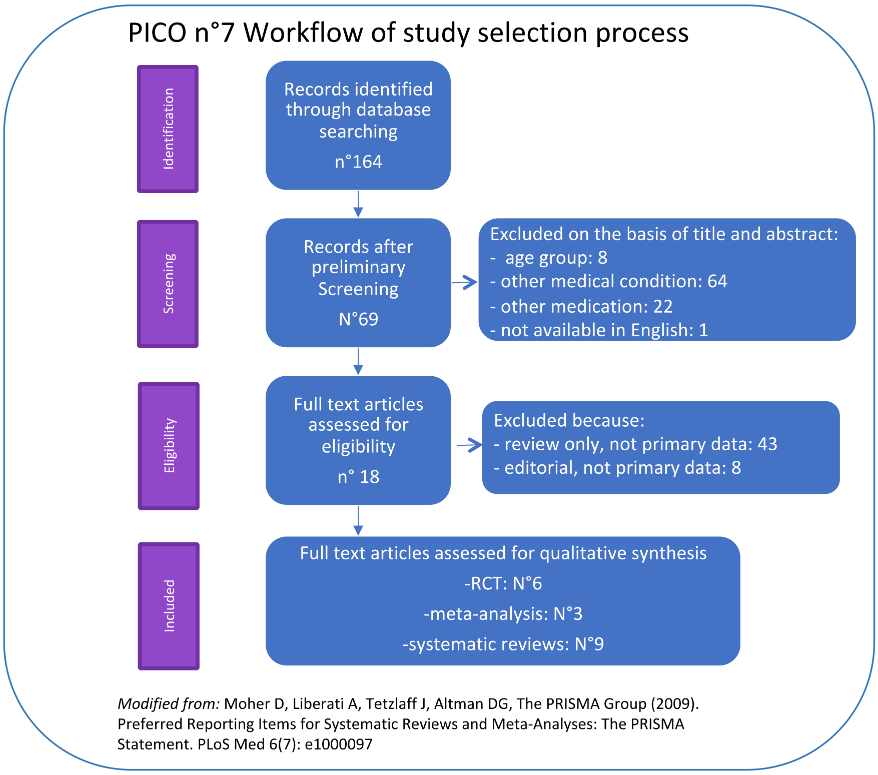

Title of the study, first author, year [ref]	Type of study	Study design	N of patients (age)	Experiments assessed	Outcomes	Results	Effect [Relative risk (RR) or odds ratio (OR) + confidence interval (CI 95%)]
Episodic use of an inhaled corticosteroid or leukotriene receptor antagonist in preschool children with moderate-to-severe intermittent wheezing.<br>**Bacharier LB, 2008 [1]**	Randomized double-blind, placebo-controlled trial	Administration of either budesonide inhalation (n 96), montelukast (n 95) or placebo (n 47) for 1 week in addition to albuterol in children with *moderate-to-severe intermittent wheezing*	238 (12-59 months)	7 days of inhaled budesonide (1 mg twice daily) vs montelukast 4 mg daily vs placebo	Primary outcome:<br>Proportion of episode-free days (EFDs) during 12 months	No difference among:<br>budesonide,<br>montelukast or<br>placebo	0.76 (0.70-0.81)<br>0.73 (0-66-0.79)<br>0.74 (0.65-0.81)
					Secondary outcomes:		
					Oral corticosteroid use	No difference (p = 0.15)	
					Health care use	No difference (p = 0.98)	
					Quality of life	No difference (p > 0.16)	
					Linear growth	No difference (p = 0.59)	
					Trouble breathing	Improved with budesonide (p = 0.003) and montelukast (p = 0.003)	Score AUC: 4.2 (3.1-5.2)<br>4.2 (3.1-5.3)
					Interference with activity scores	Improved with budesonide (p = 0.01) and montelukast (p = 0.001)	Score AUC: 4.8 (3.7-5.9)<br>4.3 (3.1-5,4)
					Mean total symptoms score	Improved with budesonide (p = 0.001) and montelukast (p = 0.003)	Score AUC:<br>5.8 (4.8-6.7)<br>5.4 (4.4-6.4)
Can montelukast shorten prednisolone therapy in children with mild to moderate acute asthma? A randomized controlled trial.<br>**Schuh S, 2009 [2]**	Randomized double-blind, double-dummy non-inferiority trial	Stabilization of outpatients with after adjustments for multiple comparisons. with montelukast (n 67) vs prednisolone (n 63)	130 (2-17 years)	Montelukast 4 mg (2-5 yrs), 5 mg (6-14 yrs), and 10 mg (15-17 yrs)/day for 5 days<br>vs 1 mg/kg/day prednisolone for 5 days	Primary outcome after adjustments for multiple comparisons.<br>Treatment failure occurring between discharge home and day 8, defined as an asthma-related unscheduled visit or hospitalization or treatment with systemic steroids.	Montelukast 22.4% vs prednisolone 7.9%<br><br>Overall treatment effect<br><br>(more frequent in children aged ≤3 yrs)	-14.5% (-26.5%--2.4%)<br><br>OR 3.95 (1.27-14.42)<br><br>OR 4.9 (1.66-15.22)
					Secondary outcomes		

					Need for additional medication	Montelukast 23.9% vs prednisolone 9.5% (p = 0.03).	
					Days free from symptoms<br><br>Daily need for albuterol	No difference (p = 0.75)<br><br>No difference (p = 0.85)	
Intermittent or daily montelukast versus placebo for episodic asthma in children. **Valovirta E, 2011 [3]**	Randomized multicenter, double-blind, double-dummy, parallel-group, placebo-controlled trial	Daily montelukast (n 589) vs intermittent montelukast (n 591) vs placebo (n 591) in *episodic asthma*	1771 (6 months-5 years)	Montelukast 4 mg daily vs montelukast 4 mg episode-driven vs placebo	Primary outcome<br>No. wheezing episodes culminating in acute attacks	No difference between placebo and daily (p = 0.510)<br>or intermittent montelukast (p = 0.884).	Rate reduction 5.3% (-11.4,19.6) vs -1.2% (-19.2,14.0)
					Secondary outcomes		
					Symptoms during the 12-day treatment episode	No difference between placebo and daily (p = 0.045) or intermittent montelukast (p = 0.062) after adjustments for multiple comparisons.	LS mean difference<br>-0.12 (-0.24, 0.00)<br>-0.11 (-0.23, 0.00)
					Number of asthma attacks	No difference between placebo and daily (p = 0.676) or intermittent montelukast (p = 0.802).	Rate reduction 3.3% (-13.1, 17.3)<br>-2.0% (-19.2, 12.7)
					Percentage of asthma-free days	No difference between placebo and daily (p = 0.074) or intermittent montelukast (p = 0.654).	LS mean difference<br>1.06 (-0.10, 2.23)<br>-0.27 (-1.43, 0.90)
					β-agonist use (times per day)	No difference between placebo and daily or intermittent montelukast after adjustments for multiple comparisons.	LS mean difference<br>-0.29 (-0.57, 0.00)<br>-0.31 (-0.59, -0.03)
Intermittent montelukast in children aged 10 months to 5 years with wheeze (WAIT trial): a multicentre, randomised, placebo-controlled trial **Nwokoro C, 2014 [4]**	Randomized multicenter, double-blind, parallel-group, placebo-controlled trial	Montelukast (n 669) vs placebo (n 677) during the *acute attack of wheezing*<br><br>Stratification according to ALOX5 promoter genotype (5/5, 5/x, x/x)	1358 (10 months-5 years)	Montelukast vs placebo	Primary outcome:<br>Difference in number of unscheduled medical attendances (USMA) for wheezing episodes	No difference (mean 2.0 [SD 2.6] vs 2.3 [2.7]; p=0.06).<br><br>USMA were reduced in children given montelukast with 5/5 genotype vs placebo (2.0 [2.7] vs 2.4 [3.0]; p=0.01), but not in those with 5/x+x/x genotype (2.0 [2.5] vs 2.0 [2.3]; p=0.79, pinteraction=0.08).	IRR 0.88 (0.77-1.01)<br><br>IRR 0.80 (0.68-0.95)<br><br>IRR 1.03 (0.83-1.29)

					Secondary outcomes		
					No. wheeze episodes	2.7 (2.9) vs 2.6 (3.0), p = 0.68	IRR 1.02 (0.91–1.16)
					Duration of wheeze episodes (days)	5.2 (4.0) vs 5.4 (3.8), p = 0.53	IRR 0.97 (0.89–1.06)
					Time to first USMA (days)	147 (50–365) vs 130 (38–), p = 0.09	HR 0.89 (0.78–1.02)
					Courses of rescue oral corticosteroids (number per child)	0.26 (0.7) vs 0.33 (0.9), p = 0.03	IRR 0.75 (0.58–0.98)
					Proportion of children receiving oral corticosteroids, (number, (%))	233 (34%) vs 227 (34%), p = 0.86	OR 0.98 (0.78-1.23)
Montelukast treatment of acute asthma exacerbations in children aged 2 to 5 years: A Randomized, Double-Blind, Placebo-Controlled Trial. **Wang X, 2018 [5]**	Randomized double-blind, placebo-controlled, parallel-group trial	Montelukast (n 60) vs placebo (n 60) during *acute asthma attack* (for a mean of 2.8 days)	120 (2-5 years)	Montelukast 4 mg daily vs placebo	Difference in PEF	No difference (p = 0.92 at day 2, p = 0.86 at day 3, p = 0.82 at day 4 and at discharge p = 0.84)	
					Difference in FEV_1	No difference in FEV_1 at discharge (p = 0.80)	
Effects of using montelukast during acute wheezing attack in hospitalized preschool children on the discharge rate and the clinical asthma score. **Demet Akbaş E, 2021 [6]**	Randomized double-blind, placebo-controlled, parallel-group trial	Montelukast (n. 50) vs placebo (n. 50) during *acute asthma attack* in a hospital setting	100 (6-72 months)	Montelukast 4 mg daily vs placebo until hospital discharge	Primary outcome<br>Difference between groups mean hospital length of stay in hours (LOS)	No difference LOS (median IQR): [68 (44–96) vs. 66 (47–99); p= 0.981].	
					Secondary outcomes		
					O2 saturation	No difference (p = 0.535)	
					Clinical asthma score	No difference (p = 0.708)	

Leukotriene receptor antagonists as maintenance and intermittent therapy for episodic viral wheeze in children. **Brodlie M, 2015 [7]**	Meta-analysis	Intermittent montelukast 4 mg vs placebo in *episodic viral wheeze*	343 (1-5 years)		No. of wheezing episodes requiring oral steroids	No effect	RR 0.85 (0.64-1.14)
Preventing exacerbations in preschoolers with recurrent wheeze: a meta-analysis. **Kaiser SV, 2016 [8]**	Meta-analysis	Intermittent montelukast vs intermittent ICS vs placebo	Preschoolers (age not known)		Intermittent montelukast vs intermittent ICS vs placebo	No effect	RR 0.82 (0.59-1.15)
A meta-analysis of montelukast for recurrent wheeze in preschool children. **Hussein HR, 2017 [9]**	Meta-analysis	Intermittent montelukast vs placebo	1334 vs 1288		Number of wheezing episodes		0.07 (-0.14-0.29)
			1413 vs 1320		Number of unscheduled medical attendance		-0.13 (-0.33-0.07)
			746 vs 703		Number of oral corticosteroids course		-0.06 (-0.15-0.02)

OR: odds ratio, EFDs:bepisode-free days, LS:least square , SD: standard deviation, IRR: incidence rate, HR; hazard ratio, USMA: unscheduled medical attendances, LOS: length of stay, ICS: inhaled corticosteroids, PEF: peak expiratory flow, FEV1: forced expiratory flow in 1 second.

References

1. Bacharier LB, Phillips BR, Zeiger RS, Szefler SJ, Martinez FD, Lemanske RF Jr, Sorkness CA, Bloomberg GR, Morgan WJ, Paul IM, Guilbert T, Krawiec M, Covar R, Larsen G, Mellon M, Moss MH, Chinchilli VM, Taussig LM, Strunk RC; CARE Network. Episodic use of an inhaled corticosteroid or leukotriene receptor antagonist in preschool children with moderate-to-severe intermittent wheezing. J Allergy Clin Immunol. 2008 Dec;122(6):1127-1135.e8. doi: 10.1016/j.jaci.2008.09.029. Epub 2008 Oct 30. PMID: 18973936; PMCID: PMC2753208.
2. Schuh S, Willan AR, Stephens D, Dick PT, Coates A. Can montelukast shorten prednisolone therapy in children with mild to moderate acute asthma? A randomized controlled trial. J Pediatr. 2009 Dec;155(6):795-800. doi: 10.1016/j.jpeds.2009.06.008. Epub 2009 Aug 4. PMID: 19656525.
3. Valovirta E, Boza ML, Robertson CF, Verbruggen N, Smugar SS, Nelsen LM, Knorr BA, Reiss TF, Philip G, Gurner DM. Intermittent or daily montelukast versus placebo for episodic asthma in children. Ann Allergy Asthma Immunol. 2011 Jun;106(6):518-26. doi: 10.1016/j.anai.2011.01.017. Epub 2011 Mar 4. PMID: 21624752.
4. Nwokoro C, Pandya H, Turner S, Eldridge S, Griffiths CJ, Vulliamy T, Price D, Sanak M, Holloway JW, Brugha R, Koh L, Dickson I, Rutterford C, Grigg J. Intermittent montelukast in children aged 10 months to 5 years with wheeze (WAIT trial): a multicentre, randomised, placebo-controlled trial. Lancet Respir Med. 2014 Oct;2(10):796-803. doi: 10.1016/S2213-2600(14)70186-9. Epub 2014 Sep 8. PMID: 25212745; PMCID: PMC4189104.
5. Wang X, Zhou J, Zhao X, Yi X. Montelukast Treatment of Acute Asthma Exacerbations in Children Aged 2 to 5 Years: A Randomized, Double-Blind, Placebo-Controlled Trial. Pediatr Emerg Care. 2018 Mar;34(3):160-164. doi: 10.1097/PEC.0000000000001184. PMID: 28590992.
6. Demet Akbaş E, Razi CH, Andıran N. Effects of using montelukast during acute wheezing attack in hospitalized preschool children on the discharge rate and the clinical asthma score. Pediatr Pulmonol. 2021 Jul;56(7):1931-1937. doi: 10.1002/ppul.25394. Epub 2021 Apr 12. PMID: 33844890.
7. Brodlie M, Gupta A, Rodriguez-Martinez CE, Castro-Rodriguez JA, Ducharme FM, McKean MC. Leukotriene receptor antagonists as maintenance and intermittent therapy for episodic viral wheeze in children. Cochrane Database Syst Rev. 2015 Oct

19;2015(10):CD008202. doi: 10.1002/14651858.CD008202.pub2. PMID: 26482324; PMCID: PMC6986470.

8. Kaiser SV, Huynh T, Bacharier LB, Rosenthal JL, Bakel LA, Parkin PC, Cabana MD. Preventing Exacerbations in Preschoolers With Recurrent Wheeze: A Meta-analysis. Pediatrics. 2016 Jun;137(6):e20154496. doi: 10.1542/peds.2015-4496. PMID: 27230765.
9. Hussein HR, Gupta A, Broughton S, Ruiz G, Brathwaite N, Bossley CJ. A meta-analysis of montelukast for recurrent wheeze in preschool children. Eur J Pediatr. 2017 Jul;176(7):963-969. doi: 10.1007/s00431-017-2936-6. Epub 2017 Jun 1. PMID: 28567533; PMCID: PMC5486554.

3) Controller therapy for the preschool child with wheezing

PICO question 8. When should controller medication be started?

Patient or population: children with preschool wheezing

Setting: primary care/hospital

Intervention: controller medication

Comparison: no treatment

Search strategy:

("Anti-Asthmatic Agents/therapeutic use"[Mesh] OR "Adrenal Cortex Hormones/therapeutic use"[Mesh] OR "steroids" OR" inhaled steroids" OR ("controller medication" OR "controller" OR "preventer" OR "maintenance" OR "daily") AND ("Respiratory Sounds"[Mesh] OR "wheeze" OR "wheezing") AND ("Child, Preschool"[Mesh] OR "toddler") AND (2008:2021[pdat])

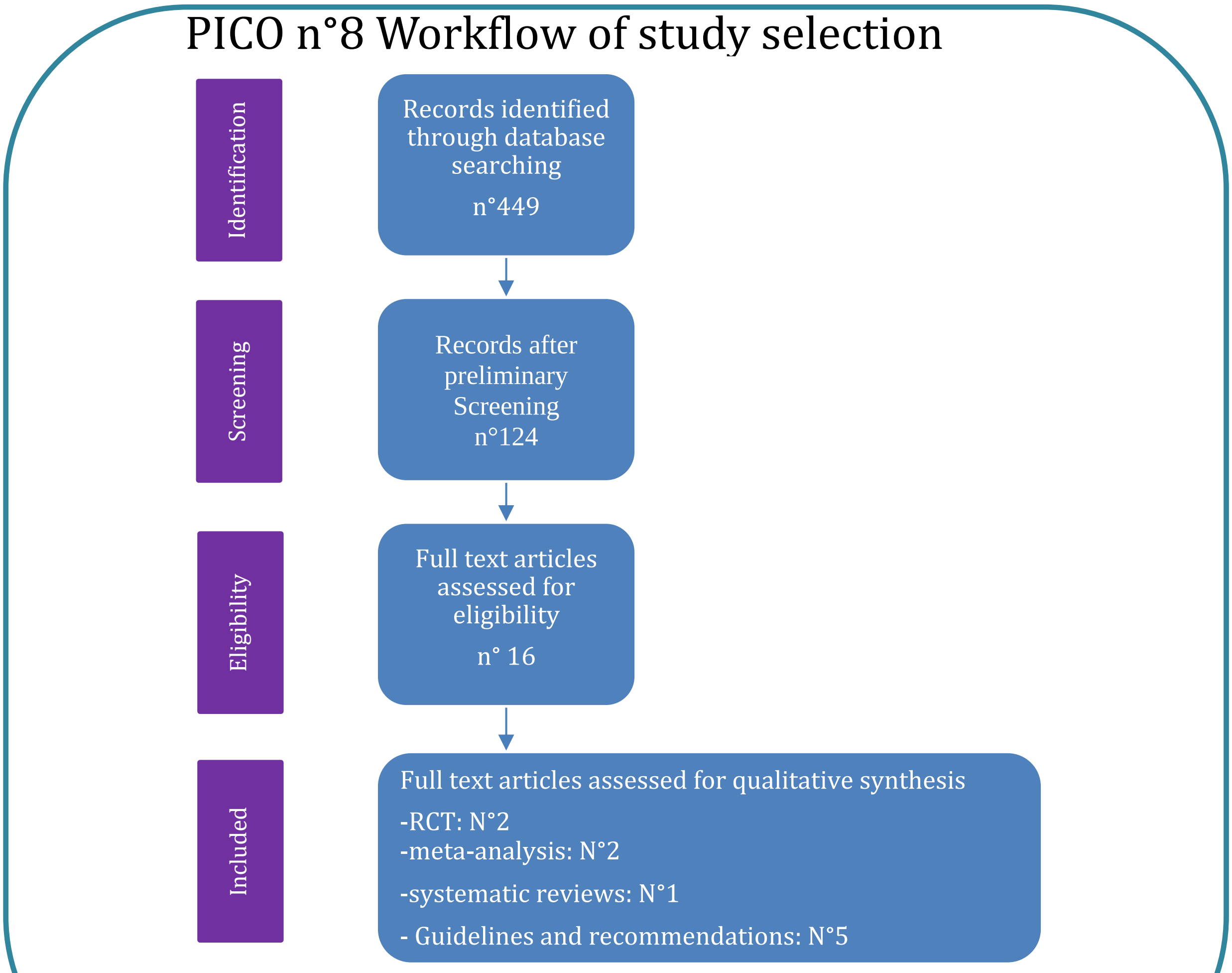

Modified from: Moher D, Liberati A, Tetzlaff J, Altman DG, The PRISMA Group (2009).
Preferred Reporting Items for Systematic Reviews and Meta-Analyses: The

Title of the study, first author, year [ref]	Type of study	Study design	N of patients, age	Experiments/mechanisms assessed	Outcomes (primary and secondary)	Results	Effect [Relative risk (RR) or odds ratio (OR) + confidence interval (CI 95%)]
Regular vs prn nebulized treatment in wheeze preschool children. **Papi A, 2009 [1]**	Double-blind, double-dummy RCT	Evaluation of the daily ICS effect in preschool children with frequent wheeze	N=276<br><br>Age: 1-4 years	Beclomethasone (400 mcg bid) + salbutamol prn (2500 mcg) (N= 110)<br>Vs<br>Placebo bid + beclomethasone/ salbutamol combination prn (800 mcg/1600 mcg) (N=110)<br>vs<br>Placebo bid + salbutamol prn (2500 mcg) (N=56) for a twelve weeks period	Primary outcome: symptom free days	Number of symptoms free days was higher in regular beclomethasone group (69.6±20.89) vs prn salbutamol group (61.0±24.83), but not vs prn combination (64.9±24.74)	Regular beclomethasone vs prn salbutamol p=0.034 (0.58-13.15)<br>Regular beclomethasone vs prn combination: p= 0.293 (–2.38; 7.62)<br>Prn combination vs prn salbutamol: p= 0.248 (–2.38 - 10.71)
					Secondary outcomes:		
					- Symptom score	Symptoms score improves, especially at night, in regular beclometasone group and in prn combination group compared to salbutamol.<br>Non statistically significant difference was found among regular beclomethasone and prn compination groups.	Regular beclomethasone vs prn salbutamol: p= 0.039; (–0.39- –0.01)<br>Prn combination vs prn salbutamol: p=0.014 (–0.43- –0.05)<br>Regular beclomethasone vs prn combination: p=0.626 (–0.12- 0.19)
					- Use of relief medication	No statistical significant difference was	Regular beclomethasone vs prn salbutamol:p =

						found among the three groups	0.156 (–0.18-0.03)<br>Regular beclomethasone vs prn combination:p = 0.995 (–0.08-0.09)<br>Prn combination vs prn salbutamol: p=0.151 (−0.18-0.03)
					- Time to first exacerbation	Time to first exacerbation was longer in regular beclomethasone group than in prn combination and prn salbutamol groups	p = 0.03
Efficacy of inhaled Corticosteroids in Infants and Preschoolers with recurrent wheezing and asthma: a systematic review with metaanalysis.<br>**Castro-Rodriguez JA, 2009 [2]**	Systematic review and meta-analysis	Compare the efficacy of inhaled corticosteroids in infants and preschoolers with recurrent wheezing or asthma compared to placebo	N=3592<br><br>Age: preschooler (1-5 years old)	Daily ICS vs placebo	Primary outcomes: numbers of exacerbations	Patient who received ICS has less exacerbations than those on placebo (18% vs 32,1%)	RR 0,59 (95% CI: 0,52-0,67; p=0.0001)<br><br>NNT: 7 [95% CI: 6 –9])
					Secondary outcomes		
					-Withdrawal rate due to wheezing/asthma exacerbations	ICS group showed a significant reduction in withdrawal rate compare to placebo	RR: 0.52 (0.43–0.63)<br>p = 0.0001
					-Changes in symptoms score	Symptom score significantly improved in ICS group compared to placebo	SMD: 0.93 (0.49–1.37)<br>p = 0.0001
					-Lung function (PEF)	Lung function significantly improved in ICS group compared to placebo	WMD: 13.8 (5.34–22.4)<br>p = 0.0001
					-Lung function (FEV_1)		WMD: 0.07 (0.05–0.09)

							p = 0.0001
					-Albuterol use	ICS group showed a significantly reduced need for albuterol use compare to placebo	SMD: 0.63 (0.30–0.63)<br>p = 0.0001
Ciclesonide in wheezy preschool children with a positive asthma predictive index or atopy.<br>**Brand LP, 2011 [3]**	RCT	Efficacy to start a continuative therapy with ICS compared to placebo, in patients with ongoing symptoms of recurrent wheezing and asthma positive predictive index or aeroallegen sensitization	N=1164<br>Age: 2-6 years	Daily ICS (Ciclesonide) 40 mcg vs<br>Daily ICS (Ciclesonide) 80 mcg vs<br>Daily ICS (Ciclesonide) 160 mcg<br>vs placebo for 24 weeks	Primary outcome: time to severe exacerbation (requiring oral steroids)	Exacerbation rate was lower in pooled ciclesonide groups (6.2%) than in placebo group (10.2%).	RR: 1.65 (95% CI: 1.04-2.63), (p = 0.030).
					Secondary outcomes:		
					-Percentage of wheeze-controlled days	No difference between groups.	
					- Symptom scores	No difference between groups.	
					-Use of rescue medication	No difference between groups.	
					- Lung function (in the subgroup with 4 to 6 years of age, N = 284) measured as FEV_1, PEF and FEF_{25-75}	FEV_1 significantly improved in ciclesonide 40 mcg group, compared to placebo;<br>FEF_{25-75} significantly improved in ciclesonide 40 mcg and 160 mcg groups, compared to placebo	$p < 0.05$
Preventing Exacerbations in Preschoolers with recurrent wheeze: a Meta-analysis<br>**Kaiser SV, 2016 [4]**	Meta-analysis	Synthesize the effects of daily inhaled corticosteroids (ICS) in preventing severe exacerbations among preschool children with recurrent wheeze	N=3278<br><br>Age: ≤6 years	Daily ICS vs placebo (n: 3278)<br><br>daily ICS vs placebo,<br>intermittent ICS vs placebo,<br>daily ICS vs intermittent ICS,<br>ICS vs montelukast	Primary outcome: number of exacerbations requiring oral steroids	There are less exacerbations with daily medium-dose ICS compared to placebo<br>(15 studies, N = 3278)	RR 0.70 (95% CI 0.61–0.79), NNT = 9

						Subgroup of children with persistent asthma has less exacerbations with daily ICS compared with placebo.<br>(8 studies, N = 2505)	RR 0,56 (95% CI: 0,46-0,70), NNT:11
						There are less exacerbation with daily ICS compared with montelukast (1 study, N = 202)	RR 0.59; (95% CI: 0.38–0.92)
						In the subgroup with intermittent asthma or viral-induce wheezing, preemptive high-dose intermittent ICS reduce exacerbation rate compared with placebo (5 studies, N = 422).	RR 0.65 (95% CI: 0.51–0.81), NNT = 6

References

1. Papi A, Nicolini G, Baraldi E, Boner AL, Cutrera R, Rossi GA, Fabbri LM; BEclomethasone and Salbutamol Treatment (BEST) for Children Study Group. Regular vs prn nebulized treatment in wheeze preschool children. Allergy. 2009 Oct;64(10):1463-1471. doi: 10.1111/j.1398-9995.2009.02134.x. PMID: 19772514.
2. Castro-Rodriguez JA, Rodrigo GJ. Efficacy of inhaled corticosteroids in infants and preschoolers with recurrent wheezing and asthma: a systematic review with meta-analysis. Pediatrics. 2009 Mar;123(3):e519-25. doi: 10.1542/peds.2008-2867. PMID: 19254986.
3. Brand PL, Luz García-García M, Morison A, Vermeulen JH, Weber HC. Ciclesonide in wheezy preschool children with a positive asthma predictive index or atopy. Respir Med. 2011 Nov;105(11):1588-95. doi: 10.1016/j.rmed.2011.07.017. Epub 2011 Aug 11. PMID: 21839625.
4. Kaiser SV, Huynh T, Bacharier LB, Rosenthal JL, Bakel LA, Parkin PC, Cabana MD. Preventing Exacerbations in Preschoolers With Recurrent Wheeze: A Meta-analysis. Pediatrics. 2016 Jun;137(6):e20154496. doi: 10.1542/peds.2015-4496. PMID: 27230765.

PICO question 9. In children with preschool wheezing, are inhaled steroids more effective (and/or safer) than leukotriene antagonists?

Patient or population: children with preschool wheezing

Setting: from primary to tertiary care

Intervention: continuous inhaled steroids

Comparison: leukotriene antagonists

Search strategy

((child, preschool[MeSH Terms]) OR (toddler*)) AND ((Respiratory Sounds[Mesh]) OR (wheez*) OR ("Asthma"[Mesh:NoExp])) AND ((("Administration, Inhalation"[Mesh]) OR (Inhaled)) AND ((ICS) OR (Adrenal Cortex Hormones[Mesh]) OR (*steroid*)) OR (”Leukotriene Antagonists"[Mesh]) OR (montelukast)) AND (english[Filter]) AND (2008:2021[pdat])

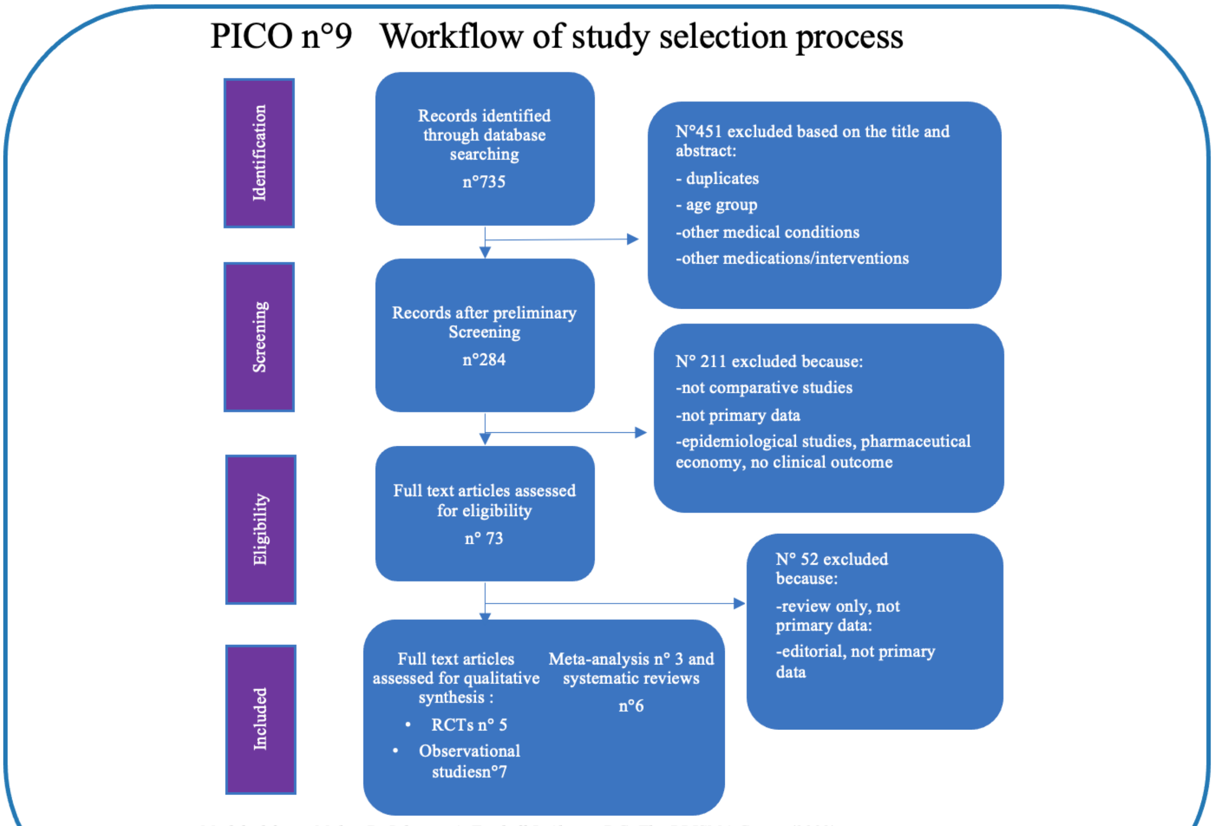

Modified from: Moher D, Liberati A, Tetzlaff J, Altman DG, The PRISMA Group (2009). Preferred Reporting Items for Systematic Reviews and Meta-Analyses: The PRISMA Statement. PLoS Med 6(7): e1000097

In Table 1 we report randomized controlled trials and observational studies and in Table 2 systematic reviews or metanalysis

Title of the study, first author, year [ref]	Type of study	Study design	Population	N of patients (age)	Experiments assessed	Outcomes	Results (p value)	Effect [Relative risk (RR) or odds ratio (OR) + confidence interval (CI 95%)]
Fluticasone or montelukast for preschool children with asthma-like symptoms: Randomized controlled trial.<br>**Kooi, 2008 [1]**	Multicenter randomized placebo controlled double-blind double-dummy trial	3 groups over 3 months-period: fluticasone propionate (FP) 100 mcg twice/die (n 25) vs montelukast 4 mg (n 18) vs Placebo (n 20)	Netherlands - children with asthma-like symptoms	63 (2-6 years)	Assessment of: daily symptom score (wheeze, cough, shortness of breath); rescue medication free days, blood eosinophils and lung function (interrupter technique, forced oscillation technique)	<u>Primary outcome:</u><br>-daily symptom score (wheeze, cough, shortness of breath) recorded by caregivers in diary record cards<br><br><u>Secondary outcomes:</u><br>-rescue medication free days<br><br>-blood eosinophils<br><br>-lung function (interrupter technique, forced oscillation technique)	Symptoms improved in all 3 groups (FP p<0.001; LTRA 0.025; placebo 0.026) with a significant difference in FP vs placebo (p 0.021)<br><br>significant decrease for rescue medication free days in each group (FP p 0.015; LTRA 0.002; placebo 0.013) no difference between groups<br><br>-LTRA group showed a reduction in circulating eosinophils (p 0.008) with a significant difference of LTRA vs placebo (p 0.045)<br><br>-no difference in lung function in the three groups except for frequency dependence (measured by forced oscillation technique) which showed an improvement in FP group compared to LTRA (p 0.048)	
Adequate levels of adherence with controller medication is associated with increased use of rescue medication in asthmatic children.<br>**Elkout, 2012 [2]**	Retrospective observational study	4 groups over 1 year period: inhaled corticosteroids (ICS) only (n 2297) vs leukotriene receptors antagonists (LTRA) (n 394) vs long acting beta2 agonists (LABA) + ICS separate inhalers (n	UK - children who were issued a new prescription for asthma controller medication	3172 (0-18 years)	Measures of adherence assessed using medication possession ratio (MPR) and level of asthma control assessed by identifying both the number of short-acting beta2 agonists (SABA)	<u>Outcomes:</u><br>-Medication possession ratio (MPR)<br><br>- number of short-acting beta2 agonists (SABA)	-MPR was poor in all groups (15-39% adequate MPR)<br>Grater MPR for LTRA (39%, p<0.05); undersupply was greater in ICS only (p<0.001)<br><br>-Adequate MPR is associated with greater use of rescue medication (>6 SABA canisters/year):<br>-ICS only p<0.001;<br>-LTRA p 0.004	-OR 1.89 (95% CI 1.35-2.48)

		481) vs LABA +ICS fixed dose (n 219)			inhalers and/or courses of oral corticosteroids prescribed over 1 year	inhalers/year (<6 canisters/year) And/or courses of oral corticosteroids prescribed over 1 year	-LABA/ICS p 0.001	- OR 2.11 (95% CI 1.27-3.49)<br>- OR 2.85 (95% CI 1.62-5.02)
Clinical effectiveness of inhaled corticosteroids versus montelukast in children with asthma: prescription patterns and patient adherence as key factors.<br>**Ducharme, 2012 [3]**	Retrospective cohort study	2 groups of children with mild-moderate asthma: ICS (n 169) vs LTRA monotherapy (n 58)	Canada - Children with mild-moderate asthma presented to Asthma center between 2000-2007	227 (2-17 years)	Rescue oral corticosteroids, prescription duration and dispensing, acute-care visits, hospital admissions, beta2 agonists use were assessed and adherence patterns were analyzed	Primary outcome:<br>-rate of rescue oral corticosteroids dispensed/person/year by pharmacies<br><br>Secondary outcomes:<br>-acute-care visits for asthma<br>-hospital admissions for asthma<br>-weekly use of >4doses of SABA<br><br>Adherence patterns<br>- The proportion of prescribed days covered (PPDC), a marker of patient compliance, was calculated as the number of days for which the drug was dispensed, divided by the number of days for which it was prescribed. The	No group differences in oral corticosteroids<br><br>No group differences in acute-care visits.<br>- ICS-treated children experienced more hospital admissions and needed more frequently SABA ≥4 doses/week<br><br>Physician prescriptions covered 62% of the follow-up period for ICS compared to 97% for LTRA<br>-In pharmacies, patients claimed 51 vs. 74% of prescribed ICS and montelukast, respectively.<br>-Consequently, dispensed ICS and montelukast covered 24% and 38% of follow-up period	RR = 1.10 (95%CI 0.66-1.84)<br><br>RR = 1.79 (95% CI 0.96-3.34)<br>RR = 3.63 (95% CI 1.20-11.03)<br><br>RR = 2.54 (95%CI 1.23 -5.23)<br><br>Mean group difference MGD= −17% (95% CI: −28%, −7%).<br><br>-MGD=−12% (95%CI −20%, −4%)<br><br>-MGD=−14% (95%CI −22%, −6%)

						proportion of days covered (PDC), indicative of drug possession, was computed as the cumulative number of days for which the prescribed drug was dispensed divided by days of follow-up		
Budesonide inhalation suspension versus montelukast in children aged 2 to 4 years with mild persistent asthma**.**<br>**Szefler, 2013 [4]**	Randomized, open label, active controlled multicenter study	2 groups over 52 weeks period: budesonide (BIS) 500 mcg/die (n 105) vs Montelukast 4-5 mg/die (n 97)	North America - children with mild asthma	202 (2-4 years)	The primary efficacy variable was time to first additional asthma medication for mild or severe asthma exacerbation (either morning step-up therapy with BIS or oral corticosteroids). Secondary efficacy variables times to the first mild and severe asthma exacerbations and rates of occurrence of mild and severe exacerbations (number/patient/ year).<br><br>Global assessments: Symptom scores,rescue medication use, and peak expiratory flow rates (PEF) were recorded daily by	Primary outcome:<br>Time to first asthma additional medication<br>Secondary outcomes:<br>-time to first mild/severe asthma exacerbation/patients/year.<br>-rate of mild/sever exacerbations/year<br><br>Global assessments:<br>-diary related variables (symptom score, PEF, rescue medication use)<br>-physician reported outcomes	no significant difference (p 0.128)<br><br>no difference<br><br>lower rate of mild or severe exacerbations/year for BIS over LTRA (p 0.003) with lower percentage of patients requiring corticosteroids for BIS group at week 12 (p 0.044), week 26 (p 0.029) and week 56 0.022)<br><br>no difference<br><br>physician-reported improvement in asthma symptoms at 12 weeks for BIS group over LTRA (p 0.021)	

					caregivers in the electronic diary. Patient-reported outcomes and global assessments were evaluated with the Child Health Questionnaire Parent Form-50 (CHQ-PF50), the Children's Health Survey for Asthma, the Pediatric Asthma Caregiver's Quality of Life Questionnaire and the Global Physician and Caregiver Assessments. Safety was evaluated by recording adverse events.	-patients reported outcomes<br><br>-safety assessment	better caregiver-reported ability to manage patient's symptoms at the end of the treatment for BIS group (p 0.034)<br><br>both medications were well tolerated	
Demographic predictors of leukotriene antagonist monotherapy among children with persistent asthma.<br>**Wu, 2014 [5]**	Cross-sectional study	2 groups of children with persistent asthma in the period 2007-2009: LTRA (n 2508) vs ICS (n 14346)	North Carolina USA	16849 (2-18 years)	Multivariate logistic regression modeling to compare the outcome of LTRA with the primary predictor of age and covariates of race, sex, rurality and disease severity; Negative binomial regression to compare outcomes of albuterol and oral steroid claims, outpatients and emergency department visits and hospitalizations	Primary outcome:<br>-Bivariate analysis with demographic variables (age sex and race) and county of residence (urban or rural) as a proxy for access to care.<br><br>Secondary outcome:<br>-healthcare utilization<br><br>-Albuterol use and oral steroid claims	Age, race, rurality were associated with LTRA prescription (p<0.01):<br>-5-13y relative to < 5y<br><br>-Caucasians relative to African Americans<br><br>-rural children relative to urban<br><br>No difference in emergency department visits or admissions<br><br>-Albuterol, oral steroids and outpatient visits were lower in LTRA group (p<0,01)	-OR 1.46 (95%CI 1.30-1.64)<br>-OR 1.40 (95% CI 1.27-1.53)<br><br>-OR 1.18 (95% CI 1.08-1.3)

					with predictors of LTRA vs ICS.			
Use of leukotriene receptor antagonists are associated with a similar risk of asthma exacerbations as inhaled corticosteroids. **Wu, 2014. [6]**	Retrospective cohort study	3 groups over 12 month period: ICS (n 17527) vs LTRA (7153) vs ICS/LABA combination (n 1511)	USA- children with asthma in the Population-Based Effectiveness in Asthma and Lung Diseases Network (PEAL)	26191 patients (15,567 health plan subjects and 10,624 TennCare Medicaid subjects with uncontrolled asthma) (4-17 years)	Cox regression analyses by adjusting for baseline covariates, adherence by using proportion of days covered, and high-dimensional propensity scores were conducted. The main outcome measurements were emergency department visits, hospitalizations, or oral corticosteroid use.	<u>Main outcome measures:</u><br>-emergency department visits<br>-hospitalizations<br><br>-oral corticosteroid use<br><br>-composite exacerbations oucome<br><br>(patients were distinguished in those with asthma only and those with asthma and allergic rhinitis)<br><br><u>Other assessments:</u><br>-to assess adherence to the controller medication, number of refills after initial fill were examined	-For subjects with allergic rhinitis, the subjects in TennCare Medicaid treated with LTRAs were less likely to experience ED visits compared with the subjects treated with ICS.<br>-For all other groups, the subjects treated with LTRA or ICS-LABA were just as likely to experience ED visits or hospitalizations, or need oral corticosteroids as the subjects treated with ICS.<br>-Risks of asthma-related exacerbations did not differ between children who initiated LTRA and ICS.<br><br>Overall adherence to controller medications was low, with no more than 50% of the subjects refilling the medication after the initial fill.	HR 0.44 (95% CI 0.21-0.93)

Fluticasone or montelukast in preschool wheeze: a randomized controlled trial.<br>**Krawiec, 2015 [7]**	Open-label randomized trial	2 groups over 12 weeks period: Montelukast 4 mg (n 23) vs Fluticasone inhaled suspension (FIS) (n 23) vs placebo (n 24)	Poland - Children with 1 to 3 episodes of pre-school wheeze admitted to Hospital between November 2007 and August 2010	70 (3-36 months)	The study aimed to evaluate the effect of anti-inflammatory treatment on frequency and severity of preschool wheeze episodes. The outcome measures were the number of PWEs, the number of hospitalizations due to wheezing, and the severity of respiratory symptoms.<br>Study clinicians obtained the medical history from parents or caregivers (included questions about history of allergic disease in the immediate family passive smoke exposure9	Primary outcomes:<br>-number of wheezing episodes/year<br>-percentage of children who had wheezing episodes/year<br>Secondary outcomes:<br>-number of wheezing episodes in the first 12 weeks<br>- percentage of children who had wheezing episodes in the first 12 weeks<br>-number of hospitalizations	- no significant differences<br>- no significant differences<br>- no significant differences<br>- no significant differences<br>- no significant differences<br>Other assessments: tobacco-exposed children treated with fluticasone had significantly fewer PWEs (P = .01).	
Individualized therapy for persistent asthma in young children.<br>**Fitzpatrick, 2016 [8]**	Multicenter randomized double-blind double-dummy clinical trial	Participants completed a 2- to 8-week run-in period followed by 3 crossover periods with daily inhaled corticosteroids (ICSs), daily leukotriene receptor antagonists, and as-needed ICS treatment coadministered with albuterol.	USA - children with asthma necessitating treatment with daily controller (Step 2) therapy of the Individualized Therapy for Asthma in Toddlers study	230 evaluable (12- 59 months)	The primary analysis involved 2 stages: determination of differential response and assessment of whether 3 prespecified features (aeroallergen sensitization, previous exacerbations, and sex) predicted a differential response.	Primary outcome:<br>-Differential response to 3 therapies<br>Secondary outcomes:<br>-annualized number of asthma control days<br>-exacerbation rates	Seventy-four percent (170/230) of children with analyzable data had a differential response to the 3 treatment strategies. Within differential responders, the probability of best response was highest for a daily ICS ($p<0.0001$).<br>Daily ICS use was associated with more asthma control days and fewer exacerbations compared with the other treatments($p<0.0001$).	

					Adverse events were recorded as safety assessment	-rescue albuterol inhalations<br><br><u>Additional assessment:</u><br>-assessment of whether 3 prespecified features (aeroallergen sensitization, previous exacerbations, and sex) predicted a differential response<br>-Adverse events	no difference<br><br>Best response was predicted by aeroallergen sensitization (p 0.0036) but not exacerbation history or sex. The probability of best response to daily ICS was further increased in children with both aeroallergen sensitization (p 0.0374) and blood eosinophil counts of 300/μL or greater (p 0.0071).<br><br>No difference	
Efficacy of montelukast sodium chewable tablets combined with inhaled budesonide in treating pediatric asthma and its effect on inflammatory factors.<br>**Zhang, 2019 [9]**	Randomized controlled trial	3 groups of asthmatic children over a 4 week period: BIS (n 45) vs LTRA (n 45) vs BIS+LTRA (n 45)	China - asthmatic children	135 (3-12 years)	Clinical symptoms, lung function, inflammatory factors and immune related indices of patients in each group were observed and recorded.	Primary outcome:<br>-levels of hypersensitive C-reactive protein (hs-CRP), IgE, IL-4, IL-8, TNF-α, CD3/CD4/CD8 detected by flow cytometry<br><br><u>Secondary outcomes:</u><br>Disappearance time of wheezes, dyspnea and asthma, discharge time of children	TNF-α, IL-4, IL-8 and hs-CRP were significantly lower than before treatment, and the combined group was significantly lower than the single-drug group in the same period (all $p < 0.05$). The number of CD4+ and CD3+ cells in the combined group was significantly higher than that in the single-drug group, while the number of CD8 + cells and the expression level of IgE were significantly lower (all $p < 0.05$).<br><br>Times to disappearance of wheezes, dyspnea, asthma and hospital stay in the BIS+LTRA group were significantly shorter than those in the single-drug group (all $p < 0.001$). Six months after treatment, the incidence of asthma in the combined group was significantly lower than that in the single-drug group (both $p < 0.05$).	

						-Spirometry measures (Forced vital capacity (FVC), forced expiratory volume in the first second (FEV1, FEV1/FVC, peak expiratory flow (PEF))<br><br>-Adverse reaction	FVC, FEV1, PEF were significantly higher than those before treatment, and in the combined group value were significantly higher than in the single-drug group in the same period (all $p < 0.001$)<br><br>No difference in the incidence of adverse reactions between the groups during treatment ($p > 0.05$). Six months after treatment, the incidence of asthma in the combined group was significantly lower than that in the single-drug group (both $p < 0.05$).	
Healthcare utilization in infants and toddlers with asthma-like symptoms. **Levine, 2019 [10]**	Historical prospective study	2 groups over 3 months and 12 months period of controller therapy: ICS (Fluticasone propionate, Beclometasone dipropionate, Budesonide) (20316) with or without LTRA vs LTRA (Montelukast 4 mg) alone (n 5792) , no therapy (236792)	Israel-children born 2005-2012 who before 3 years of age had >3 episodes of asthma-like symptoms and/or >2 bronchodilator purchases within a year	262.900 patients with >3 episodes/year of asthma-like symptoms: 26108 patients receiving controller therapy - n°20316 on ICS, n° 5792 on LTRA (3-12 years)	Primary assessment was healthcare utilization after 3 and 12 months of controller therapy (respiratory-related doctor visits). Linear and categorical regression analysis measured overall effectiveness of therapy	<u>Primary outcome:</u><br>-Number of respiratory related doctor visits at 3 and 12 months<br><br><u>Secondary outcomes:</u><br>-Number of respiratory related hospital admissions at 3 and 12 months<br><br>-Pneumonia diagnosis at 3 and 12 months<br><br>-Chest x ray at 3 and 12 months	-Doctor visits: both groups showed a decrese<br>-ICS ± LTRA ($p<0.001$) at 3 months<br>-LTRA ($p<0.001$) at 3 months<br>-ICS ± LTRA ($p<0.001$) at 12 months<br>-LTRA alone ($p<0.001$) at 12 months<br><br>-Hospital admissions<br>ICS ± LTRA ($p<0.001$) at 3 months<br>LTRA alone (p<0,001) at 3 months<br>ICS ± LTRA (p 0.37) at 12 months<br>LTRA alone (p<0,001) at 12 months<br><br>-Pneumonia diagnosis<br>ICS ± LTRA ($p<0.001$) at 3 months<br>LTRA alone (p 0.004) at 3 months<br>ICS ± LTRA ($p<0.001$) at 12 months<br>LTRA alone (p 0.058) at 12 months<br><br>-Chest x-rays:<br>ICS ± LTRA ($p<0.001$) at 3 months<br>LTRA alone (p<0,001) at 3 months	-OR 0.13 (95%CI 0.11-0.15)<br>-OR 0.54 (95%CI 0.45-0.65)<br>-OR 0.23 (95%CI 0.16-0.32)<br>-OR 0.42 (95%CI 0.22-0.76)<br><br>-OR 0.68 (95%CI 0.63-0.72)<br>-OR 0.68 (95%CI 0.58-0.79)<br>-OR 0.96 (95%CI 0.81-1.14)<br>-OR 2.16 (95%CI 1.53-3.08)<br><br>-OR 0.69 (95%CI 0.65-0.73)<br>-OR 0.81 (95%CI 0.70-0.94)<br>-OR 0.42 (95%CI 0.37-0.48)<br>-OR 0.96 (95%CI 0.70-1.32)<br><br>-OR 0.41 (95%CI 0.39-0.43)<br>-OR 0.35 (95%CI 0.31-0.39)

						-Use of bronchodilators at 3 and 12 months<br><br>-Use of oral steroids at 3 and 12 months	ICS ± LTRA (p<0.001) at 12 months<br>LTRA alone (p<0,001) at 12 months<br><br>-Bronchodilators:<br>ICS ± LTRA (p<0.001) at 3 months<br>LTRA alone (p<0,001) at 3 months<br>ICS ± LTRA (p<0.001) at 12 months<br>LTRA alone (p<0,001) at 12 months<br><br>-Systemic steroids:<br>ICS ± LTRA (p<0.001) at 3 months<br>LTRA alone (p<0,001) at 3 months<br>ICS ± LTRA (p<0.001) at 12 months<br>LTRA alone (p<0,001) at 12 months	-OR 0.06 (95%CI 0.05-0.08)<br>-OR 0.11 (95%CI 0.07-0.15)<br><br>-OR 0.26 (95%CI 0.25-0.28)<br>-OR 0.48 (95%CI 0.44-0.54)<br>-OR 0.22 (95%CI 0.19-0.25)<br>-OR 0.36 (95%CI 0.27-0.49)<br><br>-OR 0.50 (95%CI 0.48-0.52)<br>-OR 0.61 (95%CI 0.56-0.66)<br>-OR 0.46 (95%CI 0.42-0.51)<br>-OR 0.85 (95%CI 0.69-1.04)
Comparative effectiveness of budesonide inhalation suspension and montelukast in children with mild asthma in Korea.<br>**Shin, 2020 [11]**	Retrospective observational cohort study	2 groups: BIS monotherapy (n 1221) vs LTRA (n 4831) over 1 year period	Korea - children diagnosed with mild persistent asthma who initiated BIS or LTRA during 2015	26,052: for unmatched -n = 1,221 BIS; n = 24, 831 LTRA and 2,290 for matched populations -n = 1,145 per cohort. (2-17 years)	Adherence, persistency, asthma control, asthma-related health-care resource utilization, and costs were evaluated using unadjusted descriptive statistics and propensity score-matched regression analyses.	<u>Outcomes:</u><br>-adherence (measured by proportion of days covered)<br>-treatment persistency (Time to first loss of persistency)<br>-asthma control<br><br>-health resource utilization<br><br>-costs	-low for both cohorts but significantly higher for LTRA versus BIS (13.8% vs. 4.5%; p <0.001).<br>-longer for LTRA vs BIS (82.3 vs. 78.4 days, respectively; p < 0.001).<br><br>-Mean number of post-index asthma-related office visits was 6.6 for BIS versus 8.3 for LTRA (p < 0.001).<br>-A greater proportion of patients in the BIS cohort had an asthma exacerbation-related office visit than the LTRA cohort (78.3% vs. 56.1%; p < 0.001).<br>-Asthma-related total health-care costs were higher with LTRA versus BIS (p < 0.001), likely driven by higher pharmaceutical costs associated with LTRA p < 0.001).	-RR 2.936<br><br>-HR 0.838

Treatment of pediatric mild persistent asthma with low-dose budesonide inhalation suspension vs. montelukast in China. **Chen, 2021 [12]**	Retrospective cross-sectional observational study	2 groups over 12 months period: Budesonide inhalation suspension (BIS) 500 mcg/die vs Montelukast 4 mg/die (LTRA)	China-patients with mild persistent asthma	393 patients - 153 on BIS+ 240 on Montelukast (2-14 years)	Questionnaire based analysis - indicators of asthma control: Asthma Control Test (ACT)/Children-ACT e asthma related medical costs	Outcomes assessed:<br>-experienced asthma symptoms in past 4 weeks<br>-asthma symptoms more than twice/week in past 4 weeks<br>-activity limitation due to asthma<br>-night awakening or night coughing due to asthma<br>-reliever medication needed more than twice/week<br>-ACT<19<br>-exercise quality in the past 12 months<br>-asthma related medical costs	- no difference<br>-lower percentage of symptoms twice a week for LTRA (p 0.021)<br>- no difference<br>- Lower night coughing for LTRA (p 0.022)<br>- Lower required reliever medication for LTRA (p <0.001)<br>- lower percentage of ACT/C-ACT<19 for LTRA (p 0.021)<br>- better exercise tolerance for LTRA (p 0.015)<br>- lower asthma related medical costs for LTRA (p<0.001)	

Table 1. RCTs and observational studies (OR: odds ratio, RR: risk ratio, HR; hazard ratio, CI: confidence interval; LTRA: leukotriene receptor antagonist, ICS: inhaled corticosteroids, FP. Fluticasone propionate, FIS: Fluticasone inhaled suspension; BIS: Budesonide inhalation suspension, LABA: long acting beta2 agonists, SABA: short acting beta2 agonists, MPR: medication possession ratio, ACT: Asthma Control Test; PEF: peak expiratory flow, FEV1: forced expiratory flow in 1 second, FVC: Forced vital capacity (FVC), AEX: asthma exacerbations requiring systemic corticosteroids, MGD: mean group difference)

Title of the study, first author, year [ref]	Type of article	Study design	Population (age)	Mechanisms assessed	Outcome/recommendations	Effect [Relative risk (RR) or odds ratio

						(OR) + confidence interval (CI 95%)]
Definition, assessment and treatment of wheezing disorders in preschool children: an evidence-based approach.<br>**Brand, 2008 [13]**	Systematic review	Literature searches were performed in order to identify material relating to preschool wheeze. 11 relevant study areas were identified.	Preschool children with wheezing<br>(0-5 years)	Each subgroup, (at least 3 people), reviewed the retrieved references for relevant papers, adding additional papers from personal files if required. The evidence was graded as high-, moderate-, low- or very low-grade evidence based on the following criteria: study design and quality (systematic reviews and randomized controlled trials: high quality; observational studies: low quality; any other type of article: very low quality), consistency of the data and relevance. A different, and more readily interpretable, system of categorizing recommendations in four groups was followed: should (or should not) be done, or possibly should (or should not) be done.	Based on the limited evidence available, inhaled short-acting β2-agonists by metered-dose inhaler/spacer combination are recommended for symptomatic relief.<br>Educating parents regarding causative factors and treatment is useful.<br>Exposure to tobacco smoke should be avoided.<br>Allergen avoidance may be considered when sensitization has been established.<br>Maintenance treatment with inhaled corticosteroids is recommended for multiple-trigger wheeze; benefits are often small.<br>Montelukast is recommended for the treatment of episodic (viral) wheeze and can be started when symptoms of a viral cold develop**.**<br>Given the large overlap in phenotypes, and the fact that patients can move from one phenotype to another, inhaled corticosteroids and montelukast may be considered on a trial basis in almost any preschool child with recurrent wheeze but should be discontinued if there is no clear clinical benefit.	-weak evidence<br><br>- weak evidence
Inhaled corticosteroids or montelukast as the preferred primary long-term treatment for pediatric asthma?<br>**Jartti, 2008 [14]**	Systematic review	All randomized double-blind efficacy studies comparing the long-term (>4-week) treatment using a leukotriene receptor antagonist with an inhaled corticosteroid in asthmatic children were critically reviewed.	Children with mild to moderate persistent asthma<br>(0-18 years)	Two main outcome measures were analyzed, forced expiratory volume in 1 s and asthma control days.	In school-aged children, 5 reports with an adequate study design were available. All of these studies compared LTRA with FP. The meta-analysis of the two main outcome measures, forced expiratory volume in 1 s and asthma control days demonstrated the superiority of FP over LTRA. Many other clinical and pulmonary outcomes also consistently showed that low dose inhaled FP was more effective than LTRA in the long-term management of mild to moderate persistent asthma. A more favorable response to FP over LTRA was associated with more severe disease or markers of	-weighted mean difference, 4.6% predicted (95% CI: 3.5–5.5)<br><br>-weighted mean difference 5.6% (95%CI:4.3–6.9)

					allergic inflammation. About a quarter of patients benefited more from LTRA than FP. In children under school age, no comparative studies were available. However, long-term LTRA treatment was found to be effective in placebo-controlled studies in asthmatic children aged >2 years.	
The role of inhaled corticosteroids and montelukast in children with mild-moderate asthma: results of a systematic review with meta-analysis. **Castro-Rodriguez, 2010 [15]**	Meta-analysis	To compare the efficacy of ICS vs LTRA in schoolchildren and adolescents with mild–moderate persistent asthma: Randomized, prospective, controlled trials published January 1996 to November 2009 with a minimum of 4 weeks of ICS versus LTRA and of ICS versus LTRA+ICS were retrieved through Medline, Embase and Central databases. Of 124 studies identified, 18 studies (n=3757 patients) met criteria for inclusion (13 compared ICS vs LTRA, 3 ICS vs LTRA+ICS and 2 ICS vs LTRA vs ICS+LTRA).	patients with asthma (children and adults)	The primary outcome was asthma exacerbations requiring systemic corticosteroids; secondary outcomes were pulmonary function, withdrawal/hospitalization due to AEX, change in symptoms score, rescue medication-free days, albuterol use, adverse effects and adherence.	Patients receiving ICS showed a significantly lower risk for asthma exacerbations requiring systemic corticosteroids (AEX) than those with LTRA; post-hoc analysis suggests this effect was independent of quality, sponsorship and study duration. Children treated with ICS had a significant higher pulmonary function (final FEV1 % predicted, change from baseline FEV1 %, final morning peak expiratory flow (PEF)) and better clinical parameters (albuterol use, symptom score, rescue medication-free days, withdrawals due to AEX) versus LTRA. No significant difference in primary or secondary outcomes was found when LTRA was added on to ICS versus ICS alone; however, these analyses were based on only two studies. School children and adolescents with mild-moderate persistent asthma treated with ICS had less AEX and better lung function and asthma control than with LTRA. There are insufficient data to determine whether the addition of LTRA to ICS improves outcome.	-RR=0.83 (95% CI 0.72-0.96) p=0.01
Anti-leukotriene agents compared to inhaled corticosteroids in the management of recurrent and/or chronic asthma in	Meta-analysis	Randomized trials that compared LTRA with ICS as monotherapy for a minimum period of 4 weeks in patients with asthma aged 2 years and older. 65 trials met the inclusion criteria for this review. 56 trials (19 pediatric	Patients in maintenance treatments for asthma for ≥4 weeks (children aged>2 years and adults)	The primary outcome was the number of patients with at least one exacerbation requiring systemic corticosteroids. Secondary outcomes included patients with at least one exacerbation requiring hospital admission, lung function tests, indices of chronic asthma control, adverse	Patients treated with LTRA were more likely to suffer an exacerbation requiring systemic corticosteroids (N = 6077 participants). For every 28 (95% CI 15 to 82) patients treated with LTRA instead of ICS, there was one additional patient with an exacerbation requiring rescue systemic corticosteroids. The magnitude of effect	-RR: 1.51 (95% CI 1.17-1.96)

adults and children.<br>**Chauhan, 2012 [16]**		trials) contributed data (representing total of 10,005 adults and 3,333 children).<br>21 trials were of high methodological quality; 44 were published in full-text.<br>All trials pertained to patients with mild or moderate persistent asthma.<br>Trial durations varied from four to 52 weeks.		effects, withdrawal rates and biological inflammatory markers.	was significantly greater in patients with moderate compared with those with mild airway obstruction but not influenced by age group (children representing 23% of the weight versus adults). Significant group differences favoring ICS were noted in most secondary outcomes including patients with at least one exacerbation requiring hospital admission (N = 2715 participants), change from baseline FEV1 (N = 7128 participants; mean group difference (MD) 110 mL, 95% CI 140 to 80) as well as other lung function parameters, asthma symptoms, nocturnal awakenings, rescue medication use, symptom free days, the quality of life, parents' and physicians' satisfaction.<br>LTRA therapy was associated with increased risk of withdrawals due to poor asthma control (N = 7669 participants).<br>For every 31 (95% CI 22 to 47) patients treated with LTRA instead of ICS, there was one additional withdrawal due to poor control.<br>Risk of side effects was not significantly different between both groups.	-RR 2.03 (95% CI 1.41-2.91) vs<br>RR 1.25 (95% CI 0.97-1.61)<br><br>- RR 3.33 (95% CI 1.02-10.94)<br><br>- RR 2.56 (95% CI 2.01- 3.27)
Assessment of controversial pediatric asthma management options using GRADE**.**<br>**Boluyt, 2012 [17]**	Systematic review	Health care questions were formulated for 3 controversies in clinical practice:<br>- what is the most effective treatment in asthma not under control with standard-dose ICS (step 3)<br>-the use of LTRA for viral wheeze<br>-the role of extra fine particle aerosols.<br>GRADE was used to rate the quality of evidence and strength of recommendations after performing systematic literature searches.	Children with asthma or viral wheeze<br>(2-18 years)	The aims were to address controversial asthma management issues in children and to illustrate the usefulness of the Grading of Recommendations Assessment,<br>Development and Evaluation (GRADE) approach in rating the quality of evidence and strength of recommendations.	Recommendations:<br>-double the dose of ICS instead of adding a long-acting b-agonist in step 3<br>-ICS instead of LTRA are the first choice in preschool wheeze<br>-extra fine particle ICS formulations are not first-line treatment in children with asthma.	-weak evidence based on low-quality evidence for critical outcomes for all recommendations

Effectiveness of drug treatment strategies to prevent asthma exacerbations and increase symptom-free days in asthmatic children: a network meta-analysis. **Zhao, 2015 [18]**	Meta-analysis	A systematic review with network meta-analysis was conducted after a comprehensive search for relevant studies in the PubMed, Cochrane Library, Embase and Clinical Trials databases, up to July 2014. Randomized clinical trials were selected comparing treatment strategies of the Global Initiative for Asthma guidelines.	Children in maintenance treatments for asthma of ≥4 weeks duration (0-18 years)	The randomized clinical trials compared maintenance treatments for asthma in children (≤18 years) of ≥4 weeks duration, reporting exacerbations or symptom-free days. Included 35 trials with 12 010 patients. The primary and secondary effectiveness outcomes were the rates of moderate/severe exacerbations and symptom-free days from baseline, respectively. Withdrawal rates were taken as the safety outcome	For both primary and secondary outcomes, combined ICS and LABA was ranked first in effectiveness, respectively, compared with low-dose ICS, but the result of secondary outcomes was statistically insignificant.<br>Low-dose ICS, medium- or high-dose ICS and combined ICS and LTRA strategies were comparable in effectiveness. ICS monotherapies, and ICS + LABA and ICS + LTRA strategies were similarly safe. All strategies except LTRA monotherapies were significantly better than placebo. High-dose ICS had the highest rate of total withdrawals, but the difference was not significant. Combined ICS and LABA treatments were most effective in preventing exacerbations among pediatric asthma patients.	-OR 0.70 (95% CI: 0.52–0.97)<br>-OR 1.23 (95% CI: 0.94–1.61)
Principal findings of systematic reviews for chronic treatment in childhood asthma. **Castro-Rodriguez, 2015 [19]**	Systematic review	Systematic reviews of randomized clinical trials (SRCTs) on pharmacologic chronic treatment in children (1–18 years) with persistent asthma. 39 SRCTs were included: two were related to step 1, 24 to step 2, nine to steps 3 and 4, and four to step 5 (according with NAEPP and GINA guidelines). The methodological quality of these SRCTs was determined by using the AMSTAR tool.	Children with asthma (1-18 years)	To summarize the principal findings pertaining to most effective long-term pharmacologic treatment of childhood asthma.<br>The methodological qualification, using the **AMSTAR tool** (total score 11 points), showed that **30 SRCTs had ≥10 points,** 5 had 9 points, and 3 had ≤ 6 points.	For step 1: addition of ipratropium bromide to short-acting beta2-agonists does not show any benefit.<br>For step 2: in preschoolers, ICS reduce severe exacerbations and improve other clinical and lung function parameters. In children, ICSs are superior to LTRA, chromones, or xantines in reducing severe exacerbations, improving lung function and other clinical outcomes. Fluticasone propionate (FP) is better than beclomethasone dipropionate (BDP) or budesonide only for lung function; but similar to hydrofluoroalkane-BDP or to ciclosenide. Compared to low ICS doses, moderate doses result in only better lung function, but this is not true for FP. For steps 3 and 4: adding LTRA to ICS confers a small benefit; adding LABA improves lung function but does not reduce exacerbations more than double or higher ICS doses. For step 5: adding omalizumab decreases exacerbations.	-weak evidence

Preventing Exacerbations in Preschoolers With Recurrent Wheeze: A Meta-analysis. **Kaiser, 2016 [20]**	Meta-analysis	In consultation with a medical librarian, 3 search strategies for 3 databases were created Studies were considered eligible for inclusion if they met criteria regarding population, intervention and comparator, outcomes, and study design. Studies that included only children <2 years were excluded because of the potential overlap with bronchiolitis in this age group.	Children with asthma or recurrent wheezing (≥2 episodes/year) (0-6 years)	Studies comparing the following interventions were included: daily ICS versus placebo, intermittent ICS versus placebo, daily ICS versus intermittent ICS, or any regimen of ICS versus any regimen of montelukast. Any studies that reported on outcome of severe wheezing exacerbations necessitating systemic (oral or intravenous) corticosteroid were included. Severe exacerbations were chosen as our primary outcome because they are a patient-important outcome that have significant consequences for children, caregivers, and the health care system. Only randomized controlled trials were included. Guidelines, reviews, commentaries, abstracts, and letters to editors were reviewed to identify any primary data; however, these publication types were not included because of lack of peer review and inability to judge bias.	22 studies (N = 4550) were included. 15 studies (N = 3278) compared <u>daily ICS with placebo and showed reduced exacerbations with daily medium-dose ICS</u>. Subgroup analysis of children with persistent asthma showed <u>reduced exacerbations with daily ICS compared with placebo</u> (8 studies, N = 2505) and <u>daily ICS compared with montelukast</u> (1 study, N = 202). Subgroup analysis of children with intermittent asthma or viral-triggered wheezing showed <u>reduced exacerbations with preemptive high-dose intermittent ICS compared with placebo</u> (5 studies, N = 422). LIMITATIONS: More studies are needed that directly compare these strategies. There is strong evidence to support daily ICS for preventing exacerbations in preschool children with recurrent wheeze, specifically in children with persistent asthma.	- RR 0.70 (95% CI 0.61–0.79)<br><br>- RR 0.56 (95% CI 0.46–0.70)<br>- RR 0.59 (95% CI, 0.38–0.92)<br><br>- RR 0.65 (95% CI 0.51–0.81)
Daily inhaled corticosteroids or montelukast for preschoolers with asthma or recurrent wheezing: A systematic review. **Castro-Rodriguez, 2018 [21]**	Systematic review	To compare the efficacy of daily ICS versus LTRA in preschoolers with asthma or recurrent wheezing. Randomized, prospective, controlled trials published by December2017, with a minimum of 3-month therapy with daily ICS versus LTRA were identified. Of 29 trials identified, six studies (n= 3204 patients, 62% males, age range: 6-54 months) met the inclusion criteria; two were at low risk of bias. Five pertained to children with asthma; one to those with recurrent wheezing. No outcomes were similarly	Preschool children with wheezing (6-54 months)	The co-primary outcomes were the number of wheezing episodes and daily symptom score. Secondary outcomes included unscheduled emergency visits, need of rescue systemic corticosteroids (SC), hospitalization for exacerbations, lung function, and adverse effects.	Due to the inability to conduct a meta-analysis for any primary or secondary outcomes, this review reported a narrative synthesis of the outcomes. Based on trials at lowest risk of bias and the largest open-labelled studies, <u>ICS was associated with better control of symptoms and less exacerbations than LTRA</u>. And also less need for rescue systemic corticosteroids. Insufficient data of high quality prevented firm conclusions on other secondary outcomes.	-weak evidence

		reported in the six studies, preventing meta-analysis.				

Table 2. Meta-analysis and systematic reviews (OR: odds ratio, RR: risk ratio, HR; hazard ratio, CI: confidence interval; LTRA: leukotriene receptor antagonist, ICS: inhaled corticosteroids, FP. Fluticasone propionate, FIS: Fluticasone inhaled suspension; BIS: Budesonide inhalation suspension, LABA: long acting beta2 agonists, SABA: short acting beta2 agonists, MPR: medication possession ratio, ACT: Asthma Control Test; PEF: peak expiratory flow, FEV1: forced expiratory flow in 1 second, FVC: Forced vital capacity (FVC), AEX: asthma exacerbations requiring systemic corticosteroids, MGD: mean group difference)

References

1. Kooi EM, Schokker S, Marike Boezen H, de Vries TW, Vaessen-Verberne AA, van der Molen T, Duiverman EJ. Fluticasone or montelukast for preschool children with asthma-like symptoms: Randomized controlled trial. Pulm Pharmacol Ther. 2008 Oct;21(5):798-804. doi: 10.1016/j.pupt.2008.06.004. Epub 2008 Jul 2. PMID: 18647656.
2. Elkout H, Helms PJ, Simpson CR, McLay JS. Adequate levels of adherence with controller medication is associated with increased use of rescue medication in asthmatic children. PLoS One. 2012;7(6):e39130. doi: 10.1371/journal.pone.0039130. Epub 2012 Jun 27. PMID: 22761728; PMCID: PMC3384638.
3. Ducharme FM, Noya FJ, Allen-Ramey FC, Maiese EM, Gingras J, Blais L. Clinical effectiveness of inhaled corticosteroids versus montelukast in children with asthma: prescription patterns and patient adherence as key factors. Curr Med Res Opin. 2012 Jan;28(1):111-9. doi: 10.1185/03007995.2011.640668. Epub 2011 Dec 16. PMID: 22077107.
4. Szefler SJ, Carlsson LG, Uryniak T, Baker JW. Budesonide inhalation suspension versus montelukast in children aged 2 to 4 years with mild persistent asthma. J Allergy Clin Immunol Pract. 2013 Jan;1(1):58-64. doi: 10.1016/j.jaip.2012.08.005. Epub 2012 Nov 8. PMID: 24229823.
5. Wu CL, Andrews AL, Teufel RJ 2nd, Basco WT Jr. Demographic predictors of leukotriene antagonist monotherapy among children with persistent asthma. J Pediatr. 2014 Apr;164(4):827-831.e1. doi: 10.1016/j.jpeds.2013.11.029. Epub 2013 Dec 25. PMID: 24370344.
6. Wu AC, Li L, Fung V, Kharbanda EO, Larkin EK, Vollmer WM, Butler MG, Miroshnik I, Rusinak D, Davis RL, Hartert T, Weiss ST, Lieu TA. Use of leukotriene receptor antagonists are associated with a similar risk of asthma exacerbations as inhaled corticosteroids. J Allergy Clin Immunol Pract. 2014 Sep-Oct;2(5):607-13. doi: 10.1016/j.jaip.2014.05.009. Epub 2014 Jul 25. PMID: 25213056; PMCID: PMC6557573.
7. Krawiec M, Strzelak A, Krenke K, Modelska-Wozniak I, Jaworska J, Kulus M. Fluticasone or montelukast in preschool wheeze: a randomized controlled trial. Clin Pediatr (Phila). 2015 Mar;54(3):273-81. doi: 10.1177/0009922814550158. Epub 2014 Sep 22. PMID: 25246602.
8. Fitzpatrick AM, Jackson DJ, Mauger DT, Boehmer SJ, Phipatanakul W, Sheehan WJ, Moy JN, Paul IM, Bacharier LB, Cabana MD, Covar R, Holguin F, Lemanske RF Jr, Martinez FD, Pongracic JA, Beigelman A, Baxi SN, Benson M, Blake K, Chmiel JF, Daines CL, Daines MO, Gaffin JM, Gentile DA, Gower WA, Israel E, Kumar HV, Lang JE, Lazarus SC, Lima JJ, Ly N, Marbin J, Morgan W, Myers RE, Olin JT, Peters SP, Raissy HH, Robison RG, Ross K, Sorkness CA, Thyne SM, Szefler SJ; NIH/NHLBI AsthmaNet. Individualized therapy for

persistent asthma in young children. J Allergy Clin Immunol. 2016 Dec;138(6):1608-1618.e12. doi: 10.1016/j.jaci.2016.09.028. Epub 2016 Oct 21. PMID: 27777180; PMCID: PMC5148729.

9. Zhang Y, Wang H. Efficacy of montelukast sodium chewable tablets combined with inhaled budesonide in treating pediatric asthma and its effect on inflammatory factors. Pharmazie. 2019 Nov 1;74(11):694-697. doi: 10.1691/ph.2019.9582. PMID: 31739840.
10. Levine H, Leventer-Roberts M, Hoshen M, Mei-Zahav M, Balicer R, Blau H. Healthcare utilization in infants and toddlers with asthma-like symptoms. Pediatr Pulmonol. 2019 Oct;54(10):1567-1577. doi: 10.1002/ppul.24429. Epub 2019 Jul 12. PMID: 31298808.
11. Shin J, Oh SJ, Petigara T, Tunceli K, Urdaneta E, Navaratnam P, Friedman HS, Park SW, Hong SH. Comparative effectiveness of budesonide inhalation suspension and montelukast in children with mild asthma in Korea. J Asthma. 2020 Dec;57(12):1354-1364. doi: 10.1080/02770903.2019.1648504. Epub 2019 Aug 6. PMID: 31386600.
12. Chen ZM, Zhao DY, Xiang L, Hong JG. Treatment of pediatric mild persistent asthma with low-dose budesonide inhalation suspension vs. montelukast in China. World J Pediatr. 2021 Dec;17(6):619-625. doi: 10.1007/s12519-021-00464-7. Epub 2021 Oct 6. PMID: 34613593.
13. Brand PL, Baraldi E, Bisgaard H, Boner AL, Castro-Rodriguez JA, Custovic A, de Blic J, de Jongste JC, Eber E, Everard ML, Frey U, Gappa M, Garcia-Marcos L, Grigg J, Lenney W, Le Souëf P, McKenzie S, Merkus PJ, Midulla F, Paton JY, Piacentini G, Pohunek P, Rossi GA, Seddon P, Silverman M, Sly PD, Stick S, Valiulis A, van Aalderen WM, Wildhaber JH, Wennergren G, Wilson N, Zivkovic Z, Bush A. Definition, assessment and treatment of wheezing disorders in preschool children: an evidence-based approach. Eur Respir J. 2008 Oct;32(4):1096-110. doi: 10.1183/09031936.00002108. PMID: 18827155.
14. Jartti T. Inhaled corticosteroids or montelukast as the preferred primary long-term treatment for pediatric asthma? Eur J Pediatr. 2008 Jul;167(7):731-6. doi: 10.1007/s00431-007-0644-3. Epub 2008 Jan 24. Erratum in: Eur J Pediatr. 2008 Jul;167(7):737-8. PMID: 18214538.
15. Castro-Rodriguez JA, Rodrigo GJ. The role of inhaled corticosteroids and montelukast in children with mild-moderate asthma: results of a systematic review with meta-analysis. Arch Dis Child. 2010 May;95(5):365-70. doi: 10.1136/adc.2009.169177. Epub 2009 Nov 27. PMID: 19946008.
16. Chauhan BF, Ducharme FM. Anti-leukotriene agents compared to inhaled corticosteroids in the management of recurrent and/or chronic asthma in adults and children. Cochrane Database Syst Rev. 2012 May 16;2012(5):CD002314. doi: 10.1002/14651858.CD002314.pub3. PMID: 22592685; PMCID: PMC4164381.
17. Boluyt N, Rottier BL, de Jongste JC, Riemsma R, Vrijlandt EJ, Brand PL. Assessment of controversial pediatric asthma management options using GRADE. Pediatrics. 2012 Sep;130(3):e658-68. doi: 10.1542/peds.2011-3559. Epub 2012 Aug 27. PMID: 22926178.

18. Zhao Y, Han S, Shang J, Zhao X, Pu R, Shi L. Effectiveness of drug treatment strategies to prevent asthma exacerbations and increase symptom-free days in asthmatic children: a network meta-analysis. J Asthma. 2015 Oct;52(8):846-57. doi: 10.3109/02770903.2015.1014101. Epub 2015 Jun 10. PMID: 26061910.
19. Castro-Rodriguez JA, Rodrigo GJ, Rodriguez-Martinez CE. Principal findings of systematic reviews for chronic treatment in childhood asthma. J Asthma. 2015 May;52(4):407-16. doi: 10.3109/02770903.2014.971968. Epub 2014 Oct 29. PMID: 25275887.
20. Kaiser SV, Huynh T, Bacharier LB, Rosenthal JL, Bakel LA, Parkin PC, Cabana MD. Preventing Exacerbations in Preschoolers With Recurrent Wheeze: A Meta-analysis. Pediatrics. 2016 Jun;137(6):e20154496. doi: 10.1542/peds.2015-4496. PMID: 27230765.
21. Castro-Rodriguez JA, Rodriguez-Martinez CE, Ducharme FM. Daily inhaled corticosteroids or montelukast for preschoolers with asthma or recurrent wheezing: A systematic review. Pediatr Pulmonol. 2018 Dec;53(12):1670-1677. doi: 10.1002/ppul.24176. Epub 2018 Nov 5. PMID: 30394700.

PICO question 10. How long should controller therapy with ICS be continued?

Patient or population: children with preschool wheezing

Setting: from primary to tertiary care

Intervention: daily ICS

Comparison: discontinuation of ICS

Search strategy:

(((child, preschool[MeSH Terms]) OR (toddler*)) AND ((Respiratory Sounds[Mesh]) OR (wheez*) OR ("Asthma"[Mesh:NoExp])) AND ((("Administration, Inhalation"[Mesh]) OR (Inhaled)) AND ((ICS) OR (Adrenal Cortex Hormones[Mesh]) OR (*steroid*)))) AND ((maintenance therapy) OR (chronic therapy) OR(controller Therapy))

PICO n° 10 Workflow of study selection process

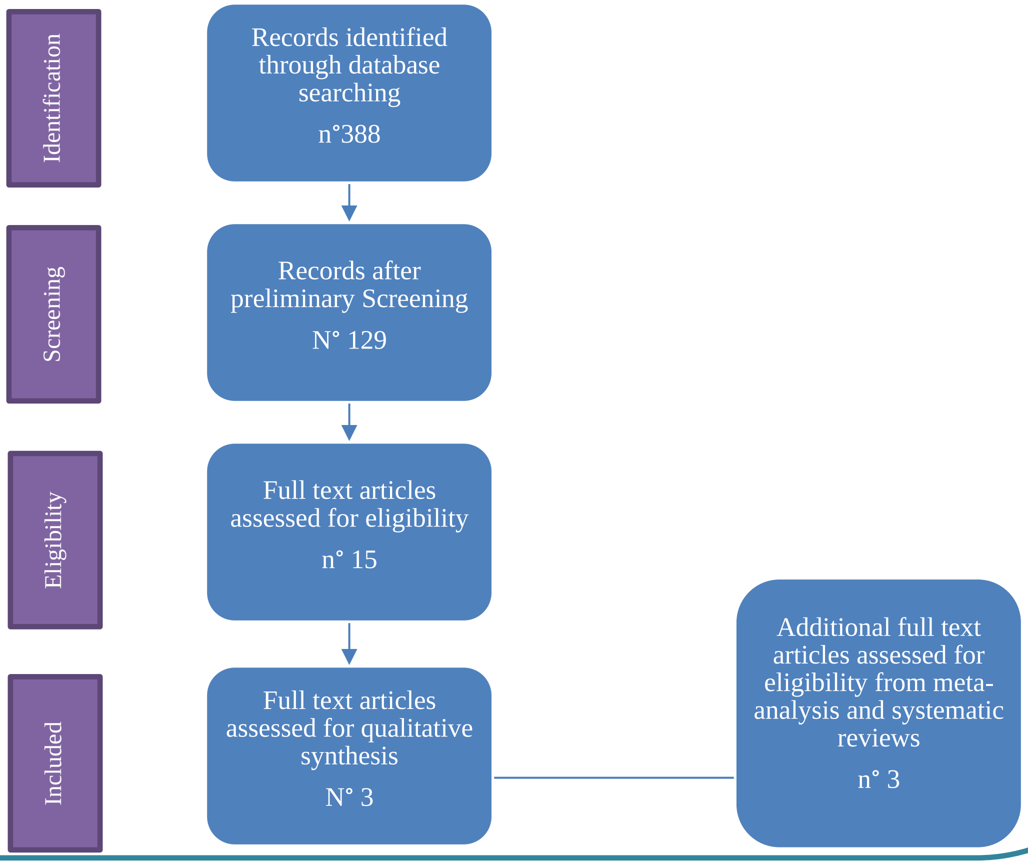

Modified from: Moher D, Liberati A, Tetzlaff J, Altman DG, The PRISMA Group (2009). Preferred Reporting Items for Systematic Reviews and Meta-Analyses: The PRISMA Statement. PLoS Med 6(7): e1000097

Title of the study, first author, year [ref]	Type of study	Study design	Population	N of patients (age)	Experiments/mechanisms assessed	Outcome (primary and secondary)
Fluticasone or montelukast for preschool children with asthma-like symptoms: randomized controlled trial.<br>**Kooi, 2008 [1]**	Multi-center, double-blind, double-dummy, randomized placebo-controlled trial	Daily montelukast 4 mg (Mk) (n 18) vs daily fluticasone propionate 100 mcg (FP) twice daily (n 25) vs daily placebo (n 20) for 3 months	Children with asthma-like symptoms (wheeze, cough and shortness of breath)	63 (2-5 years)	Assessment at baseline and after 3 months of:<br>Symptoms scores<br><br>Rescue medication free days<br><br>Blood eosinophils<br><br>Lung function<br><br>Adverse events	A statistically significant difference in change in daily symptom score was found between FP and placebo in favor of the FP group (p=0.021). Symptoms scores decreased after 3 months in all groups, but it was higher in the fluticasone group (p=0.001).<br><br>No difference between groups.<br>A significant reduction in rescue medication free days at baseline and 3 months was found within the ICS group (p=0.015), as well as in the other 2 groups.<br><br>Decrease in montelukast group after 3 months (p=0.008)<br><br>Except for a significant lower frequency dependence of resistance in the FP group as compared to the Mk group after 3 months of treatment (p=0.48), no significant changes in lung function were found.<br><br>In the Mk and FP group, more children experienced upper respiratory tract infections as compared to the placebo group (p=0.011)
Regular vs prn nebulized treatment in wheeze preschool children.<br>**Papi, 2009 [2]**	Multicenter, randomized, parallel-group, three-group, doubleblind, double-dummy, placebo-controlled study	Daily beclomethasone (400 mcg x 2 times/day) + as needed SABA (n 110) vs daily placebo + as needed ICS/SABA (800 mcg beclomethasone + 1600 mcg salbutamol) (n 110) vs placebo + as needed SABA (n 56)<br>for 12 weeks	Children with at least three episodes of wheezing requiring medical attention in the previous 6 months	276 (1-4 years)	Symptom-free days<br><br>Daytime symptom score<br><br>Night-time symptom score<br><br>No. of nocturnal awakenings	More in regular beclomethasone vs as needed salbutamol (69.6% ± 20.89 vs 61.0 ± 24.83), p = 0.034<br><br>Higher in the as needed salbutamol group: 0.55 vs 0.32 in the beclomethasone (p = 0.045) and vs 0.32 in the as needed combination (p = 0.037).<br><br>Higher in the as needed salbutamol group: 0.43 vs 0.23 in the beclomethasone group (p=0.039) and vs 0.19 in the as needed combination (p=0.014)<br><br>Higher in the as needed salbutamol group: 0.17 vs 0.05 in the beclomethasone group (p <0.001) and vs 0.05 in the as needed combination (p <0.001)

					No. of nocturnal awakenings of the parent<br><br>Use of rescue medications<br><br>Adverse events	Higher in the as needed salbutamol group: 0.22 vs 0.11 in the beclomethasone group (p=0.020) and vs 0.11 in the as needed combination (p=0.018)<br><br>No difference<br><br>No difference
Patient Characteristics Associated with Improved Outcomes with Use of an Inhaled Corticosteroid in Preschool Children at Risk for Asthma.<br>**Bacharier, 2009 [3]**	Multi-center, double-blind, randomized, placebo-controlled, parallel-group comparison trial (post-hoc analysis)	Administration of daily fluticasone propionate, 44 µg/puff, 2 puffs twice daily (n 143) vs placebo (n 142) for 2 years, followed by 1 year of observation	Children with a positive, mAPI[5] consisting of frequent wheezing and either one major risk factor or two of three minor risk factors	285 (2-3 years)	Episode-free days (EFD)<br><br>Number of systemic corticosteroid courses<br><br>Urgent care visits<br><br>Use of any supplementary controller medication<br><br>Symptoms control after 2 years of treatment	During the two-year treatment period, the proportion of episode-free days was significantly greater in the fluticasone group than in the placebo group (93.2% [95% CI 91.1 to 94.9 %] vs. 88.4% [95 %CI 84.9 to 91.2 %], P=0.006<br><br>ICS group had a lower rate of exacerbations necessitating a course of systemic corticosteroids than placebo group (57.4 per 100 child-years [95 % CI, 49.0 to 67.3] vs. 89.4 per 100 child-years [95 % CI, 78.3 to 102.2], P<0.001)<br><br>No difference<br><br>ICS group had a lower rate of montelukast use (a mean of 11.4 days per year [95 % CI, 9.3 to 13.7] vs. a mean of 24.2 days per year [95 % CI, 20.5 to 27.9], P <0.001), and a lower supplementary use of fluticasone (a mean of 8.3 days per year [95 % CI, 6.8 to 10.4] vs. a mean of 17.6 days per year [95 % CI, 14.9 to 20.9], P<0.001)<br><br>Once ICS therapy was discontinued, participants who received ICS therapy for 2 years did not have an advantage in terms of asthma symptoms during the third year of the study
Ciclesonide in wheezy preschool children with a positive asthma predictive index or atopy.<br>**Brand, 2011 [4]**	Multicentre, randomised, double-blind, placebo-controlled, parallel-group study	Daily ICS (ciclesonide 40/80/160 mcg) vs placebo (24 weeks)	MTWheezers with positive API[2] or screening for atopy	874 (224 ICS 40 mcg, 215 ICS 80 mcg, 229 ICS 160 mcg, 206 placebo)	Risk of acute exacerbation[3]	Placebo 10.2% vs ciclesonide 6.2%<br>RR 1.65, CI 95% 1.04-2.63

[1]Results are shown for regular beclomethasone, as-needed combination and as needed salbutamol, respectively
[2]Parental history of asthma, personal history of atopic dermatitis, or aeroallergen sensitization
[3]Placebo group vs pooled ICS group

[4]All results reported refer to the ICS group at baseline, 2, 4, 8, 12 weeks of treatment. No p-value reported in the study.
[5]Peripheral blood eosinophilia ≥4%, wheezing without colds, or allergic sensitization to food

References

1. Kooi EM, Schokker S, Marike Boezen H, de Vries TW, Vaessen-Verberne AA, van der Molen T, Duiverman EJ. Fluticasone or montelukast for preschool children with asthma-like symptoms: Randomized controlled trial. Pulm Pharmacol Ther. 2008 Oct;21(5):798-804. doi: 10.1016/j.pupt.2008.06.004. Epub 2008 Jul 2. PMID: 18647656.
2. Papi A, Nicolini G, Baraldi E, Boner AL, Cutrera R, Rossi GA, Fabbri LM; BEclomethasone and Salbutamol Treatment (BEST) for Children Study Group. Regular vs prn nebulized treatment in wheeze preschool children. Allergy. 2009 Oct;64(10):1463-1471. doi: 10.1111/j.1398-9995.2009.02134.x. PMID: 19772514.
3. Bacharier LB, Guilbert TW, Zeiger RS, Strunk RC, Morgan WJ, Lemanske RF Jr, Moss M, Szefler SJ, Krawiec M, Boehmer S, Mauger D, Taussig LM, Martinez FD; Childhood Asthma Research and Education Network of the National Heart, Lung, and Blood Institute. Patient characteristics associated with improved outcomes with use of an inhaled corticosteroid in preschool children at risk for asthma. J Allergy Clin Immunol. 2009 May;123(5):1077-82, 1082.e1-5. doi: 10.1016/j.jaci.2008.12.1120. Epub 2009 Feb 23. PMID: 19230959; PMCID: PMC2909590.
4. Brand PL, Luz García-García M, Morison A, Vermeulen JH, Weber HC. Ciclesonide in wheezy preschool children with a positive asthma predictive index or atopy. Respir Med. 2011 Nov;105(11):1588-95. doi: 10.1016/j.rmed.2011.07.017. Epub 2011 Aug 11. PMID: 21839625.

PICO question 11: Is intermittent therapy starting after symptoms onset with ICS preferred to daily therapy?

Patient or population: children with preschool wheezing

Setting: primary care

Intervention: intermittent high dose ICS

Comparison: daily ICS

Search strategy:

("child, preschool"[MeSH Terms] OR "toddler"[All Fields]) AND ("respiratory sounds"[MeSH Terms] OR "wheez*"[All Fields]) AND ("intermittent steroid*" OR "daily steroid*" OR "maintainance therapy" OR "ICS" OR "inhaled corticosteroid"[All Fields] OR "beclomethasone"[MeSH Terms] OR "fluticasone"[MeSH Terms] OR "Adrenal Cortex Hormones/therapeutic use"[MAJR]) AND ((2008:2021[pdat]) AND (english[Filter]))

PICO n° 11. Workflow of study selection process

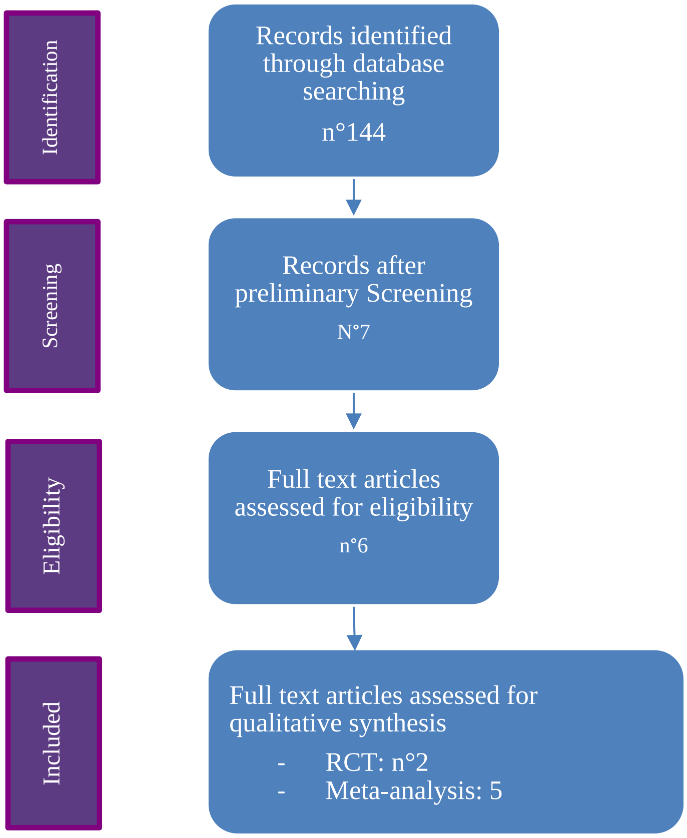

Modified from: Moher D, Liberati A, Tetzlaff J, Altman DG, The PRISMA Group (2009). Preferred Reporting Items for Systematic Reviews and Meta-Analyses: The PRISMA Statement. PLoS Med 6(7): e1000097

Title of the study, first author, year [ref]	Type of study	Study design	N of patients (age)	Experiments/mechanisms assessed	Outcomes (primary and secondary)	Results	Effect [Relative risk (RR) or odds ratio (OR) + confidence interval (CI 95%)]
Daily or Intermittent Budesonide in Preschool Children with Recurrent Wheezing. **Zeiger, 2011 [1]**	Randomized, double-blind, parallel-group trial	Children were randomly assigned to receive either an intermittent high-dose regimen of budesonide (1 mg twice daily for 7 days, starting early during a predefined respiratory tract illness (n 139) or a daily low-dose regimen of budesonide (0.5 mg nightly) (n 139) and as-needed albuterol	278 (12-53 months)	Daily low dose of budesonide<br>VS<br>Intermittent high-dose budesonide during respiratory tract illness	Frequency of exacerbations (rate per patient-year)	No difference	Daily: 0.97 CI 0.76 to 1.22<br>Intermittent: 0.95 CI, 0.75 to 1.20
					Time to the first exacerbation	No difference	hazard ratio, 0.97; CI, 0.76 to 1.22; P=0.87
					Rate of treatment failure,	No difference	P=0.12
					No. of treatments for respiratory tract illness	No difference	Event rate/Person year:<br>Intermittent: 3.61 CI (3.13 to 4.16)<br>Daily: 3.27 CI (2.82 to 3.79)
					No. of urgent care visits for asthma	No difference	Event rate/Person year:<br>Intermittent: 2.37 (1.89 to 2.97)<br>Daily: 2.40 (1.91 to 3.02)<br>Relative rate: 0.99 (0.72 to 1.35)
					Respiratory tract illnesses in which prednisolone was administered	No difference	Proportion:<br>Intermittent: 0.24 CI (0.19 to 0.29)<br>Daily: 0.26 (0.21 to 0.32)<br>Relative proportion: 0.90 (0.68 to 1.19)
					No. of days absent from work, school, or day care	No difference	Event rate/Person year:<br>Intermittent: 2.72 (2.00 to 3.70)<br>Daily: 3.02 (2.22 to 4.12)<br>Relative rate: 0.90 (0.59 to 1.37)
					Days with albuterol use (%)	No difference	Mean value<br>Intermittent: 6 CI (5 to 7)<br>Daily: 5 CI (4 to 6)<br>Mean difference<br>0.4 (−1.0 to 2.0)
					Episode-free days (%)	No difference	Mean value<br>Intermittent: 78 (75 to 80)<br>Daily : 78 (76 to 81)<br>Mean difference<br>−0.7 (−4.0 to 2.0)
Regular vs prn nebulized treatment in wheeze preschool children.	Double-blind, double-dummy, randomized,	For 12 weeks one group (n 110) received a daily dose of 400 µg of beclomethasone plus 2500 µg of salbutamol during the acute	276 (1-4 years)	Regular beclomethasone;<br>VS<br>Regular placebo + prn combination of high dose	Symptom-free days (mean percentage)	Regular beclomethasone (69.6%), > prn salbutamol (61.0 %)	Regular vs prn salb P = 0.034; [0.58; 13.15]

**Papi, 2009 [2]**	parallel-group trial	respiratory event, another group (n 110) received daily placebo plus a high doses of beclomethasone 800 µg / 1600 µg salbutamol only during an acute respiratory event, and in the last group (n 56) were administrated daily placebo and 2500 µg of salbutamol during the acute respiratory event.		beclomethasone and salbutamol<br>VS<br>Regular placebo +Prn salbutamol		No statistically significant difference between prn salbutamol (61%) and prn combination (64.9%)	Regular vs prn combination: P = 0.293; [–2.38; 7.62]<br>Prn comb Vs prn salb: P = 0.248; [–2.38; 10.71]
					No. of nocturnal awakenings (children)	Regular beclomethasone (0.05) = prn combination (0.05)> prn salbutamol (0.17)	Regular vs prn salb: P < 0.001; [–0.19; –0.05] Regular vs prn combination: P = 0.939; [–0.06; 0.05]<br>Prn comb Vs prn salb P < 0.001; [–0.18; –0.05]
					Daytime rescue medication, no. of uses	Regular beclomethasone (0.09) = prn combination (0.09)> prn salbutamol (0.16)	Regular vs prn salb P = 0.156; [–0.18; 0.03<br>Regular vs prn combination: P = 0.995; [–0.08; 0.09]<br>Prn comb Vs prn salb: P = 0.151; [0.18; 0.03]
					Night-time rescue medication, no. of uses	Regular beclomethasone (0.04) = prn combination (0.04)> prn salbutamol (0.18)	Regular vs prn salb: P = 0.095; [–0.09; 0.01]<br>Regular vs prn combination: P = 0.909; [–0.04; 0.04]<br>Prn comb Vs prn salb: P = 0.077; [–0.09; 0.00]
					Proportion of children with exacerbations		Daily (1.8%), intermittent (5.5%), placebo (9%)
Preventing Exacerbations in Preschoolers With Recurrent Wheeze: A Meta-analysis. **Kaiser, 2016 [3]**	Meta-analysis	Subgroup daily ICS (n 249) Vs intermittent ICS (n249)	498	Daily ICS<br>VS<br>Intermittent ICS	Severe exacerbation	No difference<br>Daily (25.7%)<br>Intermittent 28.1%,	RR 0.91(0.71-1.18)
						No difference in symptom free days	
Daily vs. intermittent inhaled corticosteroids for recurrent wheezing and mild persistent asthma: A systematic review with meta-analysis. **Rodrigo, 2014 [4]**	Meta-analysis			Daily ICS<br>VS<br>Intermittent ICS	Frequency of exacerbation		RR Z 0.96; 95% CI, 0.86, 1.06

Intermittent versus daily inhaled corticosteroids for persistent asthma in children and adults. **Chauhan, 2011 [5]**	Meta-analysis	6 trials:<br>-4 pediatrics (2 preschool childen)<br>-2 adults	1211<br>Preschool child: 498	Daily ICS<br>Vs<br>Intermittent ICS	Exacerbations requiring oral corticosteroids	No difference	RR 1.07; 95% CI 0.87 to 1.32;

References

1. Zeiger RS, Mauger D, Bacharier LB, Guilbert TW, Martinez FD, Lemanske RF Jr, Strunk RC, Covar R, Szefler SJ, Boehmer S, Jackson DJ, Sorkness CA, Gern JE, Kelly HW, Friedman NJ, Mellon MH, Schatz M, Morgan WJ, Chinchilli VM, Raissy HH, Bade E, Malka-Rais J, Beigelman A, Taussig LM; CARE Network of the National Heart, Lung, and Blood Institute. Daily or intermittent budesonide in preschool children with recurrent wheezing. N Engl J Med. 2011 Nov 24;365(21):1990-2001. doi: 10.1056/NEJMoa1104647. PMID: 22111718; PMCID: PMC3247621.
2. Papi A, Nicolini G, Baraldi E, Boner AL, Cutrera R, Rossi GA, Fabbri LM; BEclomethasone and Salbutamol Treatment (BEST) for Children Study Group. Regular vs prn nebulized treatment in wheeze preschool children. Allergy. 2009 Oct;64(10):1463-1471. doi: 10.1111/j.1398-9995.2009.02134.x. PMID: 19772514.
3. Kaiser SV, Huynh T, Bacharier LB, Rosenthal JL, Bakel LA, Parkin PC, Cabana MD. Preventing Exacerbations in Preschoolers With Recurrent Wheeze: A Meta-analysis. Pediatrics. 2016 Jun;137(6):e20154496. doi: 10.1542/peds.2015-4496. PMID: 27230765.
4. Rodrigo GJ, Castro-Rodríguez JA. Daily vs. intermittent inhaled corticosteroids for recurrent wheezing and mild persistent asthma: a systematic review with meta-analysis. Respir Med. 2013 Aug;107(8):1133-40. doi: 10.1016/j.rmed.2013.05.005. Epub 2013 Jun 14. PMID: 23769720.
5. Chauhan BF, Chartrand C, Ducharme FM. Intermittent versus daily inhaled corticosteroids for persistent asthma in children and adults. Cochrane Database Syst Rev. 2012 Dec 12;12:CD009611. doi: 10.1002/14651858.CD009611.pub2. Update in: Cochrane Database Syst Rev. 2013;2:CD009611. PMID: 23235678.

PICO question 12. In case of use of LTRA as controller therapy, how long should be continued?

Patient or population: children with preschool wheezing
Setting: from primary to tertiary care
Intervention: daily LTRA
Comparison: discontinuation of LTRA

Search strategy
(((child, preschool[MeSH Terms]) OR (toddler*)) AND ((Respiratory Sounds[Mesh]) OR (wheez*) OR ("Asthma"[Mesh:NoExp])) AND (("Leukotriene Antagonists"[Mesh]) OR (montelukast)) AND ((maintenance therapy) OR (chronic therapy) OR (Controller therapy))

PICO n° 12 Workflow of study selection process

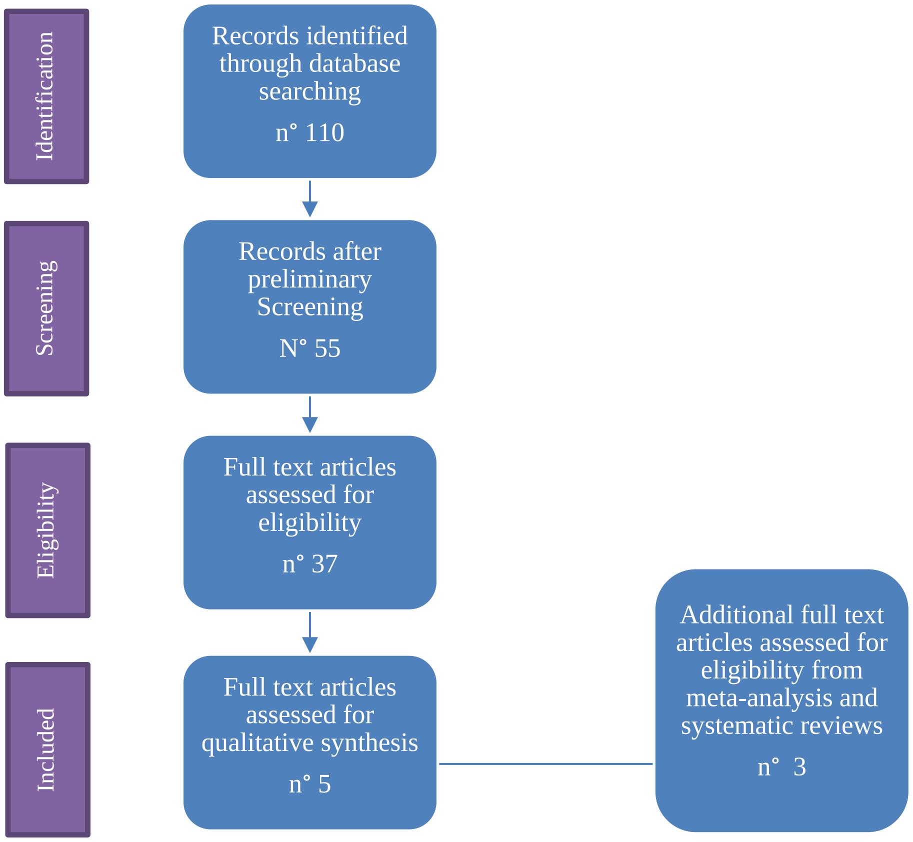

Modified from: Moher D, Liberati A, Tetzlaff J, Altman DG, The PRISMA Group (2009). Preferred Reporting Items for Systematic Reviews and Meta-Analyses: The PRISMA Statement. PLoS Med 6(7): e1000097

Title of the study, first author, year [ref]	Type of study	Study design	Population	N of patients (Age)	Experiments/mechanisms assessed	Outcome (primary and secondary)
Fluticasone or montelukast for preschool children with asthma-like symptoms: randomized controlled trial. **Kooi, 2008 [1]**	Multicenter, double-blind, double-dummy, randomized placebo-controlled trial	Daily montelukast (Mk) 4 mg (n 18) vs daily fluticasone propionate (FP) 100 mcg twice daily (n 25) vs daily placebo (n 20) for 3 months	Children with asthma-like symptoms (wheeze, cough and shortness of breath)	63 (2-5 years)	Symptoms scores<br><br>Rescue medication free days<br><br>Blood eosinophils<br><br>Lung function<br><br>Adverse events	No statistically significant difference between LTRA[a] group and placebo group in symptoms score.<br>A significant reduction between symptoms scores at baseline and after 3 months was found within the LTRA group (p=0.025)<br><br>No statistically significant difference between the 3 groups in terms of rescue medication free days.<br>A significant reduction in rescue medication free days at baseline and after 3 months was found within the LTRA group (p=0.001), as well as in the other 2 groups[1]<br><br>A statistically significant decrease in blood eosinophils after 3 months was found in the Mk group only (p=0.008)<br><br>Except for a significant lower frequency dependence of resistance in the FP group as compared to the Mk group after 3 months of treatment (p=0.048), no significant changes in lung function were found either within or between treatment groups.<br><br>In the Mk and FP group more children experienced upper respiratory tract infections as compared to the placebo group (p=0.011)
Effects of Montelukast on Subjective and Objective Outcome Measures in Preschool Asthmatic Children. **Moeller, 2008 [2]**	Cohort	Daily montelukast (n 12) as first line treatment or as add on therapy administered if FeNO[b] levels were above 9 ppb (for 8 weeks) vs no added therapy (n 13)	Asthmatic children	25 (2.5-5 years)	FeNO levels<br><br>Bronchial reactivity to AMP<br><br>Symptom scores	Decrease in FeNO of 34.8 +/- 39% (p=0.011) in LTRA added group<br><br>Increase in FeNO of 96.8 +/- 126% (p=0.018) in no added therapy group<br><br>The provocative AMP-dose increased significantly in the LTRA group (p=0.015) but not in no added therapy group (p=0.12)<br><br>Symptom scores in LTRA group were reduced after 8 weeks (p=0.034), whereas no change was observed for no added therapy group
Montelukast as Monotherapy in Children with Mild Persistent Asthma. **Wu, 2009[2] [3]**	Observational	Daily LTRA (montelukast 4 mg) for 12 weeks	Asthmatic children	597 (2-5 years subgroup)	Daytime symptoms score<br><br>Nighttime symptoms score<br><br>Mean Score of Investigators' Global Evaluation after Montelukast Treatment<br><br>SABA[c] usage	Daytime symptoms score at baseline, 4, 8, 12 weeks:<br>5.5 ± 3.7, 2.5 ± 2.4, 1.6 ± 2.2, 1.0 ± 1.7 ($p< 0.0083$)[3]<br><br>Nighttime symptoms score at baseline, 4, 8, 12 weeks:<br>2.0 ± 1.1, 1.0 ± 0.9, 0.8 ± 0.9, 0.5 ± 0.7 ($p< 0.0083$)[3]<br><br>Mean Score of Investigators' Global Evaluation After Montelukast Treatment at 4, 8, 12 weeks:<br>1.4 ± 0.9, 1.7 ± 0.9, 1.9 ± 0.9 ($p< 0.0083$)[4]<br><br>SABA usage: average number of puffs at baseline, 4, 8, 12 weeks:<br>0.5 ± 2.1, 0.2 ± 1.5, 0.3 ± 1.9, 0.3 ± 1.6 ($p< 0.0083$)[3]

					Hospitalizations<br><br>ER visits	Hospitalization at baseline, 4, 8, 12 weeks:<br>0.11 ± 0.32, 0.01 ± 0.10, 0.01 ± 0.09, 0.02 ± 0.14 (p< 0.0083)[3]<br><br>ER[d] visits at baseline, 4, 8, 12 weeks:<br>0.08 ± 0.27, 0.02 ± 0.12, 0.01 ± 0.11, 0.01 ± 0.14 (p< 0.0083)[3]
Budesonide Inhalation Suspension Versus Montelukast in Children Aged 2 to 4 Years with Mild Persistent Asthma. **Szefler, 2013 [4]**	Open-label, randomized active-controlled, multicenter study	Daily LTRA (montelukast 4 mg once daily; n 98) vs daily ICS[e] (budesonide 0.5 mg once daily; n 105)	Mild persistent asthma	203 (2-4 years)	Additional asthma medication<br><br>Additional oral steroids<br><br>Physician Global assessment of asthma control	Use of additional asthma medication over 52 weeks of treatment: 61 patients (58.1%) ICS group compared with 64 patients (66.1%) LTRA group (not statistically significant)<br><br>Percentages of patients who required oral steroid therapy at 12, 26, and 52 weeks in ICS vs LTRA group:<br>8.6% versus 18.6% (p=0.044),<br>14.3% versus 27.8% (p=0.029),<br>21.9% versus 37.1% (p=0.022), respectively<br><br>Physician Global Assessment in LTRA group:<br>Asthma symptoms: 42% better after 3 months, 45.5% after 1 year<br>Ability to manage asthma: 43.2% improved after 3 months, 44.4% after 1 year
To determine the efficacy of inhaled corticosteroids compared to montelukast in reducing exacerbation in uncontrolled asthma in children 6 months to 5 years. **Jehan, 2014 [5]**	Randomized parallel group, open-label/ single center trial	Daily LTRA (montelukast 4-5 mg) vs daily ICS (200 mcg) for 6 months	Children diagnosed with uncontrolled asthma	2400 (2.4 ± 1.2 years[5])	Need to step-up or step-down therapy according to GINA criteria	51.6% of children on ICS versus 16.7% on montelukast (p < 0.001) stepped-down therapy[6]<br><br>6.4% of children on ICS versus 32.1% on montelukast (p< 0.001) stepped-up therapy[6]
Fluticasone or montelukast in preschool wheeze: a randomized controlled trial. **Krawiec, 2015 [6]**	Single-centre, open-label, randomized trial	Daily LTRA (montelukast, 4 mg/day; n 23) vs daily ICS (fluticasone 2×50 or 2x100 mcg depending on age; n 23) vs placebo (n 24) for 12 weeks	Children between the 1st and 3rd episode of wheezing, not treated with montelukast, ICS, or systemic corticosteroids 2 months prior to admission	70 (6-36 months)	Wheezing episodes[7]<br><br>Hospitalizations[7]	40% of children experienced wheezing episodes during the 12 weeks treatment period with montelukast, 76% at one year.<br><br>10% of children required hospitalization during the 12 weeks treatment period with montelukast, 23% at one year.[8]
Healthcare utilization in infants and toddlers with asthma-like symptoms **Levine, 2019 [7]**	Retrospective study	Daily ICS (n 20316) or LTRA (n 5792)	Asthma-like symptoms and controller therapy before age of 3 years	262900 wheezers (<3 years),<br>26108 (10%) received controller therapy:<br>20 316 (78%) received ICS with or without LTRA, 5792 (22%) received LTRA alone	Number of respiratory-related doctor visits<br><br>Hospital admissions<br><br>Pneumonia doctor diagnosis<br>Chest radiographs	Outcomes before vs after 3 months of LTRA treatment:<br>Doctor visits<br>22 965 vs 15 332 p<0.001<br><br>Hospital admissions<br>522 vs 367 p<0.001<br><br>Pneumonia diagnosis<br>746 vs 601 p=0.004<br>Chest X-rays<br>2 008 vs 846 <0.001

					Additional medication	Bronchodilators<br>8 726 vs 6 447 <0.001<br>Systemic steroids<br>8 817 vs 5 989 p<0.001<br>Macrolide<br>7 251 vs 6 181 p<0.001<br>Penicillin<br>4 182 vs 3 308 p<0.001<br><br>Outcomes before vs after 12 months of LTRA treatment:
					Number of respiratory-related doctor visits	Doctor visits<br>8 518 vs 7 530 p<0.001
					Hospital admissions	Hospital admissions<br>67 vs 147 p<0.001
					Pneumonia doctor diagnosis	Pneumonia diagnosis<br>206 vs 150 p=0.058
					Chest radiographs	Chest X-rays<br>446 vs 55 p<0.001
					Additional medication	Bronchodilators<br>3 330 vs 2 134 p<0.001<br>Systemic steroids<br>2 336 vs 1 638 p<0.001<br>Macrolide<br>2 300 vs 2 049 p<0.001<br>Penicillin<br>1 402 vs 845 p=.045
Role of montelukast in multitrigger wheezers attending chest clinic in Punjab, India.<br>**Kahlon, 2021 [8]**	Cohort study	Daily LTRA (montelukast, 4 mg daily) for 3 months	MTWheezers	139 (6 months-5 years)	Symptoms control	Controlled at 1 and 3 months: 96 and 94 Partially controlled at 1 and 3 months: 11 and 8<br>Uncontrolled/Failure at 1 and 3 months: 32 and 37 [9]

[a]Leukotrien antagonists; [b]Fraction of exhaled nitric oxide; [c]Short acting beta-agonists; [d]Emergency room; [e]Inhaled corticosteroids

[1]The authors suggested that the instructions and information given to the parents in the timing/use of rescue medication might have led to an increase of its use

[2]The study analysed a population between 2 to 14 years; but it divided the results between preschool and school-age children. In the table the results of the preschool age group are reported

[3]p-value refers to comparison of the results at week 4, 8, 12 with baseline

[4]p-value refers to comparison of the results at week 8, 12 with week 4

[5]Reported as mean and standard deviation
[6]After 6 months of therapy
[7]Outcomes were measured during the 3 months of treatment and at 1 year after randomization (no longer under daily therapy)
[8]The study did not compare the outcomes at different endpoints within the same therapy group, so no statistical analysis was available
[9]Results are given as number of patients

References

1. Kooi EM, Schokker S, Marike Boezen H, de Vries TW, Vaessen-Verberne AA, van der Molen T, Duiverman EJ. Fluticasone or montelukast for preschool children with asthma-like symptoms: Randomized controlled trial. Pulm Pharmacol Ther. 2008 Oct;21(5):798-804. doi: 10.1016/j.pupt.2008.06.004. Epub 2008 Jul 2. PMID: 18647656.
2. Moeller A, Lehmann A, Knauer N, Albisetti M, Rochat M, Johannes W. Effects of montelukast on subjective and objective outcome measures in preschool asthmatic children. Pediatr Pulmonol. 2008 Feb;43(2):179-86. doi: 10.1002/ppul.20753. PMID: 18085698.
3. Wu WF, Wu JR, Dai ZK, Tsai CW, Tsai TC, Chen CC, Yang CY. Montelukast as monotherapy in children with mild persistent asthma. Asian Pac J Allergy Immunol. 2009 Dec;27(4):173-80. PMID: 20232571.
4. Szefler SJ, Carlsson LG, Uryniak T, Baker JW. Budesonide inhalation suspension versus montelukast in children aged 2 to 4 years with mild persistent asthma. J Allergy Clin Immunol Pract. 2013 Jan;1(1):58-64. doi: 10.1016/j.jaip.2012.08.005. Epub 2012 Nov 8. PMID: 24229823.
5. Jehan N, Rehman MU, Zarkoon MH. To determine the efficacy of inhaled corticosteroids compared to montelukast in reducing exacerbation in uncontrolled asthma in children 6 months to 5 years. Pak J Med Health Sci. 2014;8:662–666
6. Krawiec M, Strzelak A, Krenke K, Modelska-Wozniak I, Jaworska J, Kulus M. Fluticasone or montelukast in preschool wheeze: a randomized controlled trial. Clin Pediatr (Phila). 2015 Mar;54(3):273-81. doi: 10.1177/0009922814550158. Epub 2014 Sep 22. PMID: 25246602.
7. Levine H, Leventer-Roberts M, Hoshen M, Mei-Zahav M, Balicer R, Blau H. Healthcare utilization in infants and toddlers with asthma-like symptoms. Pediatr Pulmonol. 2019 Oct;54(10):1567-1577. doi: 10.1002/ppul.24429. Epub 2019 Jul 12. PMID: 31298808.
8. Kahlon GK, Pooni PA, Bhat D, Dhooria GS, Bhargava S, Arora K, Gill KS. Role of montelukast in multitrigger wheezers attending chest clinic in Punjab, India. Pediatr Pulmonol. 2021 Aug;56(8):2530-2536. doi: 10.1002/ppul.25522. Epub 2021 Jun 8. PMID: 34102024.

Supplementary Material 2: GRADE method: quality of evidence and strength of recommendations.

Category; grade	Definition
**Strength of recommendation**	
A	Strong evidence for efficacy and substantial clinical benefit.<br>Strongly recommended
B	Strong/moderate evidence for efficacy, but only limited clinical benefit.<br>Generally recommended
C	Insufficient evidence for efficacy; optional
**Quality of evidence**	
I	Evidence ≥1 properly randomized, controlled trial
II	Evidence from ≥1 well-designed clinical trial without randomization;<br>or cohort of case-controlled analytic studies (preferably from >1 center);<br>or from multiple time-series;<br>or from dramatic results from uncontrolled experiments
III	Evidence from the opinions of respected authorities, based on clinical experience,<br>descriptive studies, or reports of expert committees
